# Diterpenoids and C_13_ Nor-Isoprenoid Identified From the Leaves and Twigs of *Croton yanhuii* Activating Apoptosis and Pyroptosis

**DOI:** 10.3389/fchem.2022.861278

**Published:** 2022-03-28

**Authors:** Yue-qian Li, Bo-lin Hou, Mei-jie Wang, Ru-yue Wang, Xiao-han Chen, Xu Liu, Dong-qing Fei, Zhan-xin Zhang, Er-wei Li

**Affiliations:** ^1^ School of Pharmacy, State Key Laboratory of Applied Organic Chemistry, Lanzhou University, Lanzhou, China; ^2^ State Key Laboratory of Mycology, Institute of Microbiology, Chinese Academy of Sciences, Beijing, China; ^3^ Institutional Center for Shared Technologies and Facilities, Institute of Microbiology, Chinese Academy of Sciences, Beijing, China; ^4^ University of Chinese Academy of Sciences, Beijing, China; ^5^ State Key Laboratory for Chemistry and Molecular Engineering of Medicinal Resources, Guangxi Normal University, Guilin, China

**Keywords:** Euphorbiaceae, *Croton* yanhuii, diterpenoid, C_13_ nor-isoprenoid, cell apoptosis, pyroptosis

## Abstract

*Croton yanhuii* (Family Euphorbiaceae) is an annual aromatic plant endemic to Yunnan Province, China, which yields an aromatic, spicy oil used as a flavoring and fragrance. The aim of the present study was to acquire secondary metabolites from the leaves and twigs of *C. yanhuii* and to evaluate their cytotoxic activity. Five new diterpenoids, croyanhuins A–E (**1**–**5**), and one new C_13_ nor-isoprenoid, croyanhuin F (**6**), were isolated from the leaves and twigs of *C. yanhuii*. Their structures and absolute configurations were determined by extensive spectroscopic methods (1D and 2D NMR, IR, and HRESIMS) and confirmed by electronic circular dichroism (ECD) spectra or single-crystal X-ray diffraction analysis. Among the new terpenoids, compounds **1** and **3** inhibited cell proliferation and viability in a dose- and time-dependent manner, whereas both induced cleavage of either caspase-3 or PARP-1 in the SW480 cell line. Additionally, we observed that Z-YVAD-FMK and Z-VAD-FMK, two caspase inhibitors, inhibited the compound-dependent cell viability loss, suggesting that either of them can induce pyroptosis and caspase-dependent apoptosis. These biological assay results revealed that compounds **1** and **3** induce different kinds of programmed cell death in SW480 cells.

## Graphical Abstract

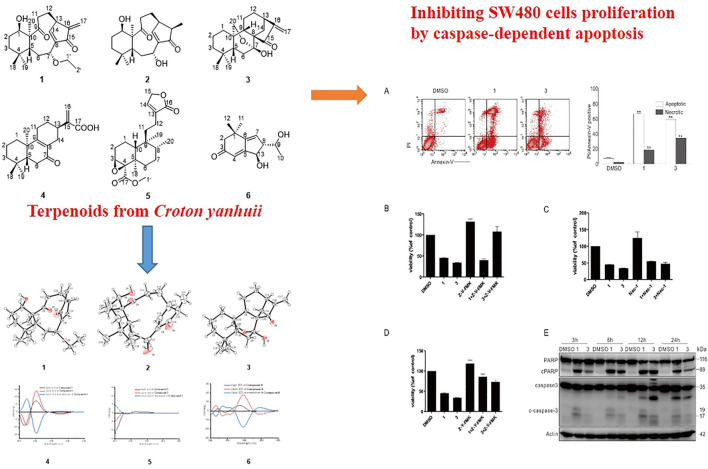

## Introduction

**FIGURE 1:**
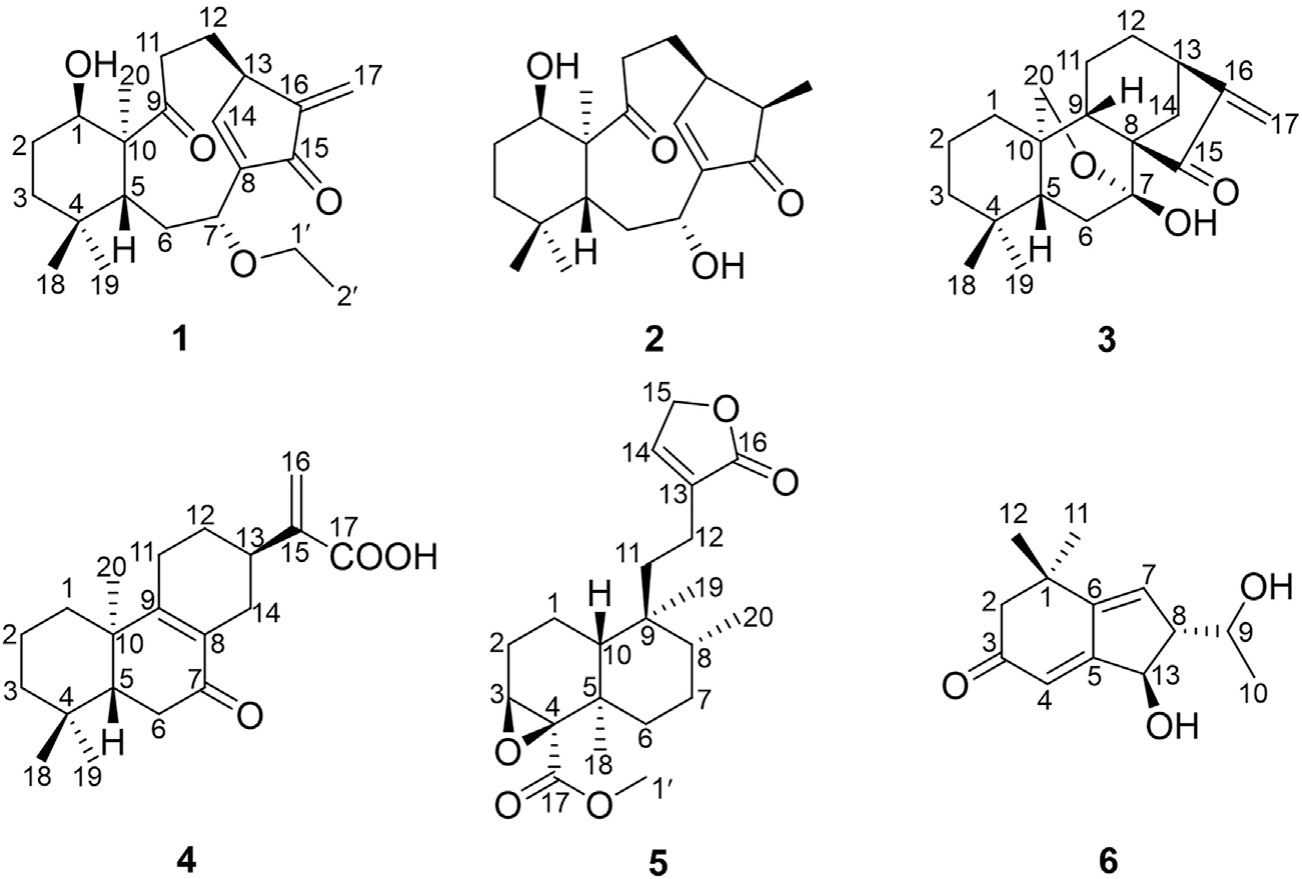
Structures of compounds **1**–**6** isolated from *Croton yanhuii.*

The plants of genus *Croton* belong to Euphorbiaceae family Crotonoideae subfamily, which contains about 1,300 species distributed in tropical and subtropical regions of the world (Berry et al., 2005). Many *Croton* species have been used as folk medicines in South America, North America, and Africa for the treatment of many diseases such as diabetes, high blood cholesterol levels, and leukemia (Salatino et al., 2007). In China, the seeds of *C. tiglium* are well known as “Ba-Dou”, a traditional Chinese medicine (TCM) that is widely used as an herb for the treatment of gastrointestinal disorders, and is purgative and antidermatophytic (Tsai et al., 2004; Wang et al., 2008; Lin et al., 2016). The essential oil extracted from the seeds of *C. tiglium* shows anti-tumor activity (Niu et al., 2020). Previous secondary metabolite investigations of this genus revealed that diterpenoids were the main ingredients (Xu et al., 2018), including clerodane (Fattorusso et al., 2002), tigliane (Cui et al., 2019), kaurane (Kuo et al., 2013), labdane (Yang et al., 2016), and pimarane (Isyaka et al., 2020), which have a wide range of biological activities, such as cytotoxic, anti-inflammatory, and anti-microbial (Morales et al., 2005; Kuo et al., 2007; Leite et al., 2017). Aside from those abovementioned, alkaloids (Novello et al., 2016), flavonoids (Cruz et al., 2020), phenylpropanoids (Abreu et al., 2020), and other terpenoids like sesquiterpenoids are present in the genus *Croton*. *C. yanhuii* is an annual aromatic plant endemically distributed in Yunnan Province of China (Flora of China Editorial Committee, 1996), which yields an aromatic, spicy oil used as a flavoring and fragrance. *C. yanhuii* is more commonly used as a tobacco additive by the local residents. There are a lot of studies on the genus *Croton* but a few on *C. yanhuii.* So far, only clerodane diterpenoids have been isolated from *C. yanhuii* (Sun et al., 2014; Li et al., 2020; Zou et al., 2021). In our present phytochemical investigation of this species, five new diterpenoids (**1**–**5**) and one new C_13_ nor-isoprenoid (**6**) (Figure 1) were isolated from the leaves and twigs of *C. yanhuii*. Their structures were elucidated by extensive spectroscopic interpretation. In the biological screenings, compounds **1** and **3** have a wonderful result achieved by decreasing cellular proliferation. Further exploration was implemented to uncover the mode of cell death that is caspase-dependent apoptosis. We herein present the isolation, structural elucidation, and biological evaluation of these new compounds.

## Results and Discussion

### Structural Elucidation

**FIGURE 2:**
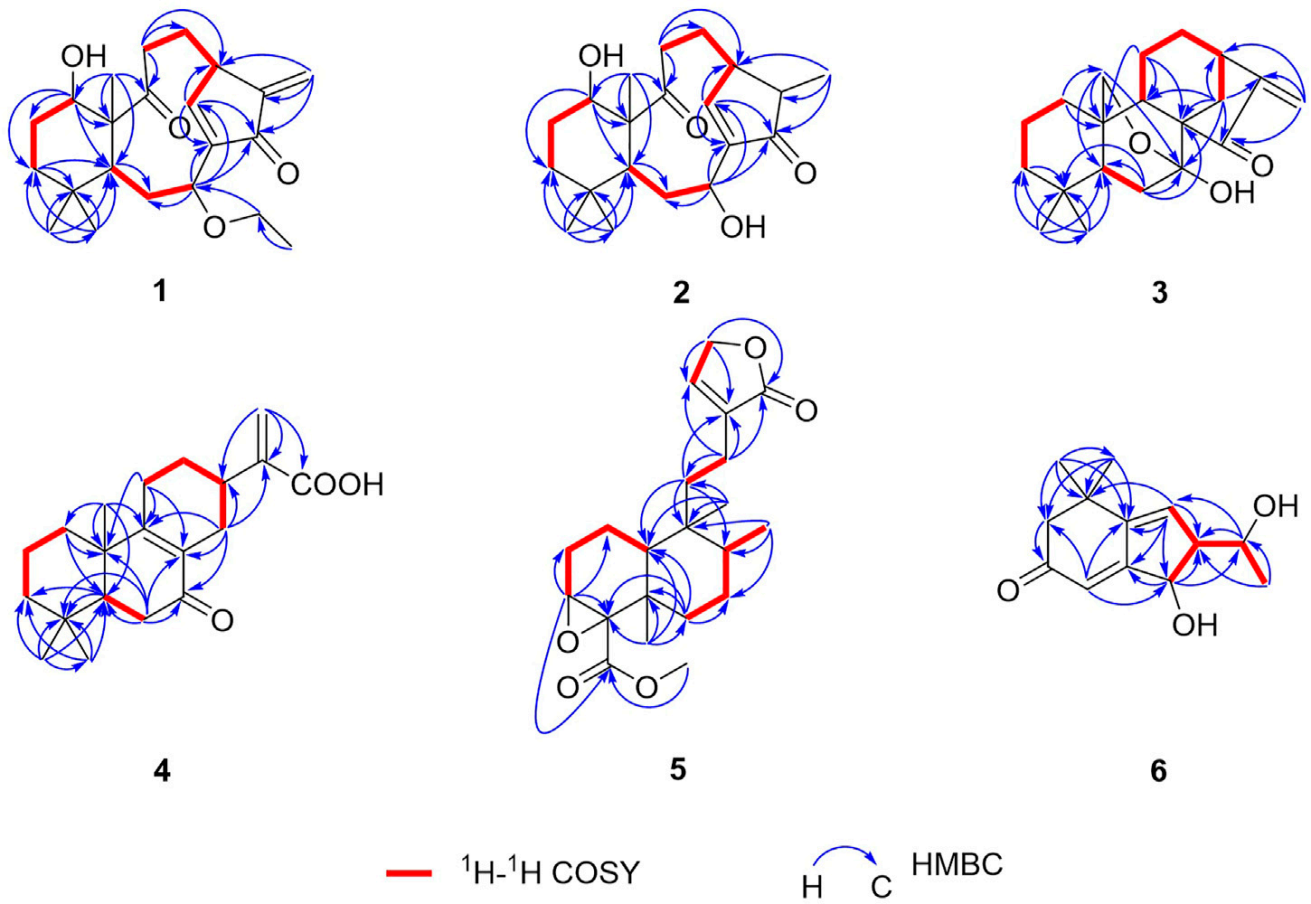
Key ^1^H-^1^H COSY and HMBC correlations of **1**–**6**.

Croyanhuin A (**1**) was obtained as colorless needle crystals. The molecular formula of **1** was established as C_22_H_32_O_4_ based on the HRESIMS (M + H)^+^ ion at *m/z* 361.2378 (calcd. for C_22_H_33_O_4_, 361.2379), requiring seven indices of hydrogen deficiency. The IR absorption bands at 3,491 and 1,685 cm^−1^ indicated the presence of hydroxyl and carbonyl functionalities. The ^1^H NMR spectrum of **1** (Table 1) revealed four methyl signals, including three singlet ones at *δ*_H_ 0.97 (s, H_3_-20), 1.00 (s, H_3_-19), and 1.09 (s, H_3_-18), and one triplet one at *δ*_H_ 1.13 (t, *J* = 7.0 Hz, H_3_-2′). One oxygenated methylene group at *δ*_H_ 3.29 (dq, *J* = 14.0, 7.0 Hz, H_2_-1′a) and 3.34 (dq, *J* = 14.0, 7.0 Hz, H_2_-1′b), two oxygenated methine groups at *δ*_H_ 3.60 (brs, H-1) and 4.32 (dd, *J* = 12.0, 4.5 Hz, H-7), an exocyclic methylene at *δ*_H_ 5.43 (s, H-17a) and 6.12 (s, H-17b), and one olefinic proton at *δ*_H_ 7.20 (d, *J* = 2.5 Hz, H-14) were also observed in the ^1^H NMR spectrum. The ^13^C NMR spectrum (Table 2) displayed that **1** possessed 22 carbon atoms that were categorized by HSQC experiment into four methyls, seven methylenes [including one oxygenated methylene at *δ*_C_ 63.9 (C-1′) and one olefinic methylene at *δ*_C_ 116.4 (C-17)], five methines [including two oxygenated methines at *δ*_C_ 71.3 (C-1) and 71.2 (C-7), and one olefinic methine at *δ*_C_ 160.3 (C-14)], and six non-protonated carbons [including two carbonyl carbons at *δ*_C_ 214.2 (C-9) and 196.0 (C-15), and two olefinic carbons at *δ*_C_ 147.1 (C-8) and 146.4 (C-16)]. From the above analyses, two carbonyls and two double bonds counted for four of seven indices of hydrogen deficiency, which required that **1** maintained a tricyclic skeleton. The 1D NMR spectroscopic data of **1** were similar with the known 8,9-*seco*-*ent*-kaurane diterpenoid, kongeniod A (Shi et al., 2018), and the main difference was that one methoxyl group in kongeniod A was replaced by one ethoxyl group in **1**. The chemical shift values of H_2_-1′ and H_3_-2′, and their coupling relationship showed an ethoxyl group presented in the structure of **1**, which was further confirmed by the ^1^H-^1^H COSY correlation of H_2_-1′ with H_3_-2′. By detailed 2D NMR analysis, the ethoxyl group was assigned to C-7, on the basis of its HMBC correlation from H_2_-1′ to C-7 (Figure 2).

**TABLE 1:** ^1^H NMR (500 MHz) data of compounds **1**–**6** (*δ* in ppm, *J* in Hz).

Position	1^a^	2^b^	3^b^	4^a^	5^b^	6^c^
1	3.60, brs	3.56, brs	α 1.29. m	α 1.24, m	α 1.58, m	—
			β 0.98, m	β 1.82, m	β 1.73, m	
2	α 1.98, m	α 1.92, m	α 1.40, m	α 1.56, m	α 1.81, m	α 2.27, d (16.0)
	β 1.48, m	β 1.51, m	β 1.40, m	β 1.65, m	β 2.13, m	β 2.38, d (16.0)
3	α 1.24, m	α 1.15, m	α 1.46, m	α 1.20, m	3.34, brd (3.5)	—
	β 1.75, m	β 1.92, m	β 1.13, td (12.5, 4.5)	β 1.46, m		
4	—	—	—	—	—	5.92, brs
5	1.41, dd (6.0, 1.5)	1.52, m	1.39, m	1.69, dd (14.0, 4.0)	—	—
6	α 1.89, ddd (13.5, 6.0, 4.0)	α 1.78, m	α 1.62, dd (13.0, 6.5)	α 2.53, dd (17.5, 4.0)	α 1.89, m	—
	β 1.26, m	β 1.13, m	β 3.05, t (13.0)	β 2,38, dd (17.5, 14.0)	β 1.52, m	
7	4.32, dd (12.0, 4.5)	4.47, dd (12.0, 4.5)	—	—	α 1.52, m	6.34, t (2.0)
					β 1.31, m	
8	—	—	—	—	1.53, m	2.67, m
9	—	—	1.40, m	—		3.79, quint (6.5)
10	—	—	—	—	1.24, t (4.0)	1.32, d (6.5)
11	α 2.03, m	α 2.12, m	α 1.70, m	α 2.40, m	1.70, m	1.17, s
	β 2.60, m	β 2.36, m	β 1.33, m	β 2.26, m	1.55, m	
12	α 2.61, m	α 2.35, m	α 2.28, m	α 1.57, m	2.13, m	1.28, s
	β 1.74, m	β 1.64, m	β 1.37, m	β 1.85, m		
13	3.60, brs	3.12, m	2.97, dd (9.5, 4.5)	2.69, m	—	4.60, dd (5.0, 3.0)
14	7.20, d (2.5)	7.18, d (3.5)	α 2.11, brd (12,5)	α 2.00, m	7.41, quint (2.0)	—
			β 1.92, dd (12.5, 5.0)	β 2.71, m		
15	—	—	—	—	4.79, q (2.0)	
16	—	2.42, m	—	6.36, s	—	—
				5.60, s		
17	6.12, s	1.06, d (7.0)	5.72, s	—	—	—
	5.43, s		5.28, s			
18	1.09, s	1.05, s	1.07, s	0.94, s	1.18, s	—
19	1.00, s	0.97, s	0.86, s	0.89, s	1.04, s	—
20	0.97, s	0.92, s	4.09, dd (10.0, 2.0)	1.13, s	0.85, d (6.5)	—
			3.86, dd (10.0, 2.0)			
1′	3.34, dq (14.0, 7.0)	—	—	—	3.71, s	—
	3.29, dq (14.0, 7.0)					
2′	1.13, t (7.0)	—	—	—	—	—
1-OH	—	3.75, d (3.0)	—	—	—	—
7-OH	—	3.87, s	—	—	—	—

a Measured in CDCl_3_.

b Measured in (CD_3_)_2_CO.

c Measured in CD_3_OD.

**TABLE 2:** ^13^C NMR (125 MHz) data of compounds **1**–**6** (*δ* in ppm).

Position	1^a^	2^b^	3^b^	4^a^	5^b^	6^c^
1	71.3, CH	71.3, CH	31.0, CH_2_	36.2, CH_2_	16.9, CH_2_	35.4, C
2	27.9, CH_2_	28.4, CH_2_	19.4, CH_2_	18.7, CH_2_	22.8, CH_2_	52.2, CH_2_
3	33.8, CH_2_	34.8, CH_2_	41.6, CH_2_	41.1, CH_2_	59.2, CH	202.5, C
4	34.8, C	35.1, C	34.4, C	33.3, C	65.0, C	118.3, CH
5	38.4, CH	38.7, CH	50.0, CH	50.0, CH	34.4, C	171.6, C
6	34.6, CH_2_	38.6, CH_2_	32.6, CH_2_	35.5, CH_2_	37.4, CH_2_	149.5, C
7	71.2, CH	64.3, CH	96.1, C	199.7, C	28.6, CH_2_	136.1, CH
8	147.1, C	147.3, C	57.5, C	129.2, C	38.2, CH	62.3, CH
9	214.2, C	213.9, C	51.3, CH	166.7, C	39.9, C	69.4, CH
10	57.3, C	58.1, C	36.7, C	39.7, C	44.0, CH	22.1, CH_3_
11	36.1, CH_2_	37.5, CH_2_	16.9, CH_2_	23.8, CH_2_	36.9, CH_2_	27.9, CH_3_
12	25.9, CH_2_	21.7, CH_2_	30.7, CH_2_	27.0, CH_2_	19.0, CH_2_	28.7, CH_3_
13	42.6, CH	43.0, CH	34.7, CH	33.2, CH	134.4, C	75.8, CH
14	160.3, CH	162.0, CH	25.7, CH_2_	27.5, CH_2_	146.2, CH	—
15	196.0, C	209.6, C	204.9, C	143.1, C	71.0, CH_2_	—
16	146.4, C	45.2, CH	155.4, C	125.7, CH_2_	174.8, C	—
17	116.4, CH_2_	10.4, CH_3_	113.9, CH_2_	171.7, C	170.4, C	—
18	33.9, CH_3_	23.7, CH_3_	21.0, CH_3_	21.4, CH_3_	29.2, CH_3_	—
19	23.5, CH_3_	34.1, CH_3_	32,8, CH_3_	32.5, CH_3_	19.4, CH_3_	—
20	17.8, CH_3_	18.2, CH_3_	66.6, CH_2_	18.6, CH_3_	16.9, CH_3_	—
1ʹ	63.9, CH_2_	—	—	—	51.9, CH_3_	—
2ʹ	15.4, CH_3_	—	—	—	—	—

a Measured in CDCl_3_.

b Measured in (CD_3_)_2_CO.

c Measured in CD_3_OD.

**FIGURE 3:**
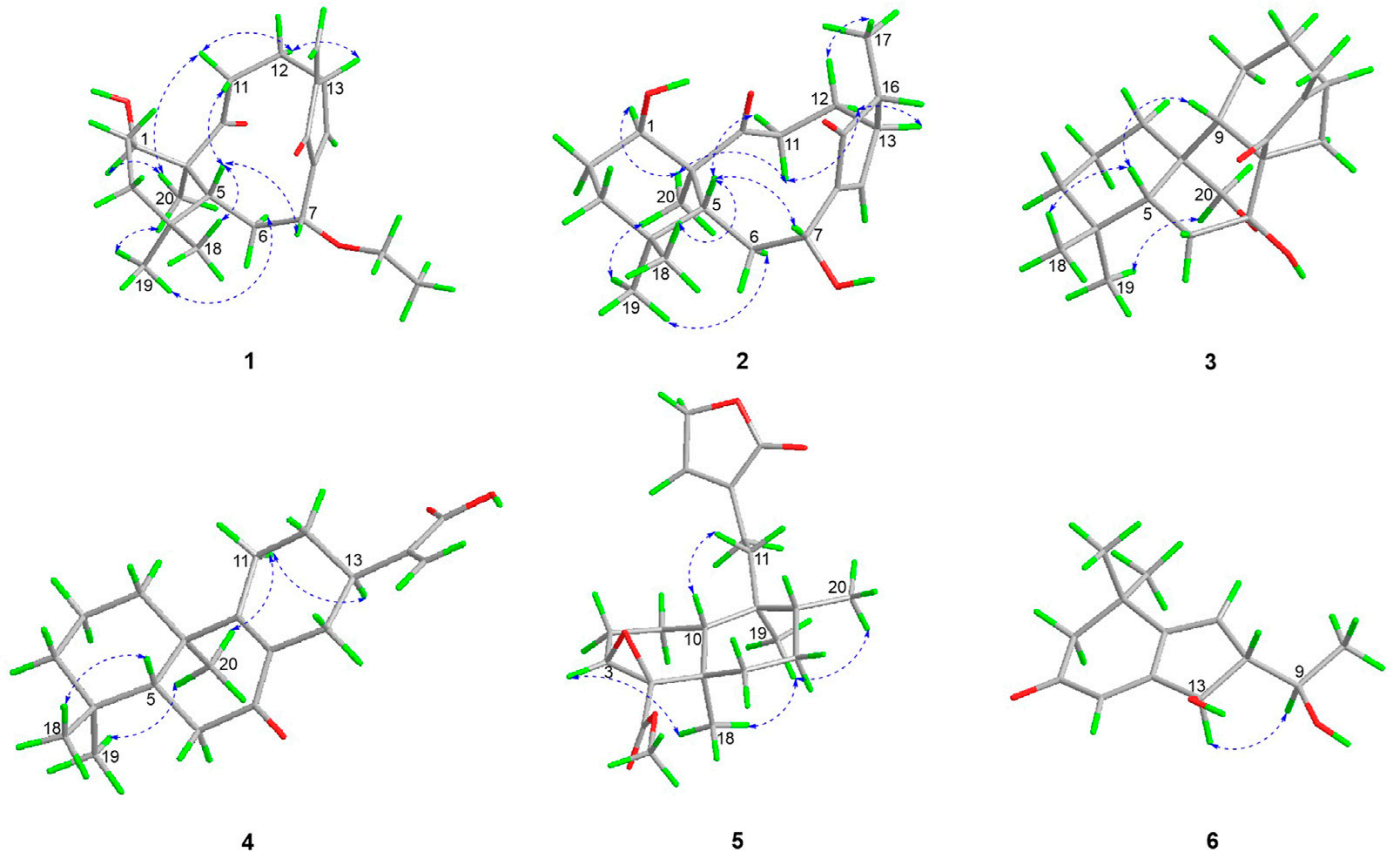
Key ROESY correlations of **1**–**6**.

**FIGURE 4:**
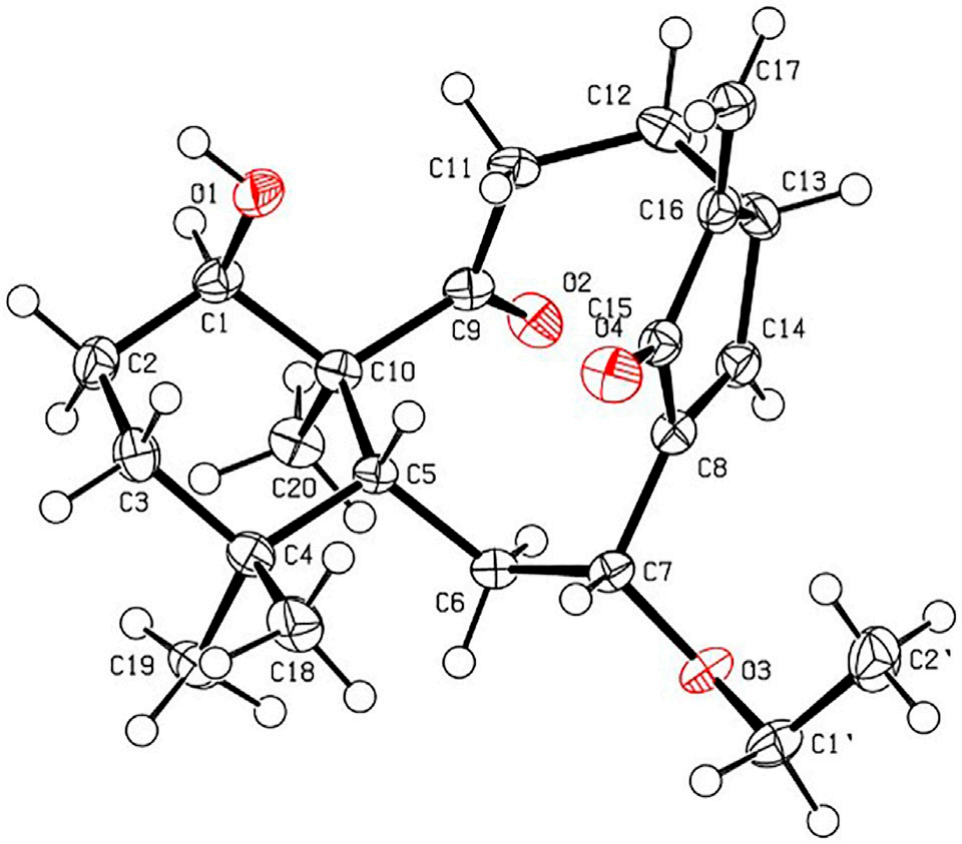
X-ray ORTEP drawing of **1** (with a thermal ellipsoid probability of 30%).

The relative stereochemistry of **1** was confirmed by careful analysis of the ROESY data (Figure 3). The key ROESY correlations of H_3_-20/H-1, H_3_-20/H_3_-19, H_3_-19/H-6α, H_3_-20/H-11α, H-11α/H-12α, and H-12α/H-13 indicated that these protons were cofacial and were assigned as α-oriented. Meanwhile, the ROESY correlations of H_3_-18/H-5, H-5/H-7, and H-5/H-11β revealed that these protons were on the opposite side and were assigned as β-oriented. The X-ray diffraction experiment (Figure 4) with Cu Kα radiation further corroborated the planar structure of **1** and fully determined its absolute configuration to be 1*R*, 5*R*, 7*R*, 10*S*, and 13*R*.

Croyanhuin B (**2**) was obtained as colorless needle crystals. The molecular formula of **2** was established to be C_20_H_30_O_4_ by HRESIMS data [*m/z* 335.2227 (M + H)^+^, calcd. for C_20_H_31_O_4_, 335.2222], indicating six indices of hydrogen deficiency. The IR absorptions at 3,444 and 1,686 cm^−1^ implied the presence of hydroxyl and carbonyl group in **2**. Comparisons of the ^1^H and ^13^C NMR data of **2** (Tables 1 and 2) with those of **1** indicated that **2** was an 8,9-*seco*-*ent*-kaurane diterpenoid as **1**. The significant differences in ^13^C NMR data were the absence of one double bond and one ethoxyl group resonances in **2**, which implied that the double bond would be hydrogenated and the ethoxyl group at C-7 would be substituted by one hydroxyl group in **2**. The speculation was further supported by the HMBC correlations from H_3_-17 to C-13, C-15, and C-16, and from H-7 to C-6, C-8, C-14, and C-15 (Figure 2).

**FIGURE 5:**
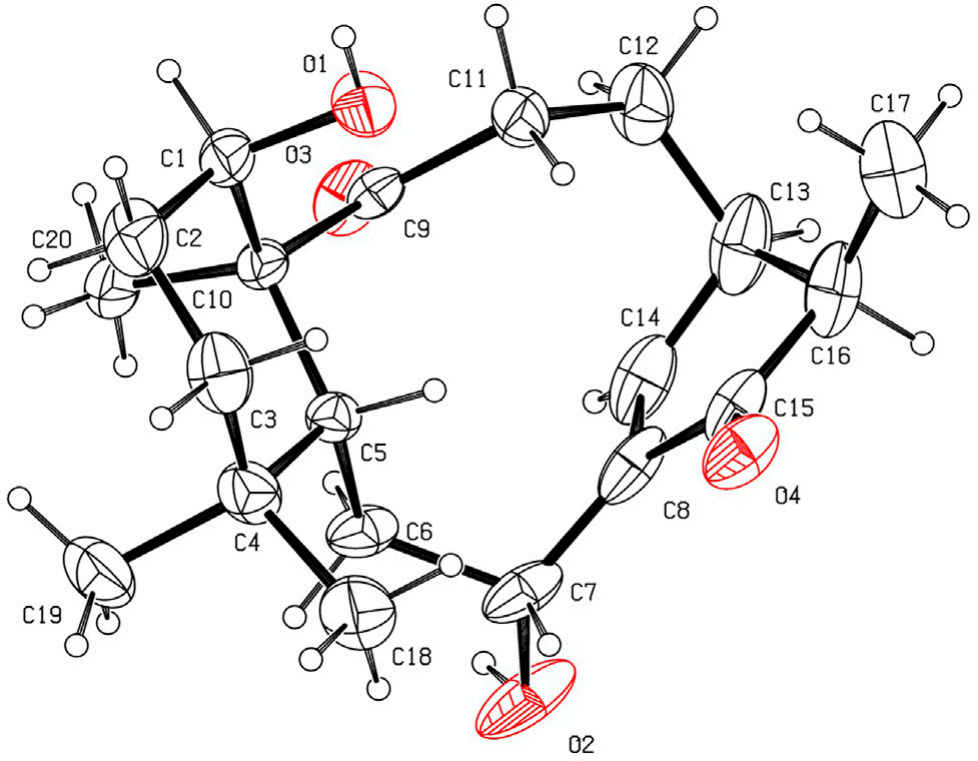
X-ray ORTEP drawing of **2** (with a thermal ellipsoid probability of 30%).

The relative configurations of **2** were determined to be identical to those of **1** by the similar ROESY correlations, except the correlation of H-12β/H_3_-17 in the ROESY spectrum, which assigned H_3_-17 to be β-oriented (Figure 3). The structure and absolute configuration of **2** were finally determined by the single-crystal X-ray diffraction experiment (Figure 5), which provided evidence for the absolute configuration of **2** as 1*R*, 5*R*, 7*R*, 10*S*, 13*R*, and 16*R*. Thus, the structure of **2** was finally deduced.

Croyanhuin C (**3**) was obtained as colorless needle crystals. Its molecular formula was determined to be C_20_H_28_O_3_ on the basis of the quasi-molecular ion peak at *m/z* 317.2116 (M + H)^+^ in its HRESIMS data. The IR absorption bands of **3** at 3,380 and 1,654 cm^−1^ indicated characteristic hydroxyl and conjugated carbonyl groups. Its ^1^H NMR spectrum (Table 1) revealed two singlet methyls at *δ*_H_ 0.86 (s, H_3_-19) and 1.07 (s, H_3_-18), a pair of terminal double bond protons at *δ*_H_ 5.28 (s, H-17a) and 5.72 (s, H-17b), and one oxygenated methylene at *δ*_H_ 3.86 (dd, *J* = 10.0, 2.0 Hz, H-20a) and 4.09 (dd, *J* = 10.0, 2.0 Hz, H-20b). The ^13^C NMR spectrum (Table 2), associated with HSQC experiment, exhibited 20 carbon resonances attributed to two methyls, nine methylenes [including one oxygenated carbon at *δ*_C_ 66.6 (C-20) and one olefinic carbon at *δ*_C_ 113.9 (C-17)], three methines, and six quaternary carbons [including one ketal carbon at *δ*_C_ 96.1 (C-7), one olefinic carbon at *δ*_C_ 155.4 (C-16), and one carbonyl carbon at *δ*_C_ 204.9 (C-15)]. Analysis of the ^1^H and ^13^C NMR spectroscopic data of compound **3** showed a structure related to the known compound serrin E (a 7,20-epoxy-*ent*-kaurane diterpenoid) (Wan et al., 2016), except for one oxygenated methine in serrin E that was replaced by a methylene in **3** at C-1 position. The planar structure of **3** was confirmed by the ^1^H-^1^H COSY correlations of H_2_-1/H_2_-2/H_2_-3 and the HMBC correlations from H_2_-1 to C-2, C-5, C-9, and C-10, and from H_2_-20 to C-1 (Figure 2).

**FIGURE 6:**
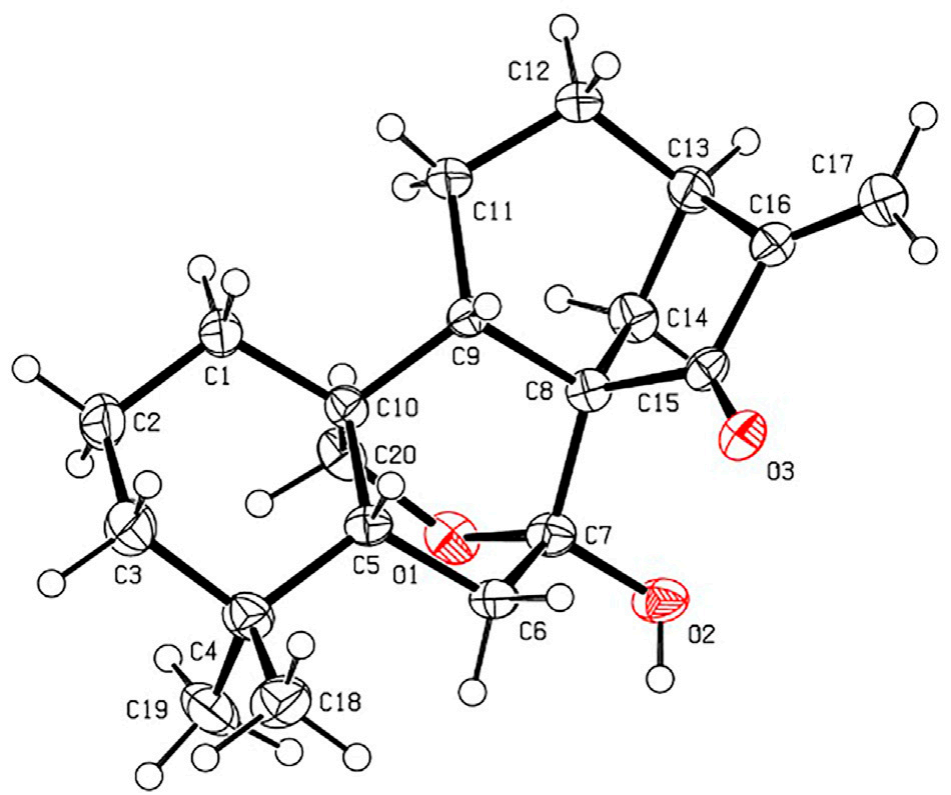
X-ray ORTEP drawing of **3** (with a thermal ellipsoid probability of 30%).

The relative configuration of **3** was established by analysis of its ROESY data (Figure 3). The ROESY correlations of H-5/H_3_-18 and H-5/H-9 demonstrated that H-5, H_3_-18, and H-9 were β-oriented, while the correlation of H_2_-20/H_3_-19 assigned H_2_-20 and H_3_-19 were α-oriented. Subsequently, a single-crystal X-ray diffraction experiment was conducted by Cu Kα radiation (Figure 6), which confirmed not only the above deduced planar structure of **3** but also its absolute configuration as 5*R*, 7*R*, 8*S*, 9*S*, 10*S*, and 13*R*. Thus, the structure of **3** was determined, which was further named croyanhuin C. The structure of compound **3** was recorded in SciFinder with a CAS number of 83,110-33-2, and only two references (Takeda et al., 1982; Xu, 1988) are available for the compound. However, **3** was not reported in the reference (Xu, 1988), and **3** was just reported as one basic skeleton in the structural elucidation process of similar compound lasiocarpanin, not actually isolated in the reference (Takeda et al., 1982). Although **3** was cited by SciFinder, it is still new.

Croyanhuin D (**4**) was isolated as a white amorphous powder. Its HRESIMS spectrum gave a pseudo-molecular ion peak at *m/z* 317.2116 [M + H]^+^ (calcd. for C_20_H_29_O_3_, 317.2117), corresponding to the molecular formula C_20_H_28_O_3_. The IR absorption band of **4** at 1,713 cm^−1^ exhibited the characteristic absorption of carbonyl groups. The ^1^H NMR spectroscopic data (Table 1) of **4** showed three methyl groups at *δ*_H_ 0.89 (s, H_3_-19), 0.94 (s, H_3_-18), and 1.13 (s, H_3_-20), and a pair of terminal double bond protons at *δ*_H_ 5.60 (s, H-16a) and 6.36 (s, H-16b). Additionally, its ^13^C NMR (Table 2) and HSQC spectra displayed the presence of 20 carbon resonances including three methyls, eight methylenes [including one olefinic carbon at *δ*_C_ 125.7 (C-16)], two methines, and seven non-protonated carbons [including one ketone carbonyl at *δ*_C_ 199.7 (C-7), one carboxyl at *δ*_C_ 171.7 (C-17), and three olefinic carbons at *δ*_C_ 129.2 (C-8), 143.1 (C-15) and 166.7 (C-9)]. As four of the seven degrees of unsaturation were accounted for one carbonyl group, one carboxyl group, and two double bonds, the remaining degrees of unsaturation required that **4** possessed a tricyclic skeleton. The characteristic chemical shift values of one carbonyl and two olefinic carbons mentioned before supported an α, β-unsaturated ketone at C-7, C-8, and C-9. The ^1^H-^1^H COSY spectrum revealed the presence of three spin systems of H_2_-1/H_2_-2/H_2_-3, H-5/H_2_-6, and H_2_-11/H_2_-12/H-13/H_2_-14. The HMBC correlations from H_3_-18 and H_3_-19 to C-3, C-4, and C-5, from H_3_-20 to C-1, C-5, C-9, and C-10, from H-5 to C-7, and the fragment of α, β-unsaturated ketone at C-7, C-8, and C-9 generated an A/B ring system. The HMBC correlations from H_2_-12 to C-9 and from H-13 to C-8 further linked the spin system of H_2_-11/H_2_-12/H-13/H_2_-14 *via* C-8 and C-9 to form a six-membered ring C. The HMBC correlations from H_2_-16 to C-13, C-17, and C-15 suggested that an acrylic acid moiety was assigned to C-13 *via* the 2-position carbon unambiguously (Figure 2).

**FIGURE 7:**
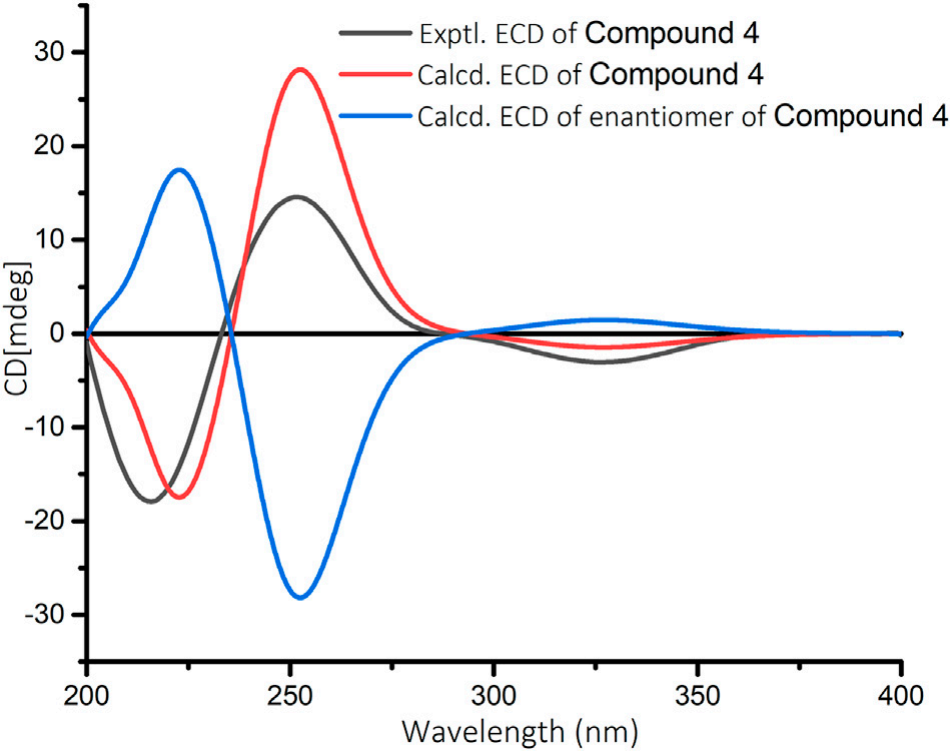
Calculated and experimental ECD spectra for compound **4** in MeOH.

The relative configuration of **4** was elucidated by ROESY spectrum. The ROESY correlations between H_3_-20/H_3_-19, H_3_-20/H-11α, and H-11α/H-13 indicated that H_3_-19, H_3_-20, H-11α, and H-13 were α-oriented, while correlation between H_3_-19/H-5 implied that H_3_-19 and H-5 were β-oriented (Figure 3). The absolute configuration of **4** (5*R*, 10*R*, 13*R*) was determined by comparing the experimental and calculated ECD data (Figure 7).

**FIGURE 8:**
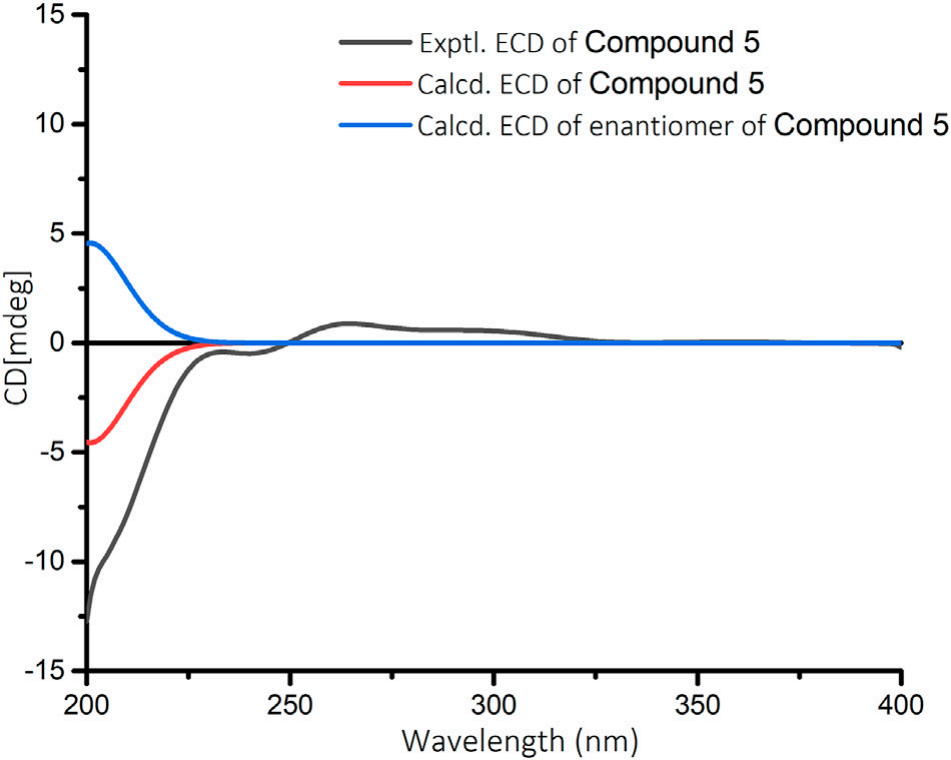
Calculated and experimental ECD spectra for compound **5** in MeOH.

Croyanhuin E (**5**) was obtained as a colorless oil, and its HRESIMS data exhibited a quasi-molecular ion peak at *m/z* 363.2171 (M + H)^+^ (calcd. for C_21_H_31_O_5_, 363.2171). The IR spectrum of **5** showed absorption bands for ester carbonyl groups at 1,748 cm^−1^. The ^1^H NMR data (Table 1) of **5** displayed signals for three methyls at *δ*_H_ 0.85 (d, *J* = 6.5 Hz, H_3_-20), 1.04 (s, H_3_-19), and 1.18 (s, H_3_-18), one methoxy group at *δ*_H_ 3.71 (s, H_3_-1′), one oxygenated methylene at *δ*_H_ 4.79 (q, *J* = 2.0 Hz, H_2_-15), one oxygenated methine at *δ*_H_ 3.34 (brd, *J* = 3.5 Hz, H-3), and one olefinic proton at *δ*_H_ 7.41 (quint, *J* = 2.0 Hz, H-14). The ^13^C NMR spectrum (Table 2) of **5**, associated with HSQC experiment, resolved 21 carbon resonances attributable to four methyls [including one oxygenated one at *δ*_C_ 51.9 (C-1′)], seven methylenes [including one oxygenated one at *δ*_C_ 71.0 (C-15)], four methines [including one olefinic one at *δ*_C_ 146.2 (C-14) and one oxygenated one at *δ*_C_ 59.2 (C-3)], and six non-protonated carbons [including one oxygenated sp^3^ hybrid quaternary carbon at *δ*_C_ 65.0 (C-4), two ester carbonyl carbons at *δ*_C_ 170.4 (C-17) and 174.8 (C-16), and one olefinic carbon at *δ*_C_ 134.4 (C-13)]. Detailed analysis of the 1D and 2D NMR spectra data of **5** showed that it had a clerodane diterpenoid skeleton. The ^1^H and ^13^C NMR spectra of **5** were very similar to those of the known tinotufolin F (Fukuda et al., 1994), with the difference being the replacement of a furan ring in tinotufolin F by an α, β-unsaturated γ-lactone ring in **5**, which was confirmed by the HMBC correlations from H_2_-15 to C-13, C-14, and C-16, and from H_2_-12 to C-11, C-13, C-14, and C-16 (Figure 2). In the ROESY spectrum (Figure 3), the cross-peaks from H_3_-18 to H-3, H_3_-18 to H_3_-19, and H_3_-19 to H_3_-20 indicated that H-3, H_3_-18, H_3_-19, and H_3_-20 were cofacial, and were assigned α-orientations. The ROESY correlation from H-10 to H_2_-11 suggested the β-orientations of H-10 and H_2_-11. On the basis of the relative configuration, the absolute configuration of **5** was established by ECD calculations. The results suggested that the calculated ECD spectrum matched well with the experimental data, revealing the absolute configuration to be (3*S*, 4*R*, 5*R*, 8*R*, 9*S*, 10*R*)-**5** (Figure 8).

Croyanhuin F (**6**) was obtained as a brown gum. Its HRESIMS data afforded a pseudo-molecular ion peak at *m/z* (M + H)^+^ 223.1327 (calcd. for C_13_H_19_O_3_, 223.1334), which was consistent with a molecular formula of C_13_H_18_O_3_ with five degrees of unsaturation. The IR spectrum showed the presence of absorption bands for hydroxyl and carbonyl groups at 3,398 and 1,644 cm^−1^, respectively. The ^1^H NMR data (Table 1) displayed three methyls at *δ*_H_ 1.17 (s, H_3_-11), 1.28 (s, H_3_-12), and 1.32 (d, *J* = 6.5 Hz, H_3_-10), two oxygenated methines at *δ*_H_ 3.79 (quint, *J* = 6.5 Hz, H-9) and 4.60 (dd, *J* = 5.0, 3.0 Hz, H-13), and two olefinic protons at *δ*_H_ 5.92 (brs, H-4) and 6.34 (t, *J* = 2.0 Hz, H-7). Its ^13^C NMR signals (Table 2), with the aid of HSQC spectra, exhibited 13 carbon resonances attributed to three methyls, one methylene, five methines [including two oxygenated carbons at *δ*_C_ 69.4 (C-9) and 75.8 (C-13), and two olefinic carbons at *δ*_C_ 118.3 (C-4) and 136.1 (C-7)], and four non-protonated carbons [including two olefinic carbons at *δ*_C_ 149.5 (C-6) and 171.6 (C-5), and one carbonyl carbon at *δ*_C_ 202.5 (C-3)]. The aforementioned NMR data suggested that compound **6** might be a C_13_ nor-isoprenoid with a two-ring system.

**FIGURE 9:**
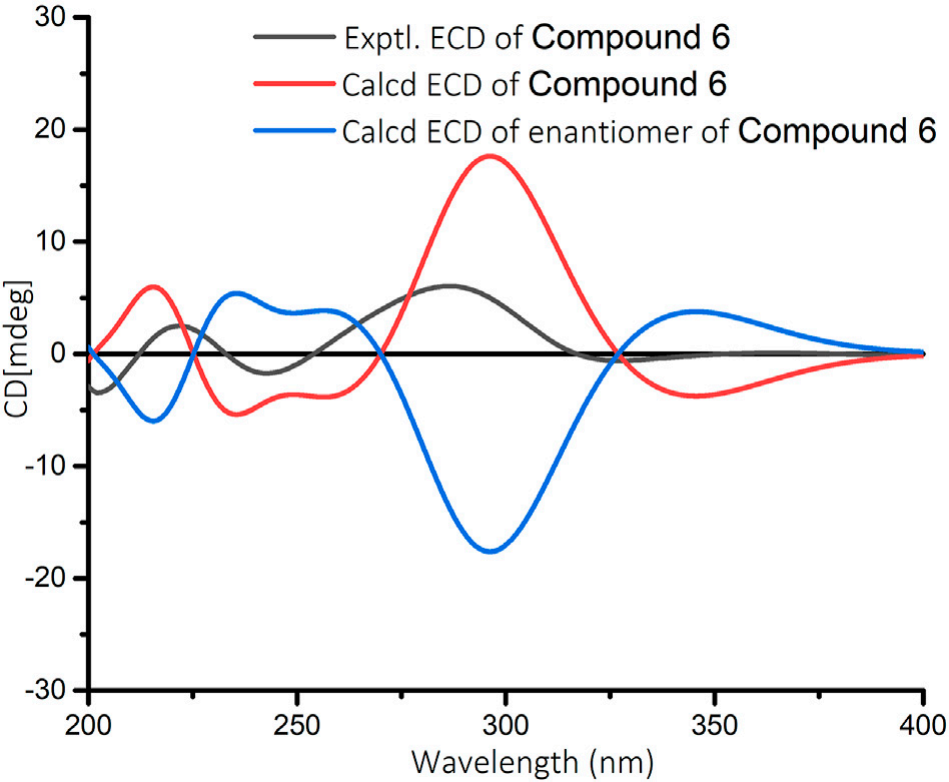
Calculated and experimental ECD spectra for compound **6** in MeOH.

In the ^1^H-^1^H COSY spectrum (Figure 2) of **6**, the correlations of H_3_-10/H-9/H-8 and H-7/H-8/H-13 revealed a fragment between C-7, C-8, C-9, C-10, and C-13. In the HMBC spectrum (Figure 2), the HMBC correlations from H_3_-11 and H_3_-12 to C-1, C-2, and C-6, from H_2_-2 to C-1, C-3, C-4, and C-6, and from H-4 to C-2 and C-6 demonstrated the presence of a cyclohex-2-en-1-one moiety (ring A) with CH_3_-11 and CH_3_-12 at C-1 in **6**. The HMBC correlations from H-7 to C-5, C-6, C-8, and C-13, from H-8 to C-6, C-7, and C-13, and from H-13 to C-4, C-5, and C-8 constructed a cyclopentene moiety (ring B) with a hydroxyl group at C-13, which fused with ring A *via* C-5 and C-6. Moreover, the HMBC correlations from H_3_-10 to C-8 and C-9, and from H-9 to C-7, C-8, C-10, and C-13, in combination with the aforementioned ^1^H-^1^H COSY correlations, showed that a 1-hydroxyethyl side chain was assigned to C-8. Hence, the planar structure of **6** with a 6/5 bicyclic core was deduced. The ROESY experiment (Figure 3) established the stereochemistry of **6**. ROESY correlation between H-13 and H-9 suggested that H-13 was α-orientation and H-8 was β-orientation. The absolute configuration of **6** was determined by comparing experimental and calculated ECD spectra predicted by time-dependent density-functional theory. The result showed that the experimental and calculated ECD spectra were in good agreement (Figure 9). Thus, the absolute configuration of **6** was assigned as 8*R* and 13*R*.

### Biological Activity

**FIGURE 10:**
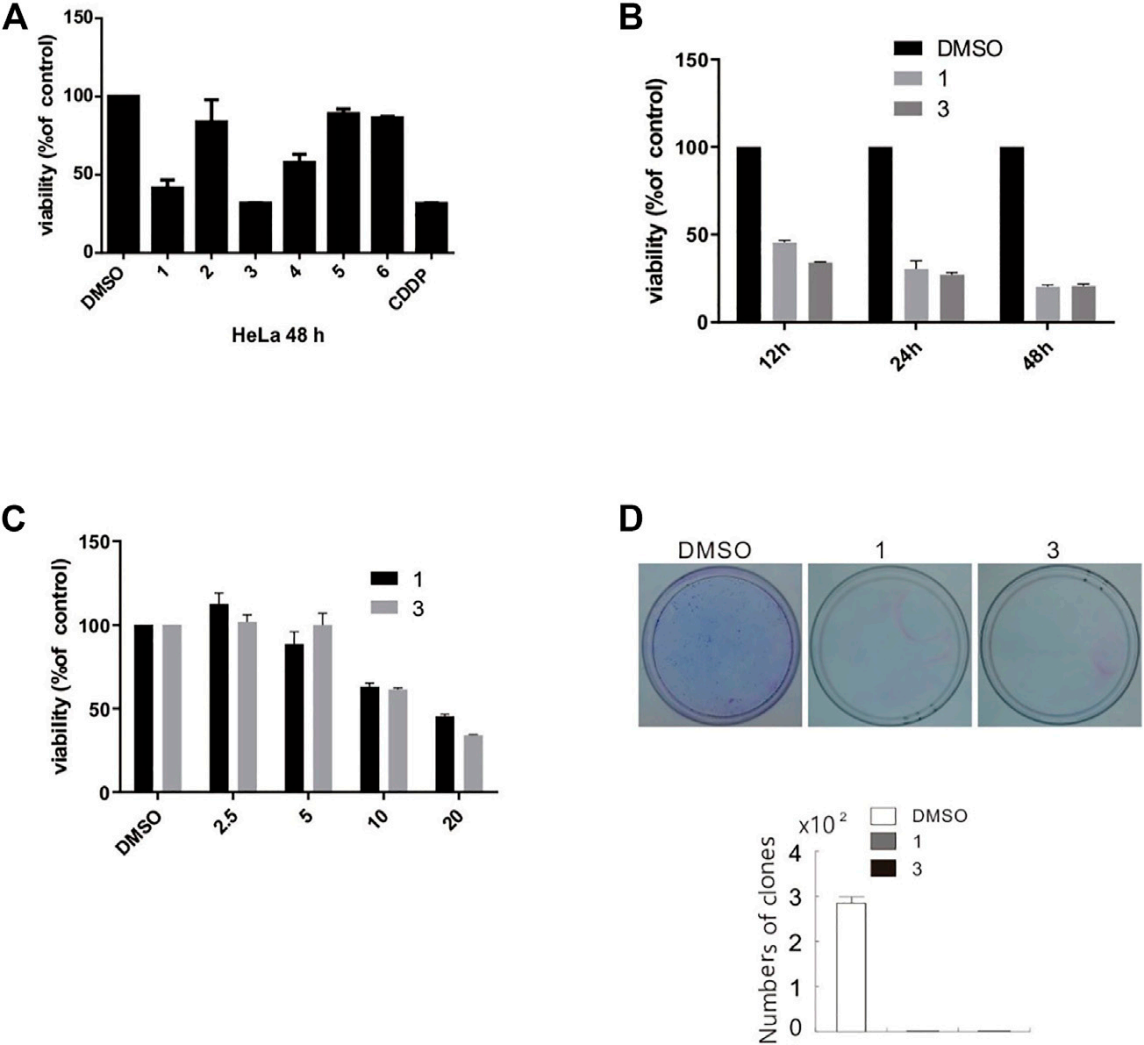
Compounds **1** and **3** markedly inhibited cell viability and proliferation. **(A)** HeLa cells were challenged with compounds **1–6** for 48 h, and detection of cell viability was carried out by MTS assay. **(B** and **C)** SW480 cells were treated with compounds **1** and **3** (2.5–25 μM) upon to 48 h, and detection of cell viability was carried out by MTS assay. **(D)** Colony formation assays in SW480 cells were performed in the presence of compounds **1** and **3** (20 μM) for 10 days. The image represented quantification of the signals (*n* = 3). For the results of histogram, the data of as mean ± S.D. presented and analyzed by *T*-test. Similar experiments repeated at least for three times.

MTS assay were carried out to detect the biological activities of compounds **1**–**6** at a dosage of 50 μM in HeLa cells. Among six compounds, compounds **1** and **3** exhibited markedly inhibitory activity (Figure 10A). Based on aforementioned results, we next used different concentrations (3.125, 6.25, 12.5, and 25 μM) of compounds **1** and **3** to detect the half maximal inhibitory concentration (IC_50_). As shown in Table 3, MTS assay was carried out in HeLa, SHSY5Y, SW480, A549, ACHN, and HepG2 cell lines, while *cis*-diaminedichloroplatinum (CDDP) was utilized as a positive control. Compared to other cell lines, compounds **1** and **3** displayed a stronger inhibition on cell viability in SW480 cells. Since compounds **1** and **3** were explored, the further activity evaluation in the SW480 cell line that is a human colon cancer. In MTS assay, compounds **1** and **3** inhibited the cell viability of SW480 cells in a time- and dose-dependent manner (Figures 10B,C), whereas their inhibitory effect was further confirmed by colony growth assays (Figure 10D). Taken together, aforementioned results indicated that compounds **1** and **3** were able to inhibit both cell viability and proliferation.

**TABLE 3:** Cytotoxic activity of compounds **1** and **3** (IC_50_, μM)^a^.

Compound/IC_50_	Hela	SHSY5Y	SW480	A549	ACHN	HepG2
Compound **1**	9.92 ± 3.01	21.81 ± 2.70	13.37 ± 3.63	10.2 ± 2.5	16.12 ± 3.18	23.24 ± 4.95
Compound **3**	8.27 ± 2.67	32.43 ± 3.15	10.37 ± 3.93	24.26 ± 2.06	29.07 ± 2.96	46.71 ± 3.03
CDDP	16.2 ± 2.88	18.48 ± 2.70	29.93 ± 1.66	NA^b^	20.36 ± 3.09	20.79 ± 2.23

a IC_50_ stands for mean ± SD.

b NA: not available. A549 has drug resistance to CDDP.

**FIGURE 11:**
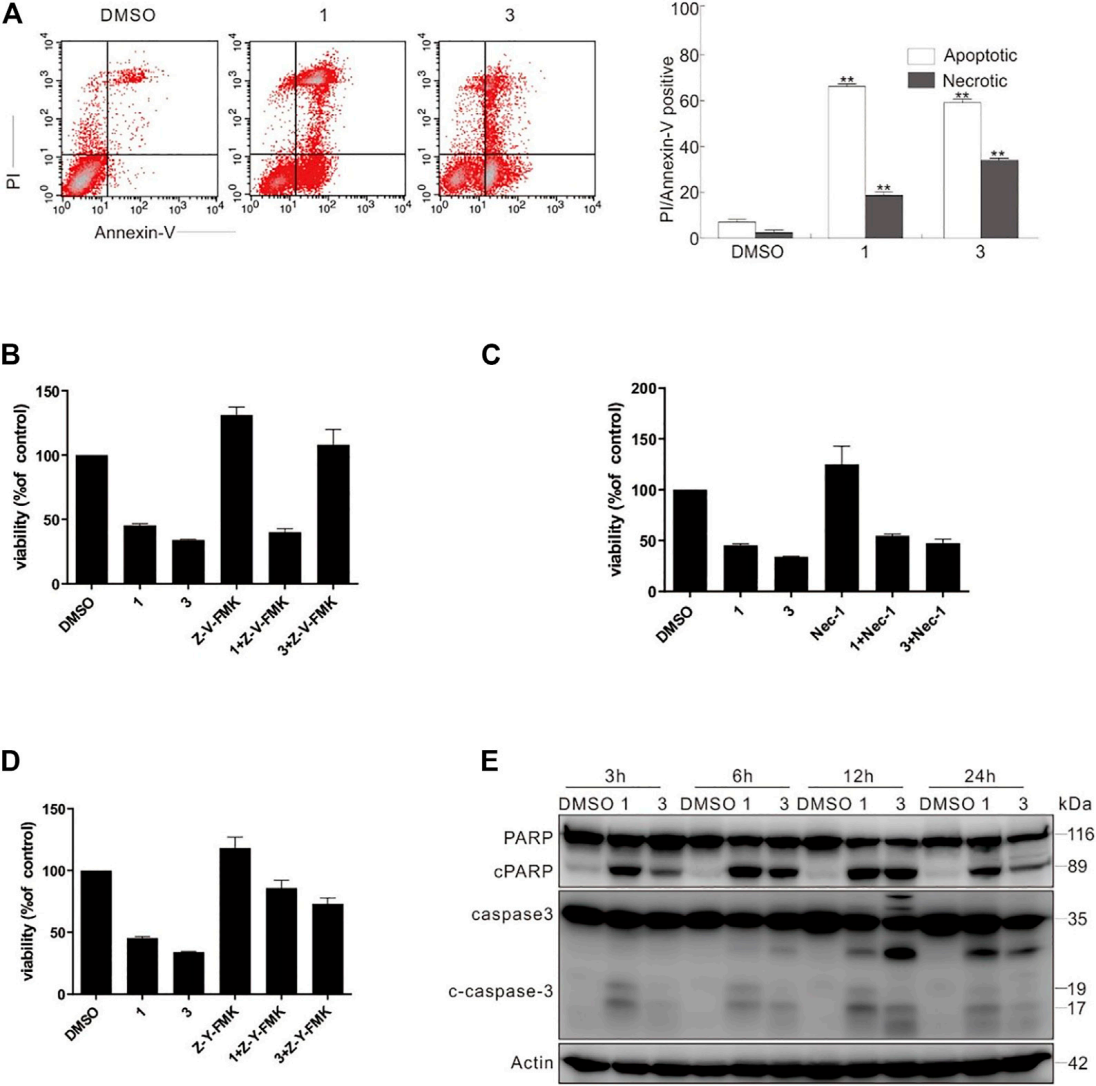
Compounds **1** and **3** activated caspase-dependent apoptosis. **(A)** Following treatment of SW480 cells with compounds **1** and **3** (20 μM) for 6 h, the induced apoptosis and necrosis were determined by flow cytometry. Apoptotic: AV-positive and PI-negative; necrotic: PI-positive. **(B–D)** SW480 cells were challenged with compounds **1** and **3** with or without Z-V-FMK (20 μM) or Nec1 (30 μM) or Z-Y-FMK (25 μM) for 12 h, and detection of cell viability was carried out by MTS assay. **(E)** The SW480 cells were treated with compounds **1** and **3** (20 μM) upon to 24 h, and then cell lysates were prepared and analyzed by immunoblotting using the indicated antibodies; actin was used as a loading control. The image represented quantification of the signals (*n* = 3). For the results of histogram, the data of as mean ± S.D. presented and analyzed by *T*-test. ^*^ *p* < 0.05 vs. control; ^*^^*^ *p* < 0.01 vs. control. Similar experiments were performed at least three times.

Through flow cytometry, compounds **1** and **3** could activate both apoptotic and necrotic cell death pathways (Figure 11A). To dig deep into the pathways of programmed cell death induced by compounds **1** and **3**, caspase inhibitors were employed in the following experiments. Interestingly, Z-VAD-FMK (Z-V-FMK), the pan-caspase inhibitor, almost totally rescued **3**-dependent but not **1**-induced cell viability loss (Figure 11B), whereas necrostatin 1 (Nec-1), which is a potent inhibitor of necroptosis, provided less protection than Z-V-FMK did in either **1** or **3** challenged cells (Figure 11C). Unexpectedly, Z-YVAD-FMK, a caspase 1 inhibitor, often used as pyroptosis inhibitor, markedly rescued both compounds **1**- and **3**-induced cell viability loss (Figure 11D), suggesting that either **1** or **3** can induce apoptosis and programmed necrotic cell death. Concerning Z-V-FMK’s failure of rescuing compound **1**-dependent cell viability loss, we posited that combination of compound **1** with Z-V-FMK might activate other types of cell death. The immunoblotting results revealed that the treatment of compounds **1** or **3** caused the cleavage of PARP-1 and caspase-3 (Figure 11E), indicating that these two compounds actually induced caspase-dependent apoptosis.

## Conclusion

In summary, six new terpenoids **1**–**6**, namely, five new diterpenoids (**1**–**5**) and one new C_13_ nor-isoprenoid (**6**), were purified from the leaves and twigs of *C. yanhuii*. The structures of the new compounds were characterized by using NMR, HRESIMS, ECD spectra, and X-ray diffraction. Among those, compounds **1** (8, 9-*seco*-*ent*-kaurane type diterpenoid) and **3** (7,20-epoxy-*ent*-kaurane type diterpenoid) are both kaurane-type diterpenoids, and could inhibit SW480 cell proliferation by caspase-dependent apoptosis. Compound **2** showed high similarity to **1**, but its bioactivity of inducing cell apoptosis is unsatisfactory. We surmise that exocyclic double bond and ethoxyl unit could be the essential functional groups. The bioactive kaurane-type diterpenes contained in *C. yanhuii* deserve further investigation for their anti-tumor potentials.

## Experimental Section

### General Experimental Procedures

Melting points were measured using an X-4 digital display micromelting point apparatus (Beijing Tech Instrument Co., Ltd., Beijing, China) and are uncorrected. Optical rotations were obtained on an Anton Paar MCP 200 Automatic Polarimeter (Anton Paar GmbH, Graz, Austria). IR and UV spectra were recorded on a Nicolet IS5 FT-IR spectrophotometer (Thermo Scientific, Madison, WI, United States) and a Thermo Genesys-10S UV-vis spectrophotometer (Thermo Scientific, Madison, WI, United States), respectively. ECD spectra were acquired on an Applied Photophysics Chirascan spectropolarimeter (Applied Photophysics Ltd., Leatherhead, United Kingdom). HRESIMS data were performed on an Agilent Accurate-Mass-Q-TOF LC/MS 6520 instrument (Agilent Technologies, Santa Clara, CA, United States). The NMR spectral data were measured on a Bruker Avance-500 MHz spectrometer (Bruker, Rheinstetten, Germany). Solvents including ethanol, methanol, petroleum ether (PE), acetone, dichloromethane, and ethyl acetate used for extraction and chromatographic separation were analytical grade. TLC was carried out on silica gel HSGF254 plates purchased from the Qingdao Marine Chemical Factory, and the spots were visualized by UV at 254 nm or spraying with 5% H_2_SO_4_ ethanol solution followed by heating. Silica gel (200–300 mesh Qingdao Haiyang Chemical Co., Ltd.), octadecylsilyl (ODS, 50 μm, YMC Co., Ltd.), MCI GEL CHP 20P (75–150 μm, Mitsubishi Chemical Ltd.), and Sephadex LH-20 (Amersham Biosciences) were used for column chromatography (CC). Semi-preparative HPLC was conducted with an Agilent 1200 HPLC system using a reversed-phase (RP) column (Reprosil-Pur Basic C18 column; 5 µm; 10 × 250 mm; detector: UV) with a flow rate of 2.2 ml/min.

### Plant Material

The leaves and twigs of *C. yanhuii* were collected in August 2019 from Guanlei Town, Mengla County, Xishuangbanna Prefecture, Yunnan Province, China, and identified by Dr. Jian-Yin Li (Lanzhou University). A voucher specimen (No. 20190623CY) was deposited at the Natural Product Laboratory, School of Pharmacy, Lanzhou University.

### Extraction and Isolation

The air-dried leaves and twigs of *C. yanhuii* (10 kg) were powdered and extracted four times with ethanol (4 × 100 L, 7 days each time) at room temperature. The solvent was evaporated to obtain a crude extract (527 g), which was suspended in H_2_O and partitioned with EtOAc and *n*-BuOH, successively. The EtOAc extract (180 g) was fractionated by silica gel (200–300 mesh) column chromatography (CC) eluted with PE-acetone (40:1, 20:1, 10:1, 5:1, 2:1, and 1:1, v/v, each about 15 L) to yield five fractions (Fr.A–Fr.E) on the basis of TLC analysis. The Fr.C (27 g) was chromatographed using MCI gel CC eluted with aqueous methanol in gradient (1:2 to 0:1, v/v) to obtain six subfractions (Fr.C1–Fr.C6). Fr.C2 (2.1 g) was separated by a Sephadex LH-20 column eluting with MeOH-CH_2_Cl_2_ (2:3, v/v) and further purified by semi-preparative RP-HPLC using MeOH-H_2_O (75:25, v/v, 2.2 ml/min) as the mobile phase to afford **1** (7.0 mg). Fr.C3 (3 g) was separated by silica gel CC eluted with PE-acetone (15:1, 10:1, 5:1, 1:1, and 0:1, v/v) to get five subfractions (Fr.C3.1–Fr.C3.5). Fr.C3.3 (190 mg) was further applied to silica gel CC eluting with CH_2_Cl_2_-acetone (100:1, 50:1, 25:1, and 0:1, v/v) to give four subfractions (Fr.C3.3.1–Fr.C3.3.4). Compounds **3** (1.1 mg) and **5** (3.5 mg) were isolated from Fr.C3.3.2 by semi-preparative RP-HPLC (MeOH-H_2_O 2:1 to 3:1, v/v, 2.2 ml/min). Fr.C3.5 (122 mg) was separated by a Sephadex LH-20 column eluted with MeOH to obtain nine subfractions (Fr.C3.5.1–Fr.C3.5.9). Fr.C3.5.4 (21 mg) was purified by a semi-preparative RP-HPLC (MeOH-H_2_O 2:1, v/v, 2.2 ml/min) to afford **4** (6.5 mg). Fr. D (32 g) was applied to an ODS MPLC CC and eluted with MeOH-H_2_O (1:9 to 1:0, v/v) to afford eight fractions (Fr.D1–Fr.D8). Fr. D2 (150 mg) was applied to a silica gel CC eluted with PE-acetone (10:1, 5:1, 1:1, and 0:1, v/v) to yield four subfractions (Fr.D2.1–Fr.D2.4). Compound **6** (7.0 mg) was isolated from Fr. D2.4 subjecting to Sephadex LH-20 CC eluted with MeOH and then subjecting to semi-preparative RP-HPLC (MeOH-H_2_O 1:3 to 2:3, v/v, 2.2 ml/min). Fr. D4 (170 mg) was chromatographed by a Sephadex LH-20 column eluting with MeOH and purified by semi-preparative RP-HPLC using MeOH-H_2_O (1:4 to 1:1, v/v, 2.2 ml/min) as the mobile phase to give **2** (3.0 mg).

Croyanhuin A (**1**): colorless needle crystals; mp 198–200°C; [*α*]_D_^20^ −41.42 (*c* 0.70, MeOH); UV (MeOH) *λ*_max_: 244 nm; IR (KBr) *v*_max_ cm^−1^: 3,491, 2,929, 1,685, 1,380, 1,096, 936 cm^−1^; see Table 1 for ^1^H NMR (500 MHz, CDCl_3_) data; see Table 2 for ^13^C NMR (125 MHz, CDCl_3_) data; HRESIMS *m/z* 361.2378 (M + H)^+^ (calcd. for C_22_H_33_O_4_, 361.2379), molecular formula was C_22_H_32_O_4_.

Crystallographic data of **1**. C_22_H_32_O_4_, *M*r = 360.47, orthorhombic, space group *P*2_1_2_1_2_1_, *a* = 8.1442 (1) Å, *b* = 14.7847 (2) Å, *c* = 15.8701 (2) Å, *α* = 90.00°, *β* = 90.00°, *γ* = 90.00°, *V* = 1910.91 (4) Å^3^, *T* = 150 K, *Z* = 4, *d* = 1.253 g/cm^3^, *μ*(Cu Kα) = 0.67 mm^−1^, *F* (000) = 784, crystal dimensions 0.21 × 0.03 × 0.01 mm were used for measurement on a Rigaku XtaLAB Synergy R, HyPix diffractometer with Cu Kα radiation (*λ* = 1.54184 Å). There was a total of 10,518 measured reflections and 3,777 independent reflections (*R*_int_ = 0.0423). The final *R*_1_ value was 0.0379 [*I* > 2σ (*I*)]. The final w*R* (*F* ^2^) value was 0.0989 [(*I* > 2σ (*I*)]. The final *R*_1_ value was 0.0393 (all data). The final w*R* (*F* ^2^) value was 0.0977 (all data). The goodness of fit on *F* ^2^ was 1.067. The Flack parameter was 0.04 (11). Crystallographic data for the structure of compound **1** have been deposited with the Cambridge Crystallographic Data Centre (deposition no. CCDC 2120825). Copies of these data can be obtained free of charge *via* www.ccdc.cam.ac.uk/conts/retrieving.html [or from the Cambridge Crystallographic Data Centre, 12 Union Road, Cambridge CB21EZ, U.K.; fax (+44) 1223–336-033; or deposit@ccdc.cam.uk].

Croyanhuin B (**2**): colorless needle crystals; mp 219–221°C; (*α*) −18.33 (*c* 0.30, MeOH); UV (MeOH) *λ*_max_: 236 nm; IR (KBr) *v*_max_ cm^−1^: 3,444, 2,946, 1,686, 1,462, 1,379, 1,029 cm^−1^; see Table 1 for ^1^H NMR [500 MHz, (CD_3_)_2_CO] data; see Table 2 for ^13^C NMR [125 MHz, (CD_3_)_2_CO] data; HRESIMS *m/z* 335.2227 (M + H)^+^ (calcd. for C_20_H_31_O_4_, 335.2222), molecular formula was C_20_H_30_O_4_.

Crystallographic data of **2**. C_20_H_30_O_4_, *M*r = 334.44, monoclinic, space group *P*2_1_, *a* = 9.3079 (7) Å, *b* = 10.9833 (6) Å, *c* = 9.6254 (8) Å, *α* = 90.00°, *β* = 111.847 (9)°, *γ* = 90.00°, *V* = 913.35 (12) Å^3^, *T* = 300 K, *Z* = 2, *d* = 1.216 g/cm^3^, *μ* (Cu Kα) = 0.66 mm^−1^, *F* (000) = 364, crystal dimensions 0.11 × 0.05 × 0.03 mm were used for measurement on a Rigaku XtaLAB Synergy R, HyPix diffractometer with Cu Kα radiation (*λ* = 1.54184 Å). There was a total of 15,567 measured reflections, and 3,497 independent reflections (*R*_int_ = 0.0802). The final *R*_1_ value was 0.0620 [*I* > 2σ (*I*)]. The final w*R* (*F* ^2^) value was 0.1787 [*I* > 2σ (*I*)]. The final *R*_1_ value was 0.0807 (all data). The final w*R* (*F* ^2^) value was 0.1673 (all data). The goodness of fit on *F* ^2^ was 1.077. The Flack parameter was −0.2 (3). Crystallographic data for the structure of compound **2** have been deposited with the Cambridge Crystallographic Data Centre (deposition no. CCDC 2143626). Copies of these data can be obtained free of charge *via* www.ccdc.cam.ac.uk/conts/retrieving.html [or from the Cambridge Crystallographic Data Centre, 12 Union Road, Cambridge CB21EZ, U.K.; fax (+44) 1223–336-033; or deposit@ccdc.cam.uk].

Croyanhuin C (**3**): colorless needle crystals; mp 170–171°C; [*α*]_D_^20^ −65.44 (*c* 0.11, MeOH); UV (MeOH) *λ*_max_: 220 nm; IR (KBr) *v*_max_ cm^−1^: 3,380, 3,010, 2,921, 1,654, 1,437, 1,406, 1,317, 1,020, 952 cm^−1^; see Table 1 for ^1^H NMR [500 MHz, (CD_3_)_2_CO] data; see Table 2 for ^13^C NMR [125 MHz, (CD_3_)_2_CO] data; HRESIMS *m/z* 317.2116 (M + H)^+^ (calcd. for C_20_H_29_O_3_, 317.2117), molecular formula was C_20_H_28_O_3_.

Crystallographic data of **3**. C_20_H_28_O_3_, *M*r = 316.42, orthorhombic, space group *P*2_1_2_1_2_1_, *a* = 7.35623 (13) Å, *b* = 10.9350 (2) Å, *c* = 20.7149 (5) Å, *α* = 90.00°, *β* = 90.00°, *γ* = 90.00°, *V* = 1,666.31 (6) Å^3^, *T* = 150 K, *Z* = 4, *d* = 1.261 g/cm^3^, *μ*(Cu Kα) = 0.66 mm^−1^, *F* (000) = 688, crystal dimensions 0.09 × 0.04 × 0.02 mm were used for measurement on a Rigaku XtaLAB Synergy R, HyPix diffractometer with Cu Kα radiation (*λ* = 1.54184 Å). There was a total of 9,156 measured reflections, and 3,337 independent reflections (*R*_int_ = 0.0536). The final *R*_1_ value was 0.0440 [*I* > 2σ (*I*)]. The final w*R* (*F* ^2^) value was 0.1147 [(*I* > 2σ (*I*)]. The final *R*_1_ value was 0.0492 (all data). The final w*R* (*F* ^2^) value was 0.1117 (all data). The goodness of fit on *F* ^2^ was 1.032. The Flack parameter was −0.01 (16). Crystallographic data for the structure of compound **3** have been deposited with the Cambridge Crystallographic Data Centre (deposition no. CCDC 2120826). Copies of these data can be obtained free of charge *via* www.ccdc.cam.ac.uk/conts/retrieving.html [or from the Cambridge Crystallographic Data Centre, 12 Union Road, Cambridge CB21EZ, U.K.; fax (+44) 1223–336-033; or deposit@ccdc.cam.uk].

Croyanhuin D (**4**): colorless needle crystals; mp 219-221°C; [*α*]_D_^20^ +8.31 (*c* 0.65, MeOH); UV (MeOH) *λ*_max_: 250 nm; IR (KBr) *v*_max_ cm^−1^: 2,931, 1713, 1,659, 1,455, 1,377, 1,262, 1,025 cm^−1^; see Table 1 for ^1^H NMR (500 MHz, CDCl_3_) data; see Table 2 for ^13^C NMR (125 MHz, CDCl_3_) data; HRESIMS *m/z* 317.2116 [M + H]^+^ (calcd. for C_20_H_29_O_3_, 317.2117), molecular formula was C_20_H_28_O_3_.

Croyanhuin E (**5**): colorless oil; [*α*]_D_^20^ −1.14 (*c* 0.35, MeOH); UV (MeOH) *λ*_max_: 215 nm; IR (KBr) *v*_max_ cm^−1^: 2,954, 2,872, 1748, 1,452, 1,388, 1,257, 1,069 cm^−1^; see Table 1 for ^1^H NMR [500 MHz, (CD_3_)_2_CO] data; see Table 2 for ^13^C NMR [125 MHz, (CD_3_)_2_CO] data; HRESIMS *m/z* 363.2171 (M + H)^+^ (calcd. for C_21_H_31_O_5_, 363.2171), molecular formula was C_21_H_30_O_5_.

Croyanhuin F (**6**): brown gum; [*α*]_D_^20^ −42.28 (*c* 0.70, MeOH); UV (MeOH) *λ*_max_: 292nm; IR (KBr) *v*_max_ cm^−1^: 3,398, 2,965, 1,644, 1,384, 1,277, 1,129, 888 cm^−1^; see Table 1 for ^1^H NMR (500 MHz, CD_3_OD) data; see Table 2 for ^13^C NMR (125 MHz, CD_3_OD) data; HRESIMS *m/z* 223.1327 (M + H)^+^ (calcd. for C_13_H_19_O_3_, 223.1334), molecular formula was C_13_H_18_O_3_.

### Electronic Circular Dichroism Calculations

The conformational analyses were performed for the enantiomers of all plausible stereoisomers of **4–6** using the SYBYL-X-2.1.1 program with the MMFF94s molecular force field. Gaussian 09 software was applied to screen stable conformers with the energy of the optimized structures at the B3LYP/6–31G (d) level (Frisch et al., 2010). The ECD curves of the conformers were determined by the TDDFT method at the B3LYP/6–31+G(d) level with the CPCM model in a methanol solution. SpecDis 1.7.1 software with UV correction was used to weigh the overall ECD curves by Boltzmann distribution of each conformer (Bruhn et al., 2017). The calculated ECD curves of **4–6** were compared with the experimental results for the absolute configuration determination.

### Cell Viability Assay (MTS)

SHSY5Y, SW480, A549, ACHN, and HepG2 cell lines cultured in 96-well plates with different concentrations (3.125, 6.25, 12.5, and 25 μM) of compounds **1** and **3** and *cis*-diaminedichloroplatinum (CDDP) to detect the half maximal inhibitory concentration (IC_50_). Compared to other compounds and cell lines, compounds **1** and **3** displayed a stronger inhibition on cell viability in the SW480 cell line. Therefore, we choose SW480, a human colon cancer cell line, to explore further activity evaluation of compounds **1** and **3**. SW480 cells cultured in 96-well plates (8,000 cells per well) with 100 µl complete culture media were carried out. After overnight incubation, cells were replaced with phenol red free complete medium, which was added with either drug-free or compound **1** or compound **3** (3.125, 6.25, 12.5, and 25 μM), or Z-V-FMK (20 μM), Z-Y-FMK (20 μM), and Nec-1 (30 μM) in different time points. Cells were cultured for the indicated period, and cell viability was detected at 492 nm by CellTiter 96 aqueous non-radioactive cell proliferation assay (Promega).

### Colony Growth Assay

SW480 cells were seeded at a concentration of 300 cells/ml in 6-well plates and cultured for 14 days to allow colony growth in the presence or absence of the indicated concentration of compound **1** or **3** (20 μM). Pictures were taken after 4% paraformaldehyde fixation and trypan blue stain and then the numbers of colony were calculated by ImageJ.

### Flow-Cytometry Assay

SW480 cells were treated with compound **1** or **3** (20 μM), then trypsinized and harvested (keeping all floating cells), washed with cold PBS buffer, followed by incubation with fluorescein isothiocyanate-labeled annexin V (FITC) and propidium iodide (PI) as instructed in the Annexin-V-FITC Apoptosis Detection Kit (Biovision Inc., Milpitas, CA, United States, K101-100) and analyzed by flow cytometry (FACSAria, Becton Dickinson, Franklin Lakes, NJ, United States). While PI-positive staining was necrotic, the cells with annexin V-positive and PI-negative stainings were calculated as apoptotic.

### Immunoblotting Analysis

SW480 cells were incubated overnight to reach about 70%–80% confluence before addition of compounds **1** and **3** in different time points. Whole cell lysate was obtained with lysis by using Triton X-100/glycerol buffer, and then the SDS-PAGE gel separation of the lysates was performed by utilizing 12% gel depending on the molecular weights of the desired proteins and transferred to PVDF (polyvinylidene fluoride) membrane. The membrane will be incubated in milk at room temperature for 1 h. Western blot was performed by using appropriate primary antibodies and horseradish peroxidase-conjugated suitable secondary antibodies, followed by detection with enhanced chemiluminescence (Pierce Chemical Rockford, IL, United States).

### Statistical Analysis

The images were analyzed to validate the linear range of chemiluminescence signals and quantifications were carried out by utilizing densitometry. The normally distributed data are shown as mean ± SD and analyzed using one-way analysis of variance and the Student–Newman–Keuls post-hoc test. Data are shown as mean ± SD in graphs. *p*-value < 0.05 was considered to have significant differences.

## Data Availability

The original contributions presented in the study are included in the article/Supplementary Material, further inquiries can be directed to the corresponding authors.
