# Supplementary material for: Diterpenoids and C13 Nor-Isoprenoid Identified From the Leaves and Twigs of Croton yanhuii Activating Apoptosis and Pyroptosis
Source: Front Chem. 2022 Mar 28;10:861278. doi: 10.3389/fchem.2022.861278 (PMC8996330; doi:10.3389/fchem.2022.861278)

Supplementary Material

Diterpenoids and C_{13} Nor-Isoprenoid Identified From the Leaves and Twigs of *Croton yanhuii* Activating Apoptosis and Pyroptosis

Yue-qian Li[1,2,3†], Bo-lin Hou[2,4†], Mei-jie Wang[1], Ru-yue Wang[1], Xiao-han Chen[1], Xu Liu[1], Dong-qing Fei[1,5*], Zhan-xin Zhang[1*], and Er-wei Li[2,3*]

[1]School of Pharmacy and State Key Laboratory of Applied Organic Chemistry, Lanzhou University, Lanzhou, China

[2]State Key Laboratory of Mycology, Institute of Microbiology, Chinese Academy of Sciences, Beijing, China

[3]Institutional Center for Shared Technologies and Facilities, Institute of Microbiology, Chinese Academy of Sciences, Beijing, China

[4]University of Chinese Academy of Sciences, Beijing, China

[5]State Key Laboratory for Chemistry and Molecular Engineering of Medicinal Resources,

*** Correspondence:**

Dong-Qing Fei

feidq@lzu.edu.cn

Zhan-Xin Zhang

zhangzhx@lzu.edu.cn

Er-Wei Li

liew@im.ac.cn

[†]These authors have contributed equally to this work and share first authorship

Contents of Supplementary Material

Figure S1. ^{1}H NMR spectrum of **1** in $CDCl_3$ (500 MHz) S4
Figure S2. ^{13}C NMR spectrum of **1** in $CDCl_3$ (125 MHz) S5
Figure S3. HSQC spectrum of **1** in $CDCl_3$ S6
Figure S4. HMBC spectrum of **1** in $CDCl_3$ S7
Figure S5. ^{1}H-^{1}H COSY spectrum of **1** in $CDCl_3$ S8
Figure S6. ROESY spectrum of **1** in $CDCl_3$ S9
Figure S7. HRESIMS spectrum of **1** S10
Figure S8. IR spectrum of **1** S11
Figure S9. ^{1}H NMR spectrum of **2** in $(CD_3)_2CO$ (500 MHz) S12
Figure S10. ^{13}C NMR spectrum of **2** in $(CD_3)_2CO$ (125 MHz) S13
Figure S11. HSQC spectrum of **2** in $(CD_3)_2CO$ S14
Figure S12. HMBC spectrum of **2** in $(CD_3)_2CO$ S15
Figure S13. ^{1}H-^{1}H COSY spectrum of **2** in $(CD_3)_2CO$ S16
Figure S14. ROESY spectrum of **2** in $(CD_3)_2CO$ S17
Figure S15. HRESIMS spectrum of **2** S18
Figure S16. IR spectrum of **2** S19
Figure S17. ^{1}H NMR spectrum of **3** in $(CD_3)_2CO$ (500 MHz) S20
Figure S18. ^{13}C NMR spectrum of **3** in $(CD_3)_2CO$ (125 MHz) S21
Figure S19. HSQC spectrum of **3** in $(CD_3)_2CO$ S22
Figure S20. HMBC spectrum of **3** in $(CD_3)_2CO$ S23
Figure S21. ^{1}H-^{1}H COSY spectrum of **3** in $(CD_3)_2CO$ S24
Figure S22. ROESY spectrum of **3** in $(CD_3)_2CO$ S25
Figure S23. HRESIMS spectrum of **3** S26
Figure S24. IR spectrum of **3** S27
Figure S25. ^{1}H NMR spectrum of **4** in $CDCl_3$ (500 MHz) S28
Figure S26. ^{13}C NMR and spectrum of **4** in $CDCl_3$ (125 MHz) S29
Figure S27. HSQC spectrum of **4** in $CDCl_3$ S30
Figure S28. HMBC spectrum of **4** in $CDCl_3$ S31
Figure S29. ^{1}H-^{1}H COSY spectrum of **4** in $CDCl_3$ S32
Figure S30. ROESY spectrum of **4** in $CDCl_3$ S33
Figure S31. HRESIMS spectrum of **4** S34
Figure S32. IR spectrum of **4** S35
Figure S33. ^{1}H NMR spectrum of **5** in $(CD_3)_2CO$ (500 MHz) S36
Figure S34. ^{13}C NMR spectrum of **5** in $(CD_3)_2CO$ (125 MHz) S37
Figure S35. HSQC spectrum of **5** in $(CD_3)_2CO$ S38
Figure S36. HMBC spectrum of **5** in $(CD_3)_2CO$ S39
Figure S37. ^{1}H-^{1}H COSY spectrum of **5** in $(CD_3)_2CO$ S40
Figure S38. ROESY spectrum of **5** in $(CD_3)_2CO$ S41
Figure S39. HRESIMS spectrum of **5** S42
Figure S40. IR spectrum of **5** S43
Figure S41. ^{1}H NMR spectrum of **6** in CD_3OD (500 MHz) S44

Figure S42. ^{13}C NMR spectrum of **6** in CD_3OD (125 MHz) S45
Figure S43. HSQC spectrum of **6** in CD_3OD S46
Figure S44. HMBC spectrum of **6** in CD_3OD S47
Figure S45. ^1H-^1H COSY spectrum of **6** in CD_3OD S48
Figure S46. ROESY spectrum of **6** in CD_3OD S49
Figure S47. HRESIMS spectrum of **6** S50
Figure S48. IR spectrum of **6** S51

Figure S1. ^{1}H NMR spectrum of **1** in $CDCl_3$ (500 MHz)

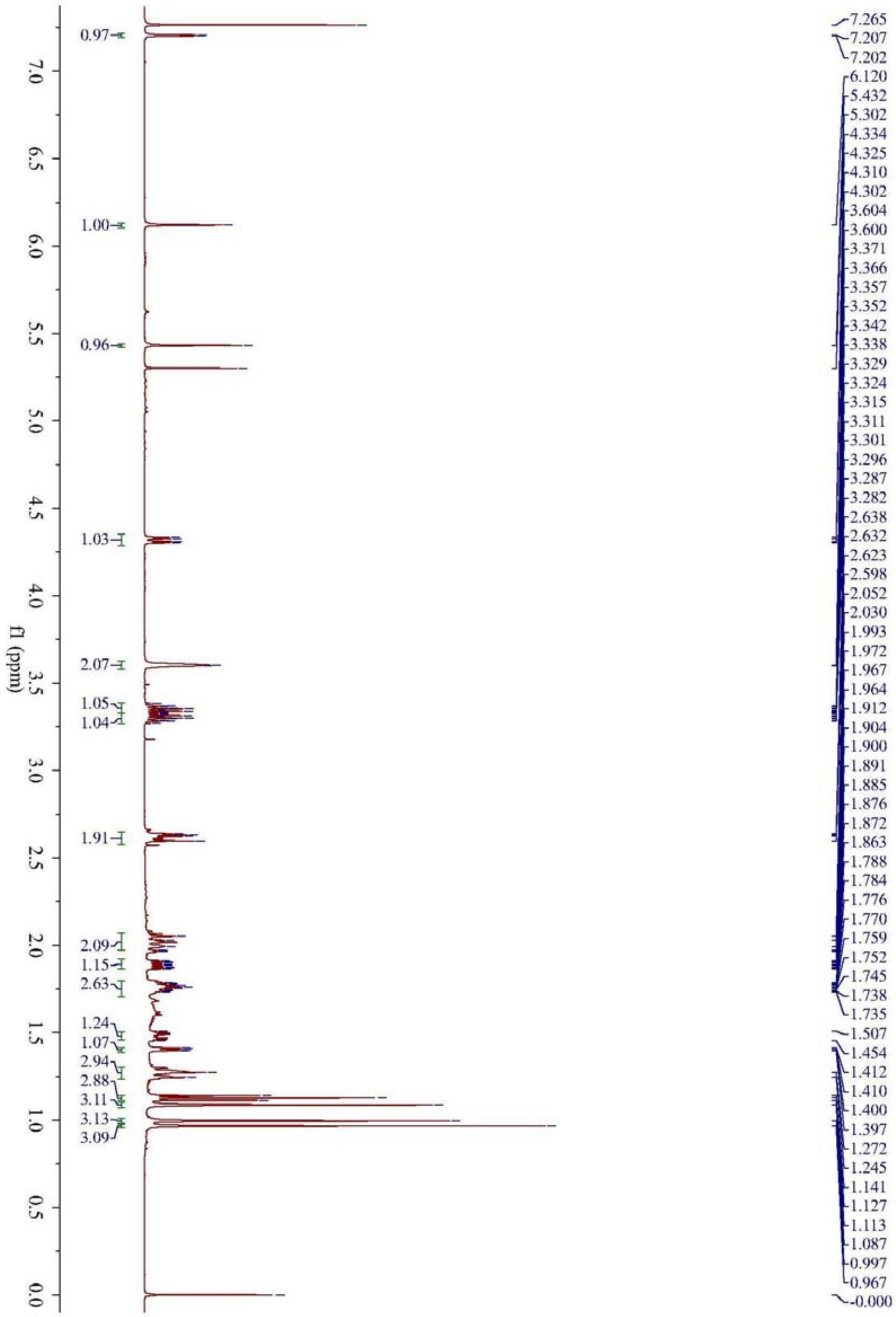

Figure S2. ^{13}C NMR spectrum of **1** in $CDCl_3$ (125 MHz)

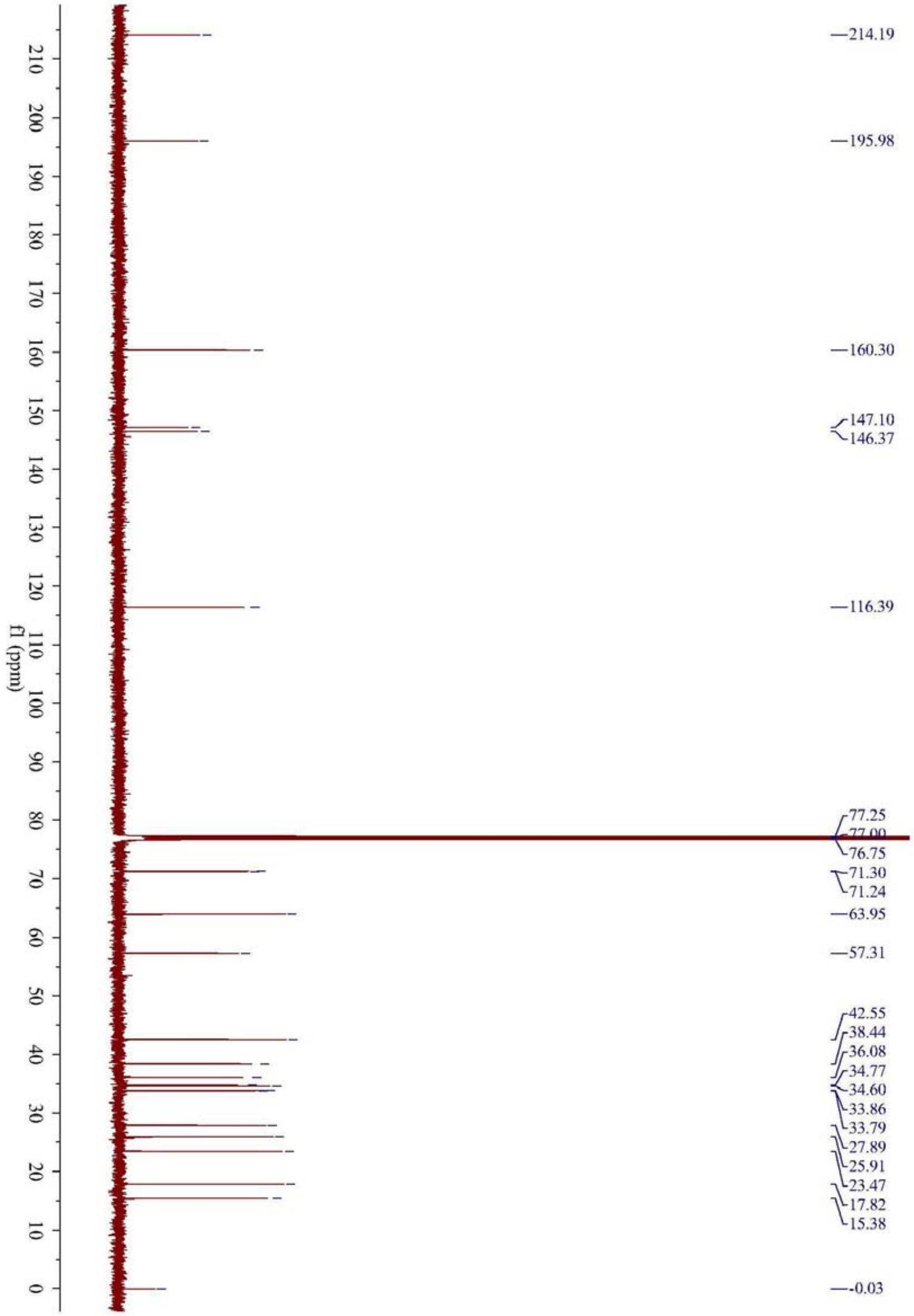

Figure S3. HSQC spectrum of **1** in $CDCl_3$

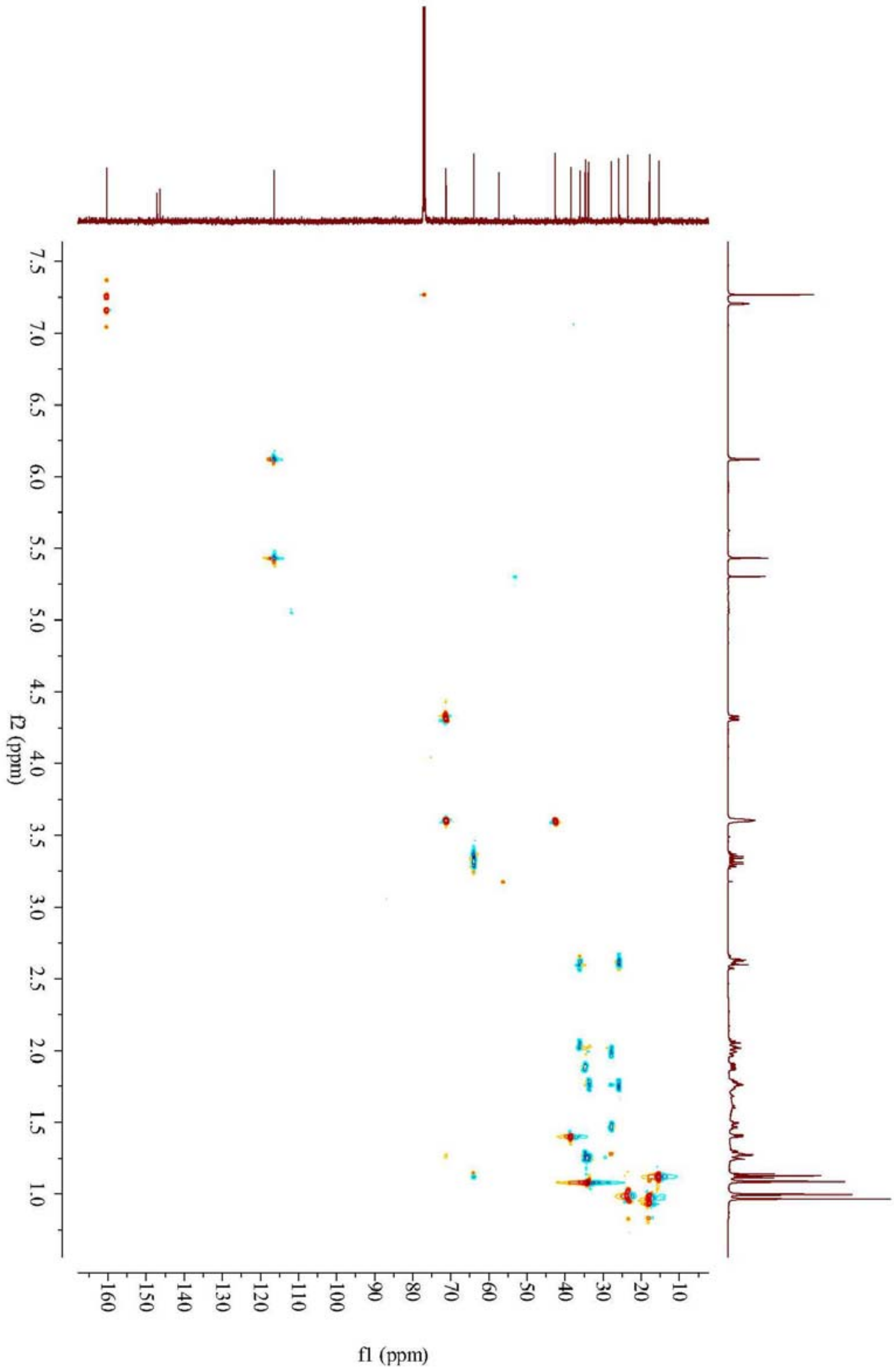

Figure S4. HMBC spectrum of **1** in $CDCl_3$

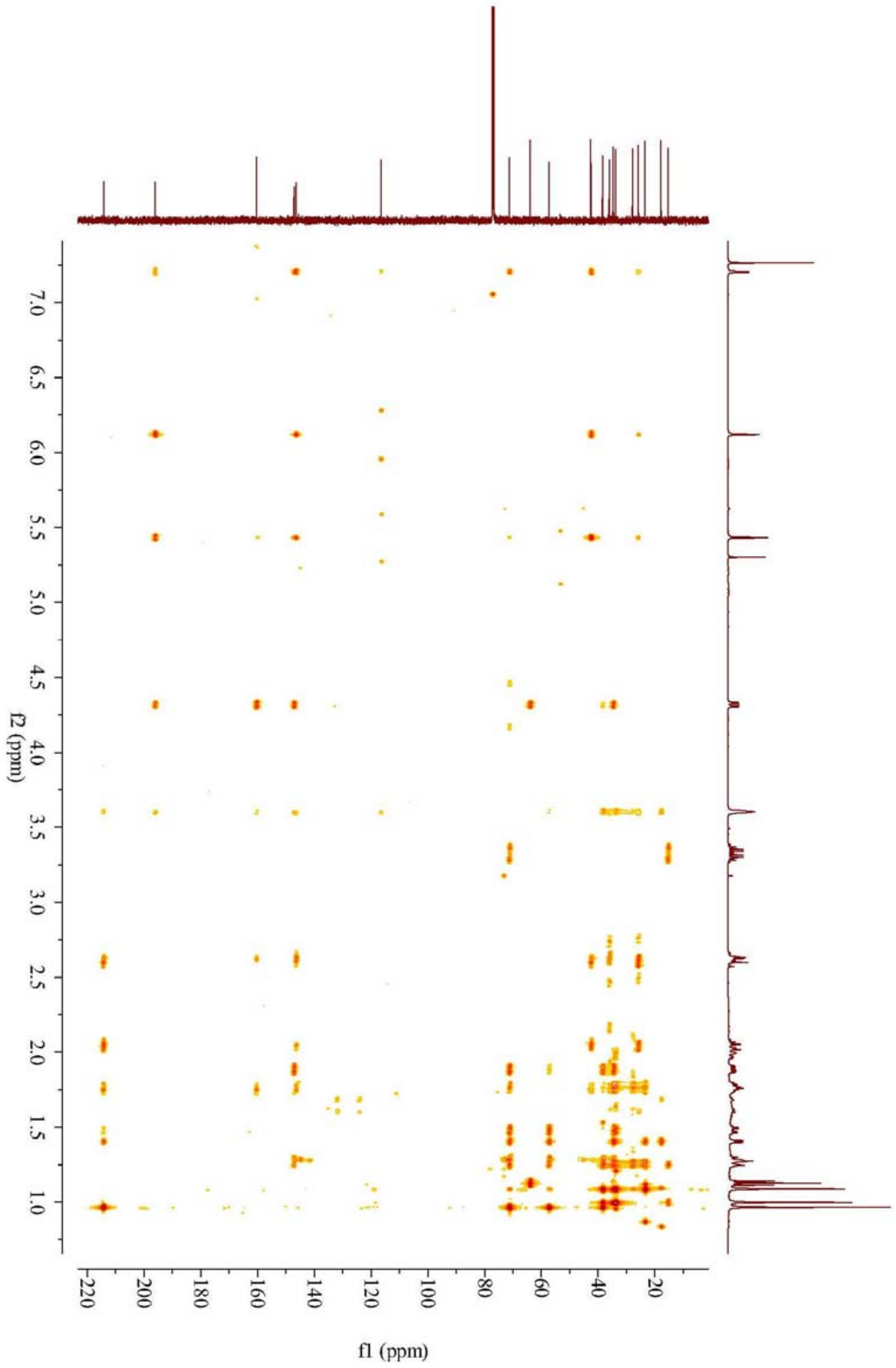

Figure S5. ^{1}H-^{1}H COSY spectrum of **1** in $CDCl_3$

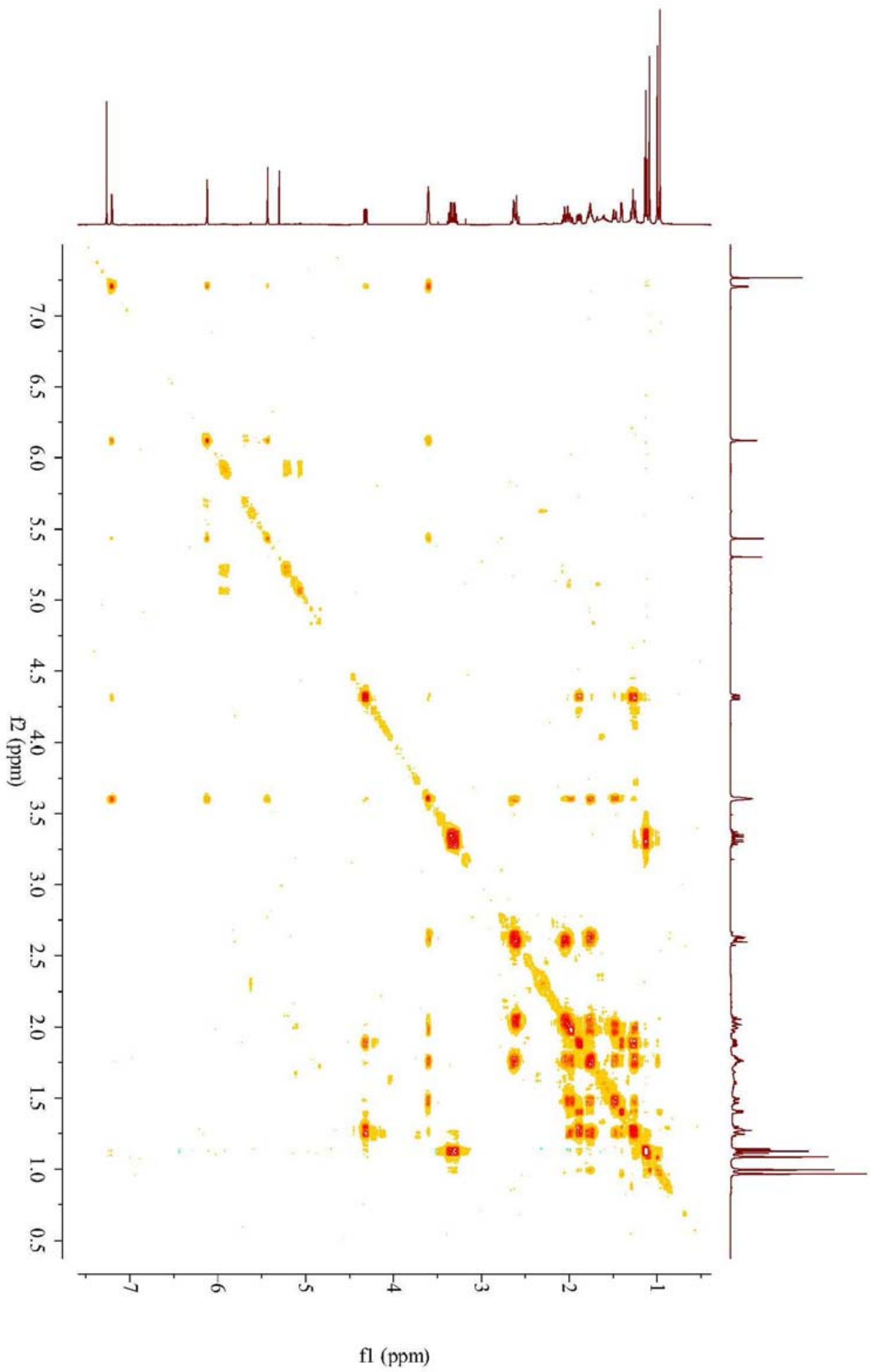

Figure S6. ROESY spectrum of **1** in $CDCl_3$

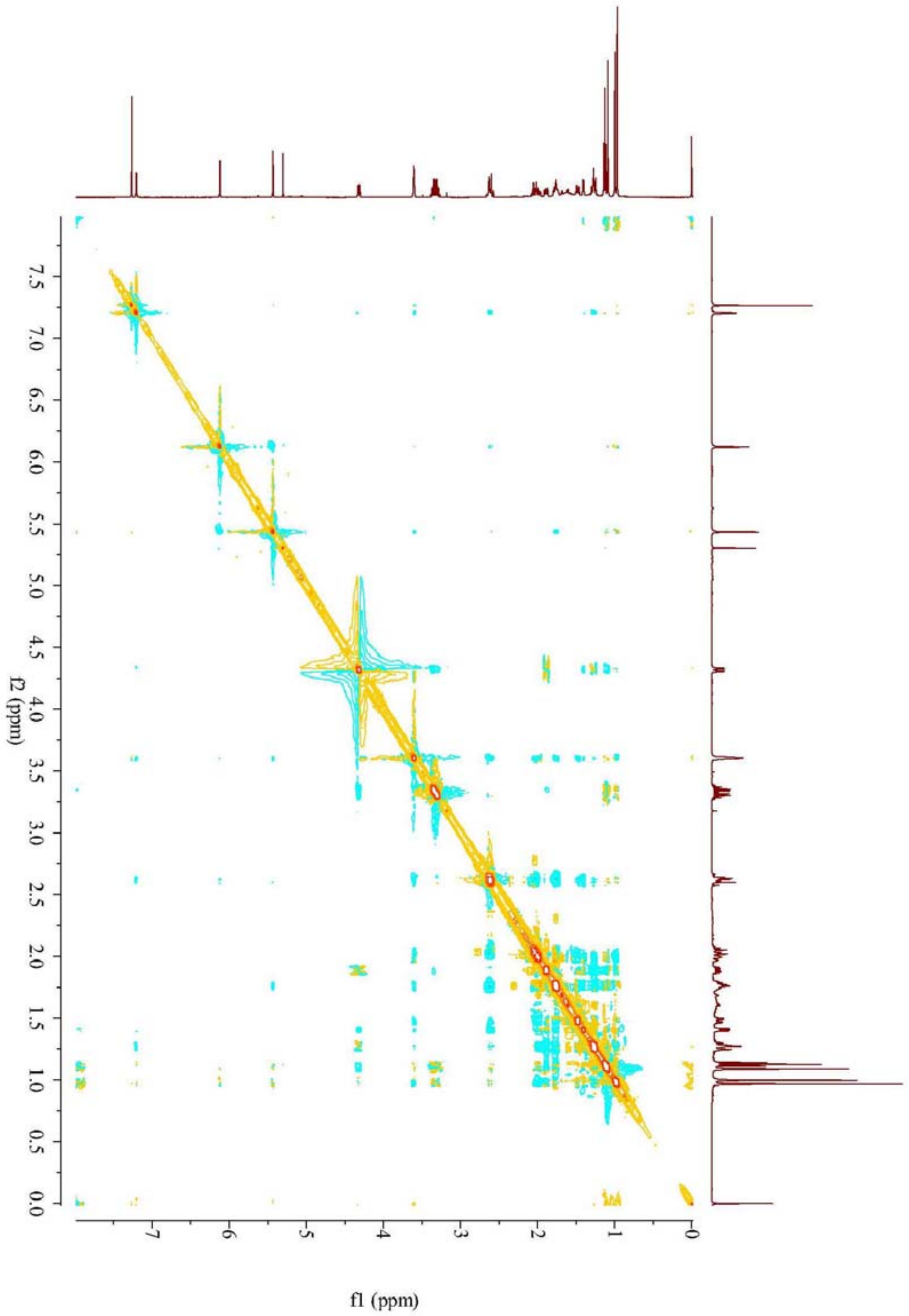

Figure S7. HRESIMS spectrum of **1**

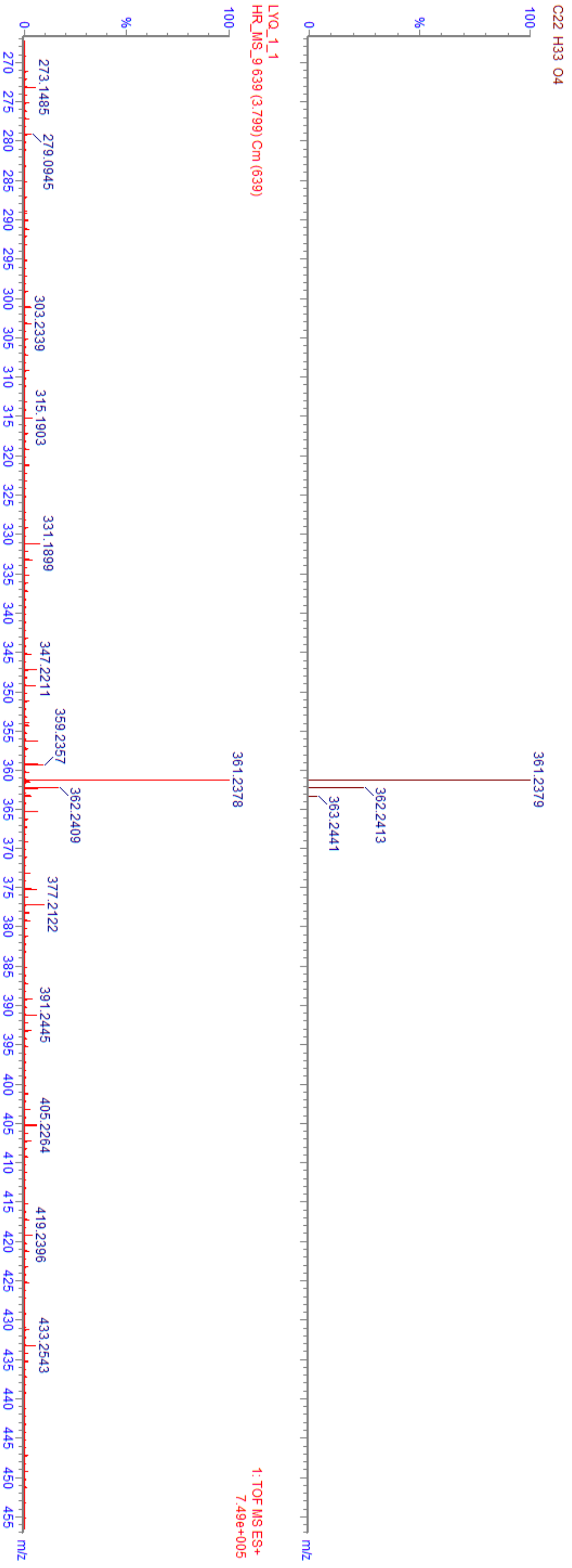

Figure S8. IR spectrum of **1**

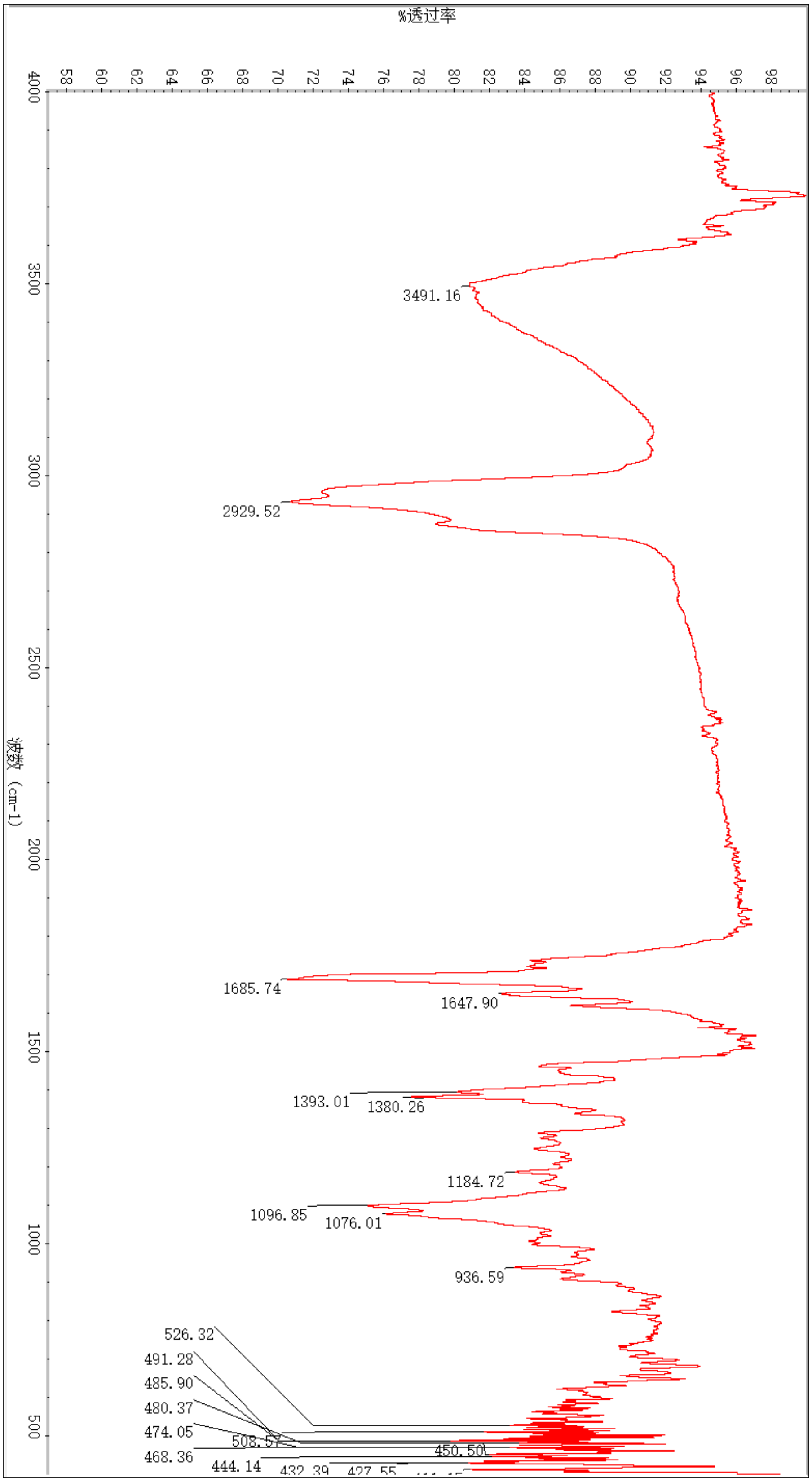

Figure S9. ^{1}H NMR spectrum of **2** in $(CD_3)_2CO$ (500 MHz)

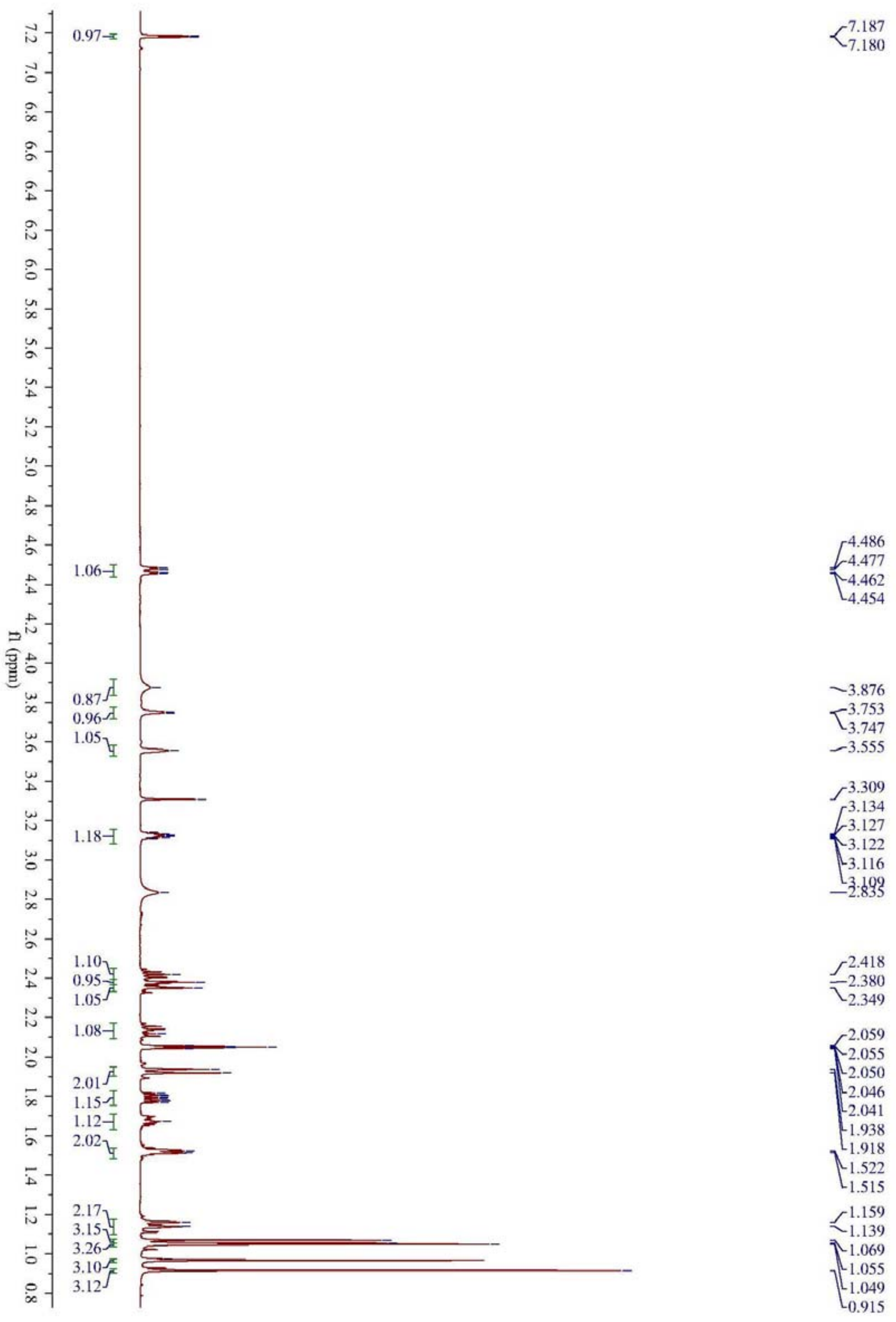

Figure S10. ^{13}C NMR spectrum of **2** in $(CD_3)_2CO$ (125 MHz)

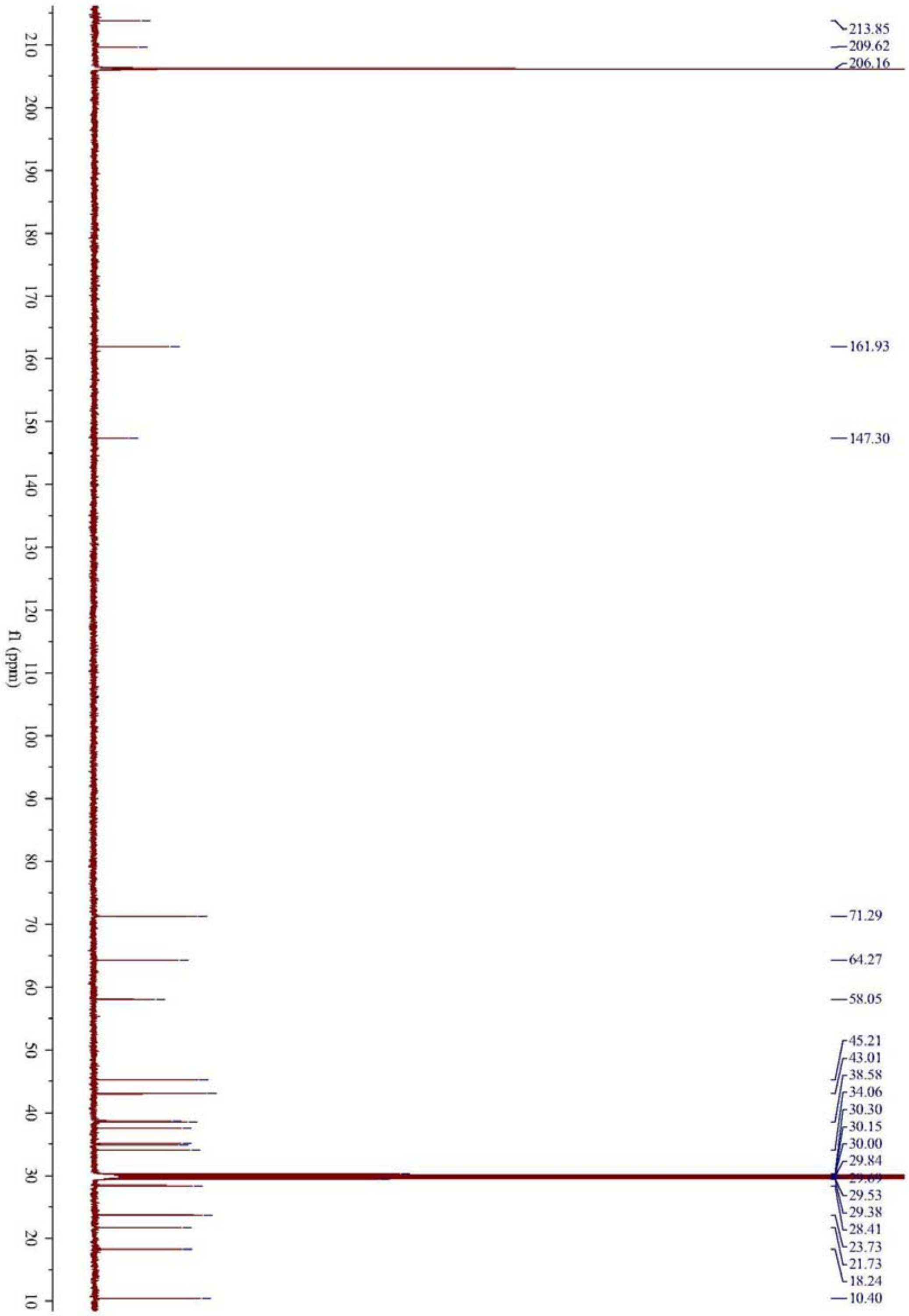

Figure S11. HSQC spectrum of **2** in $(CD_3)_2CO$

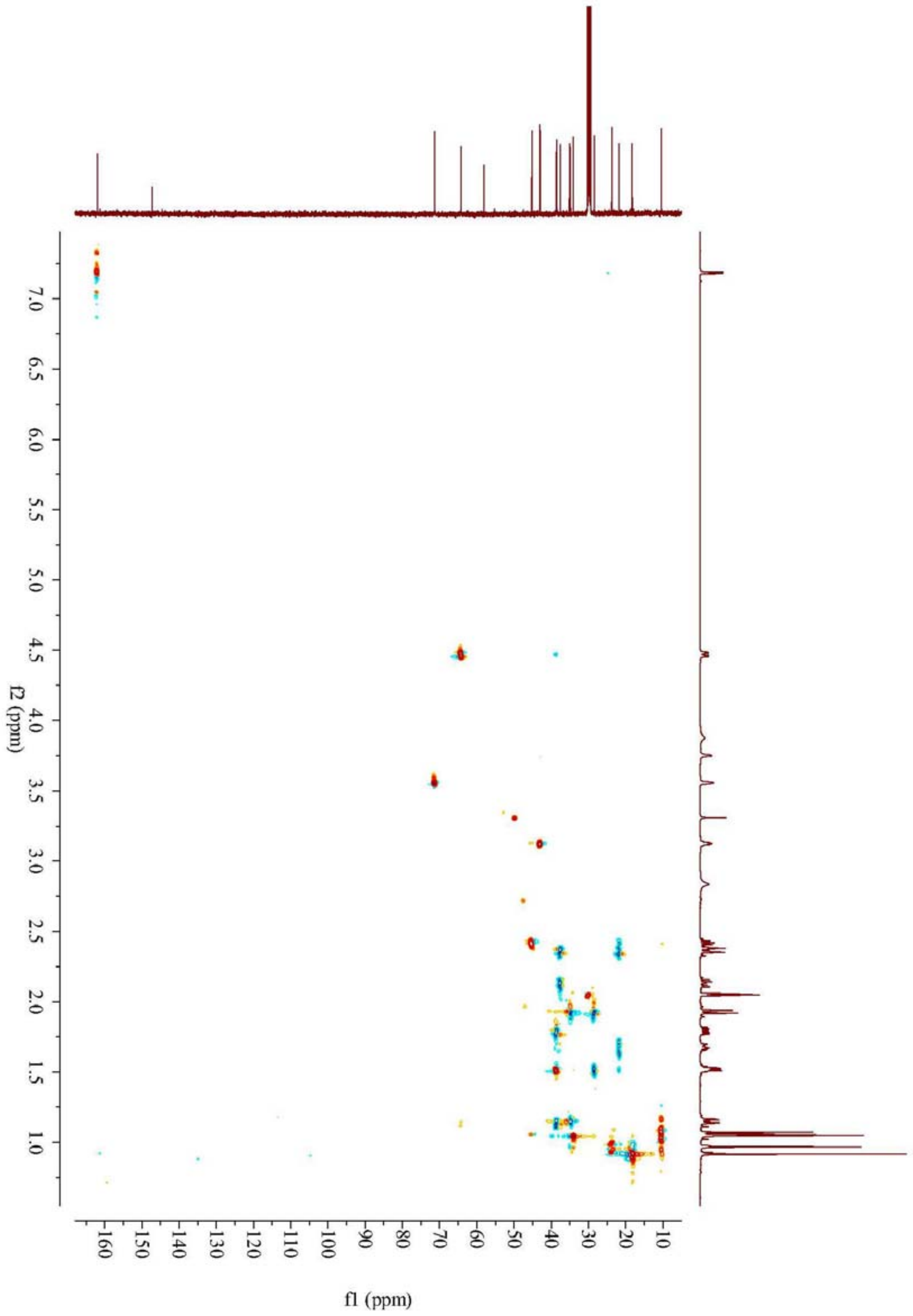

Figure S12. HMBC spectrum of **2** in $(CD_3)_2CO$

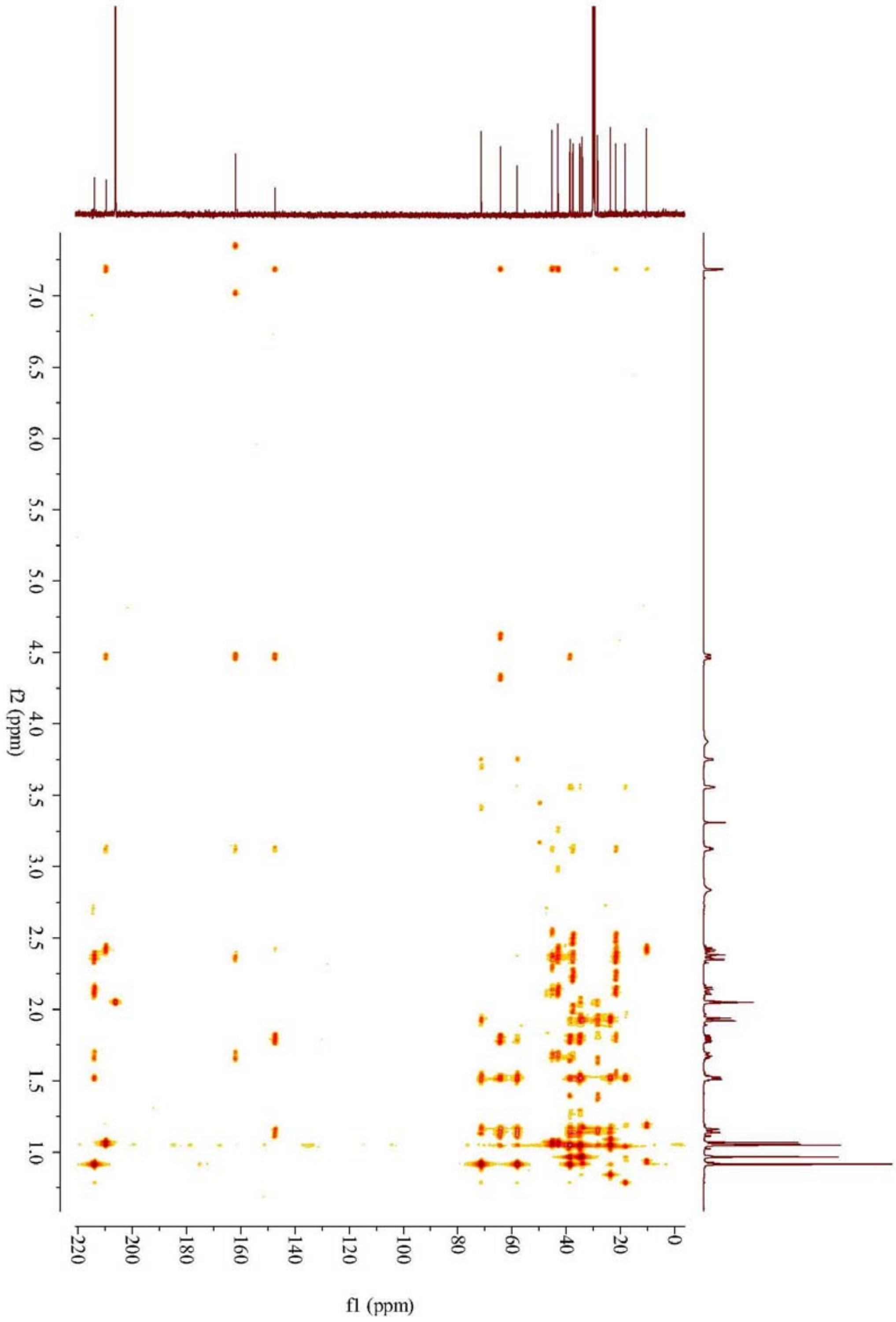

Figure S13. ^{1}H-^{1}H COSY spectrum of **2** in $(CD_3)_2CO$

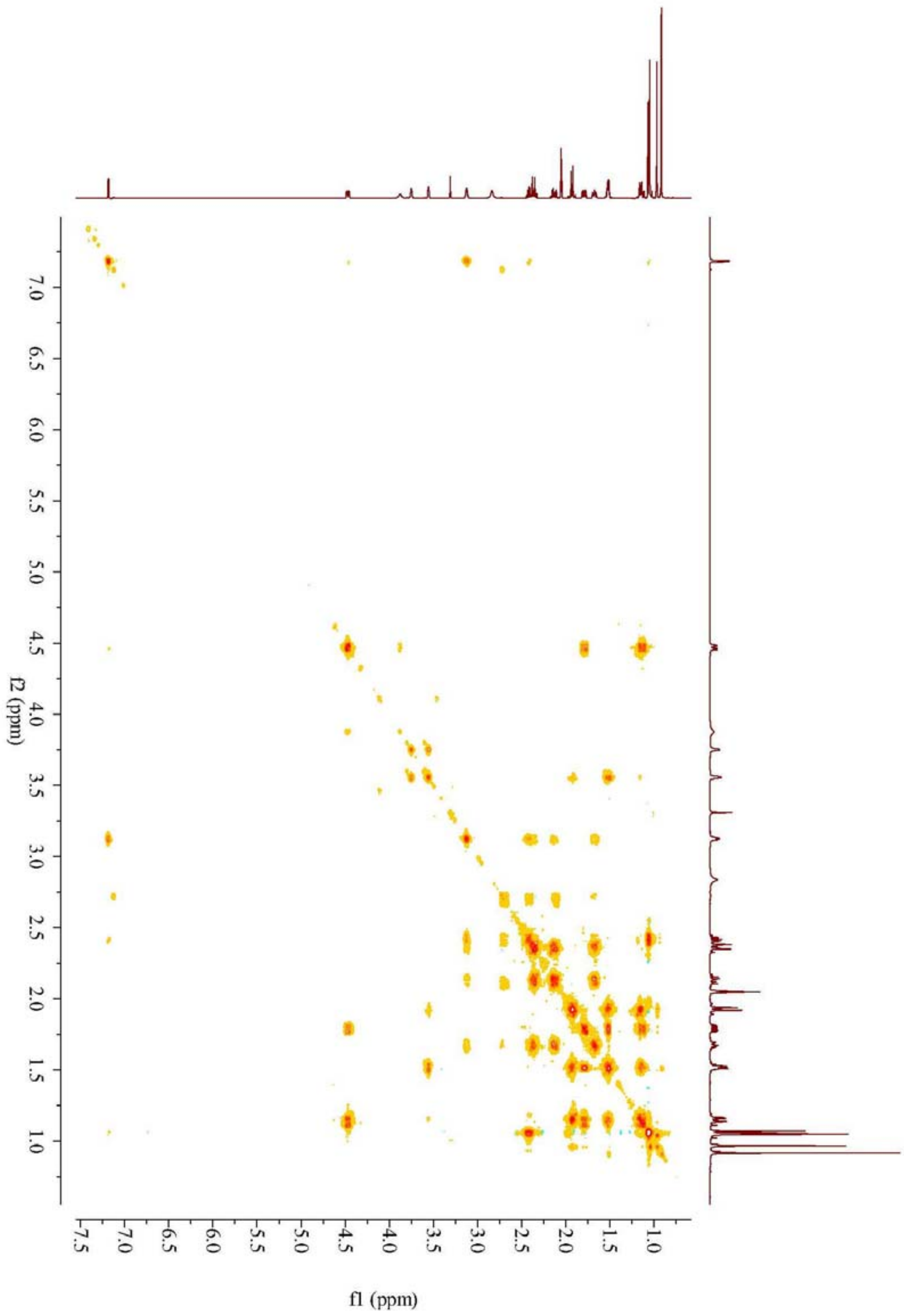

Figure S14. ROESY spectrum of **2** in $(CD_3)_2CO$

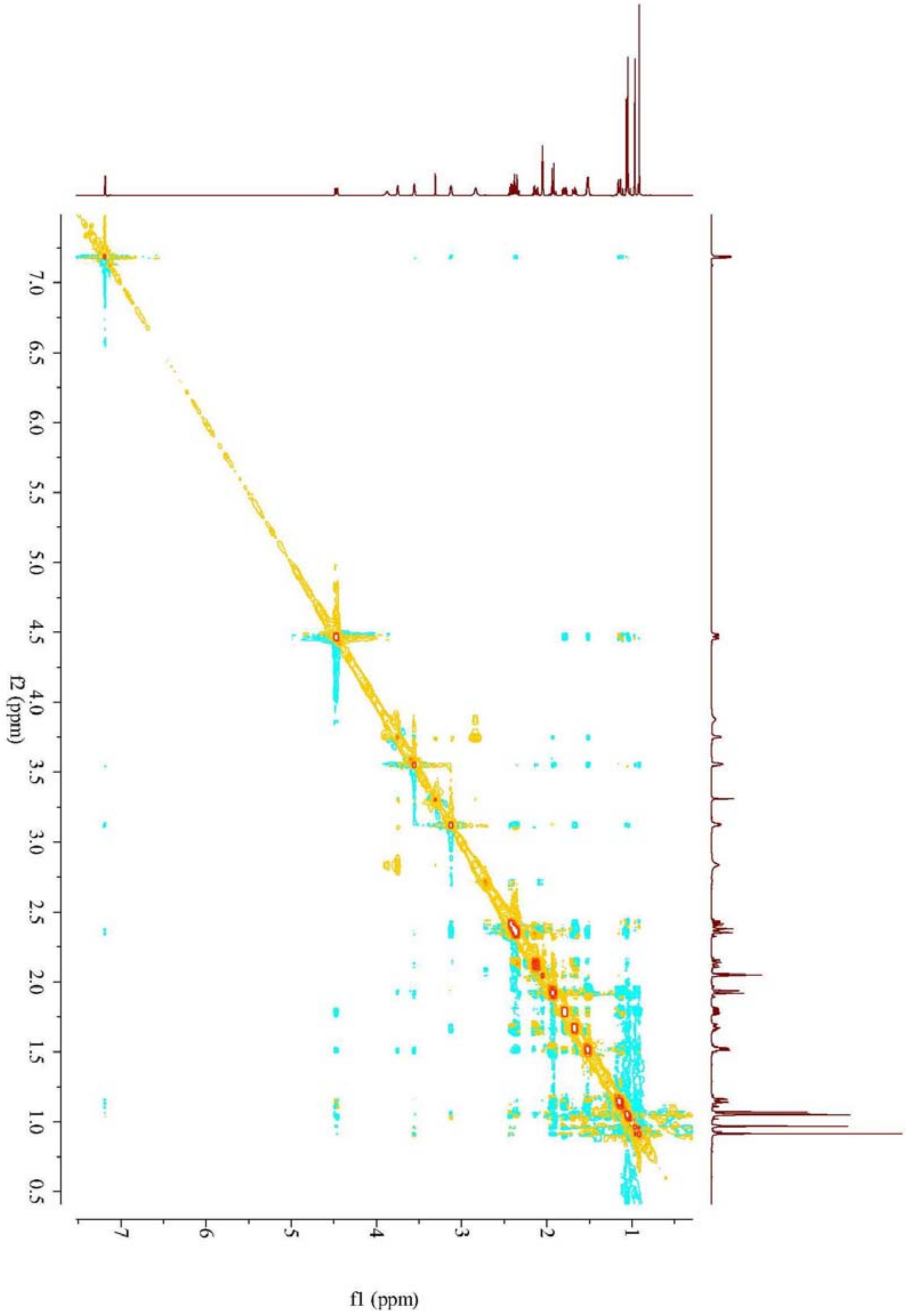

Figure S15. HRESIMS spectrum of **2**

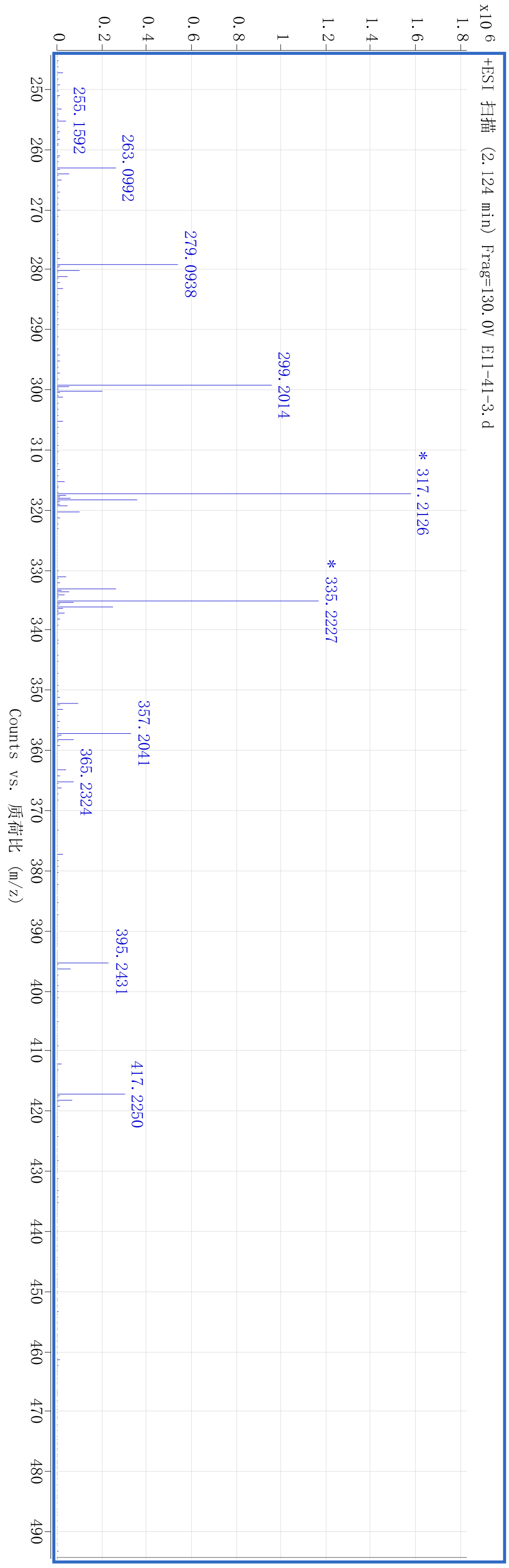

Figure S16. IR spectrum of **2**

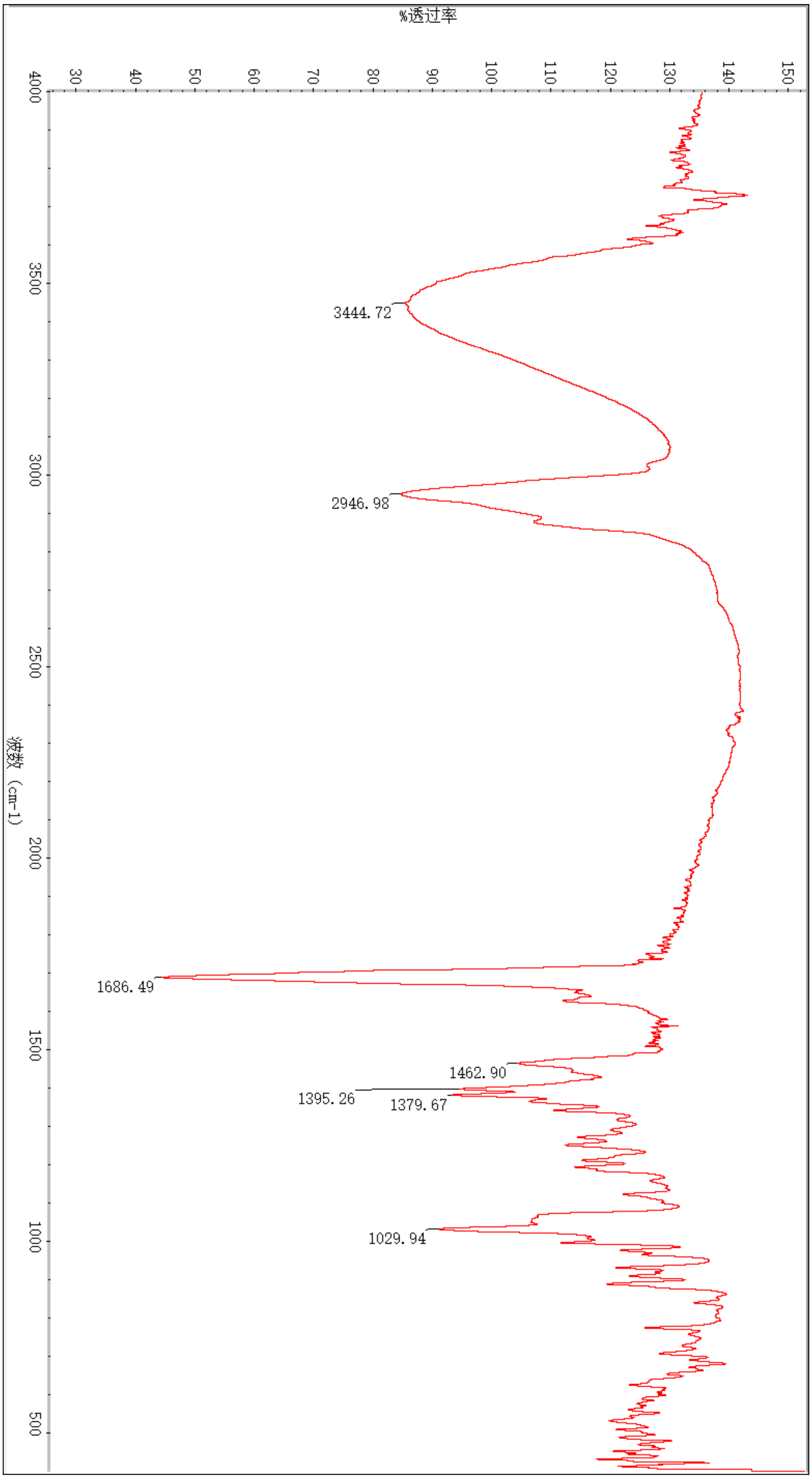

Figure S17. ^{1}H NMR spectrum of **3** in $(CD_3)_2CO$ (500 MHz)

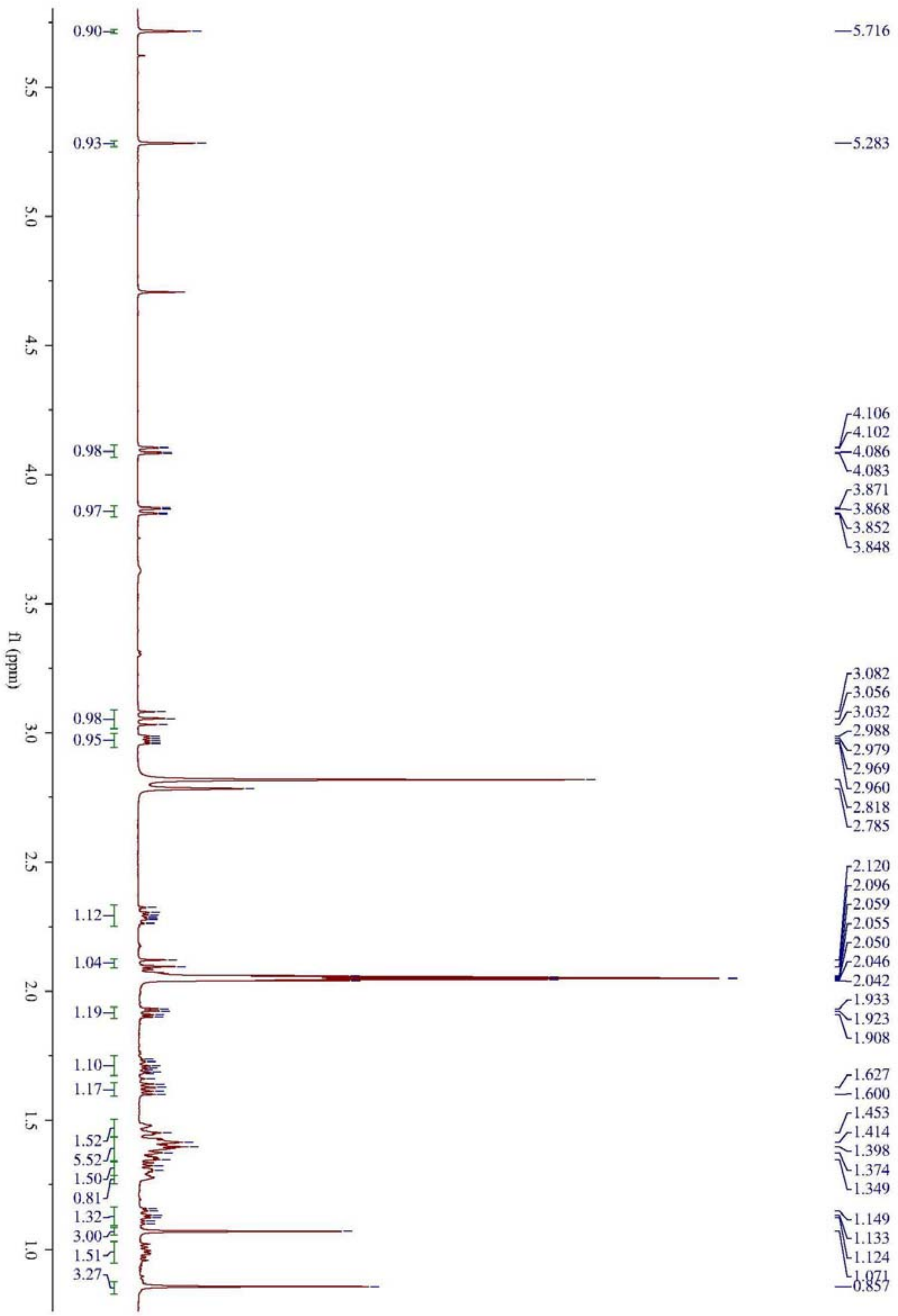

Figure S18. ^{13}C NMR spectrum of **3** in $(CD_3)_2CO$ (125 MHz)

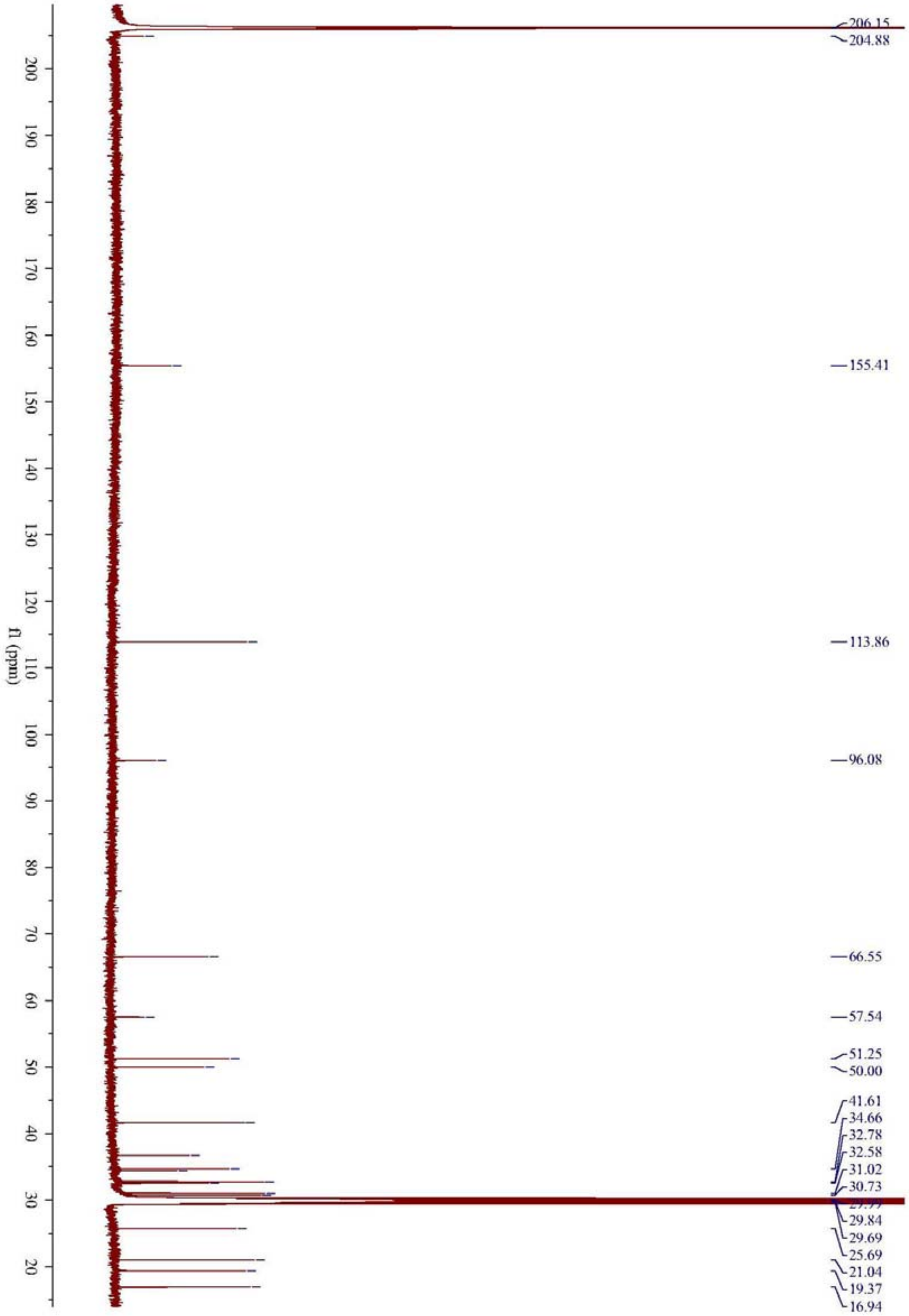

Figure S19. HSQC spectrum of **3** in $(CD_3)_2CO$

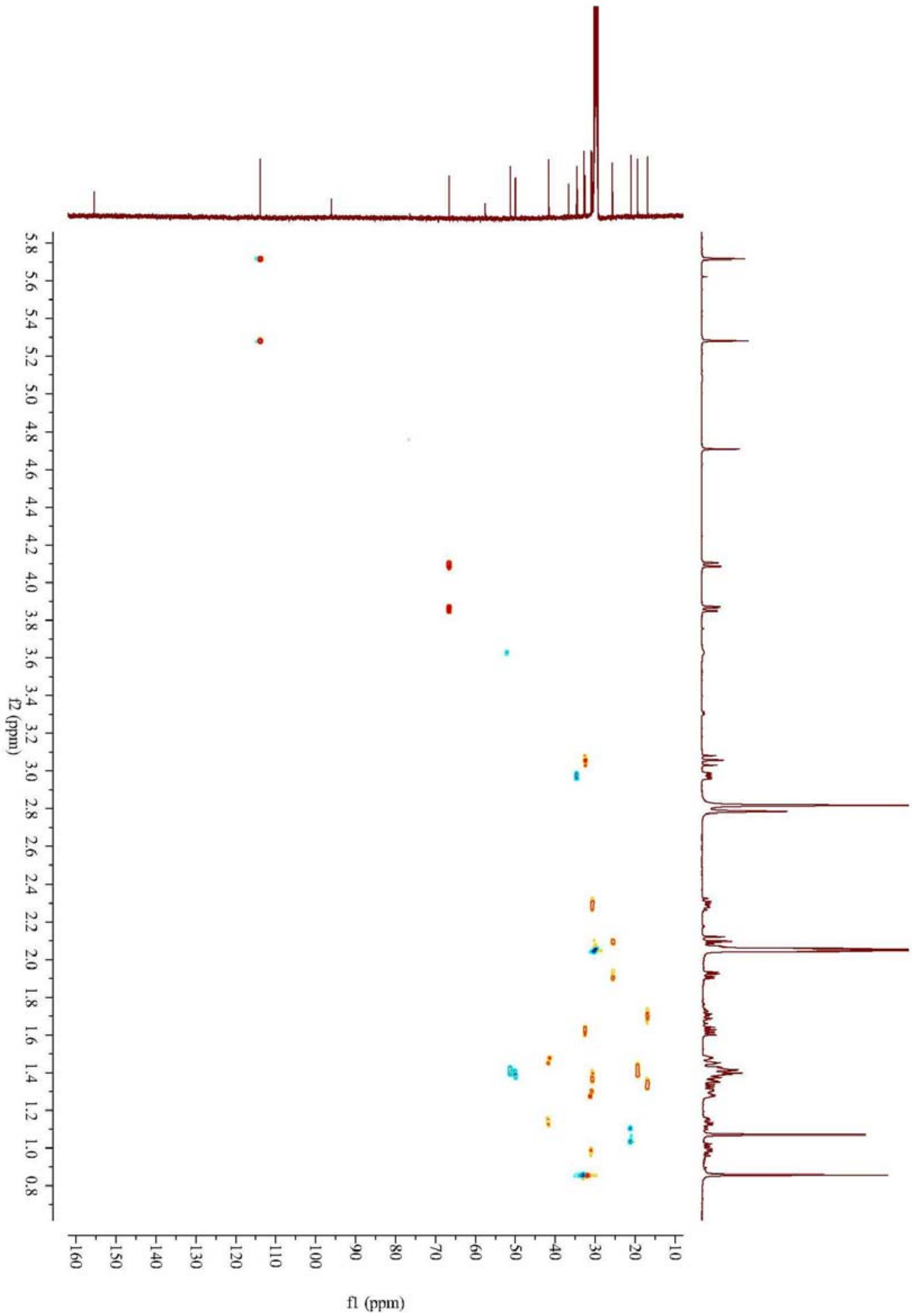

Figure S20. HMBC spectrum of **3** in $(CD_3)_2CO$

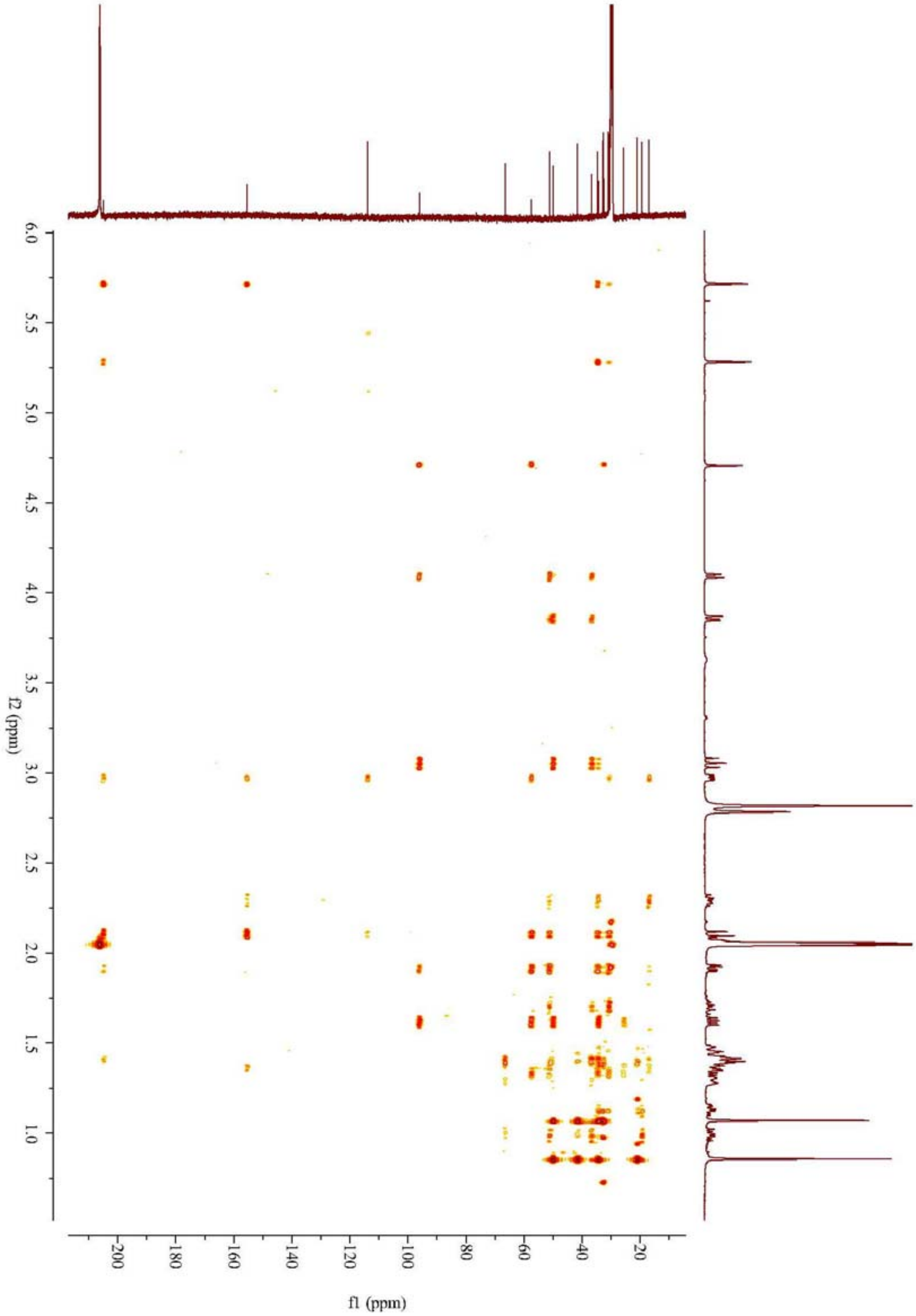

Figure S21. ^{1}H-^{1}H COSY spectrum of **3** in $(CD_3)_2CO$

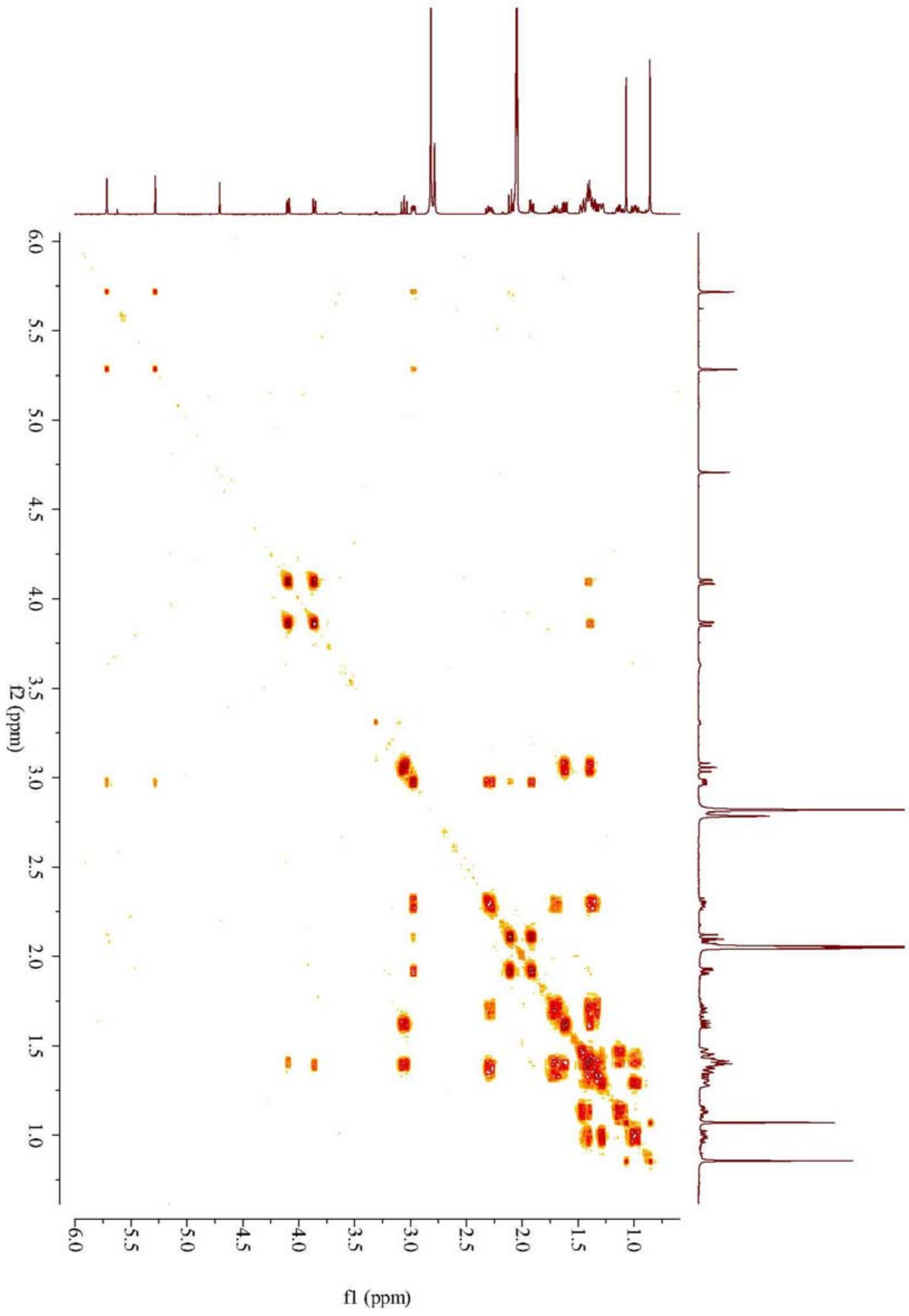

Figure S22. ROESY spectrum of **3** in $(CD_3)_2CO$

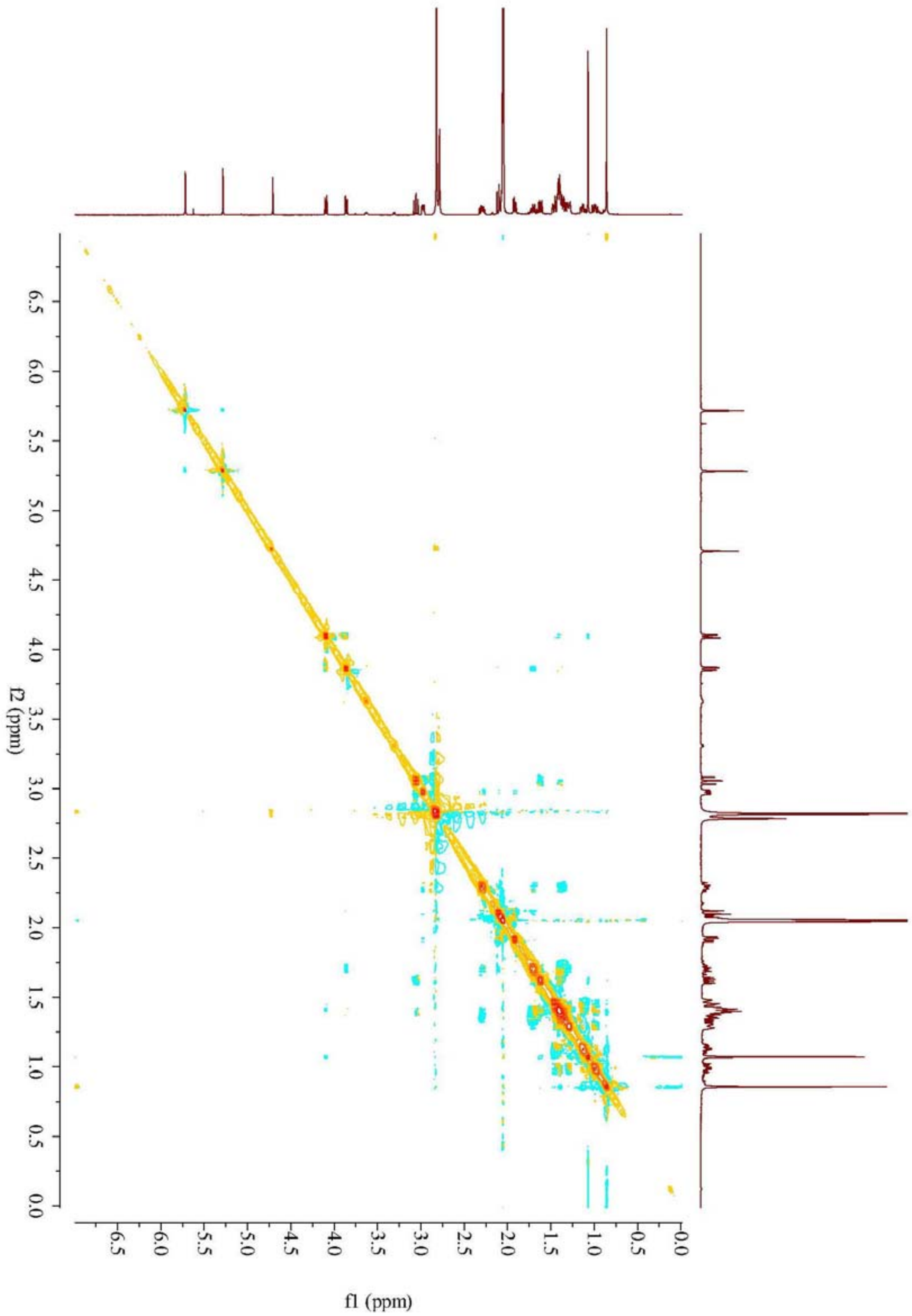

Figure S23. HRESIMS spectrum of **3**

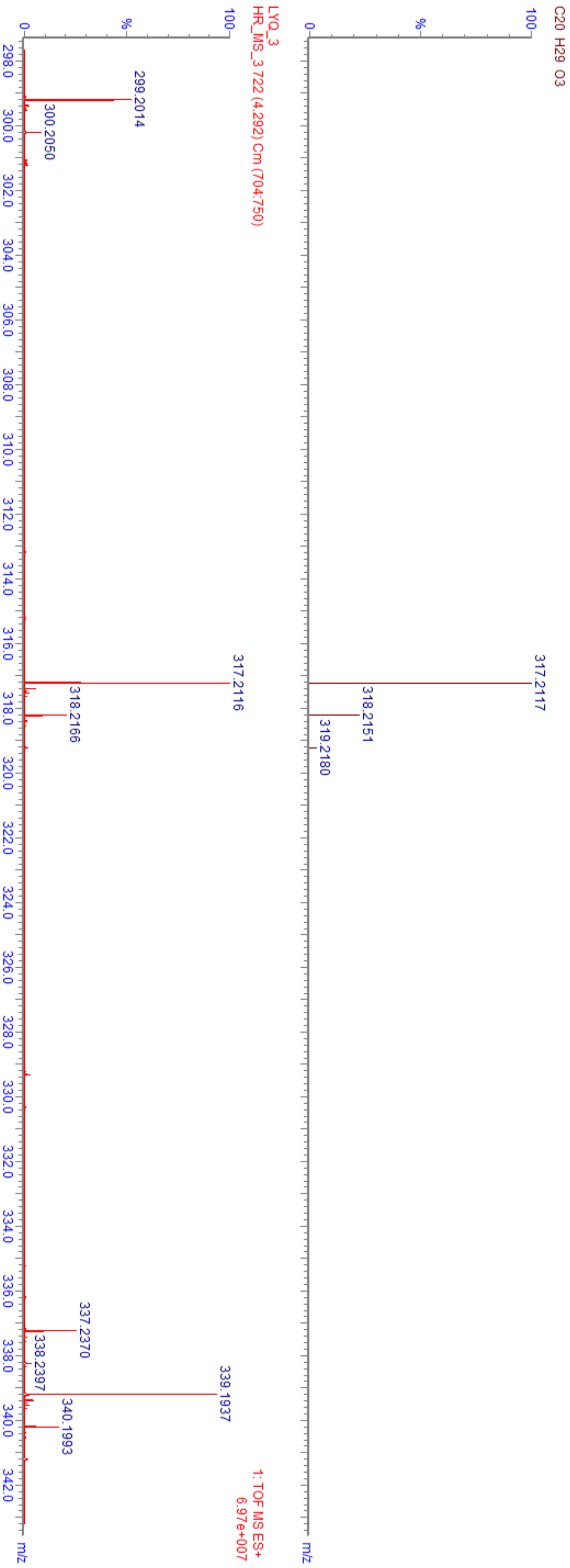

Figure S24. IR spectrum of **3**

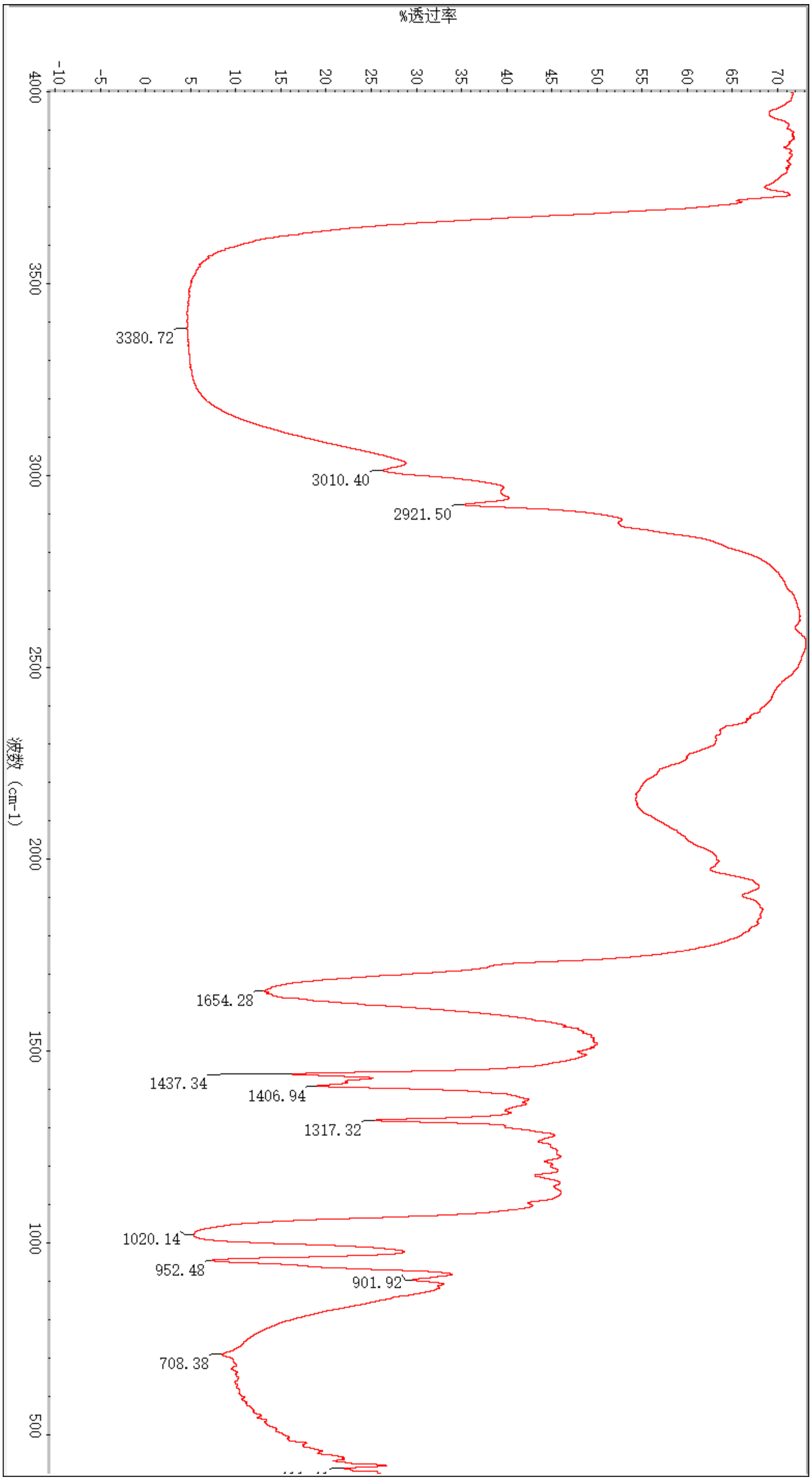

Figure S25. ^{1}H NMR spectrum of **4** in $CDCl_3$ (500 MHz)

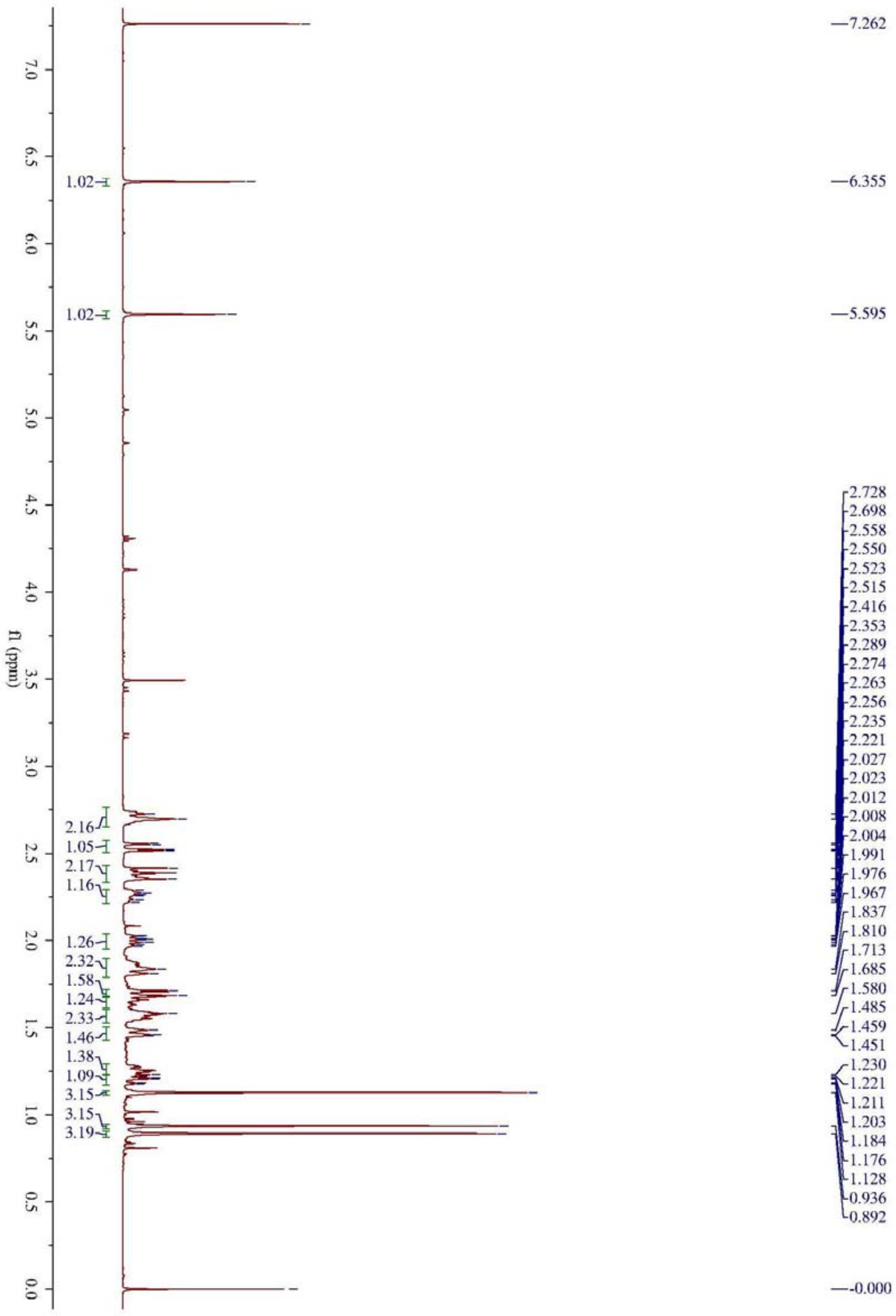

Figure S26. ^{13}C NMR and spectrum of **4** in $CDCl_3$ (125 MHz)

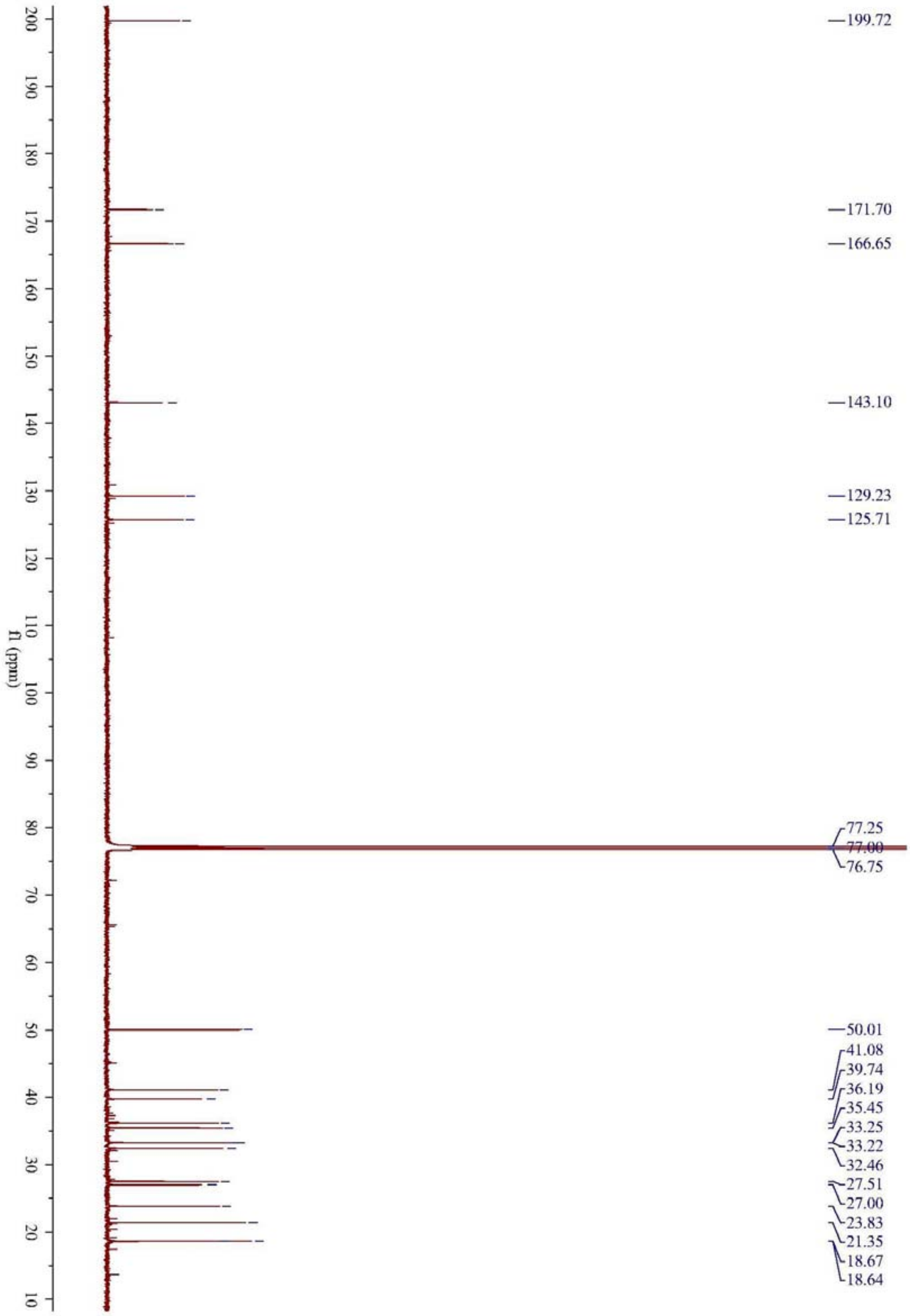

Figure S27. HSQC spectrum of **4** in $CDCl_3$

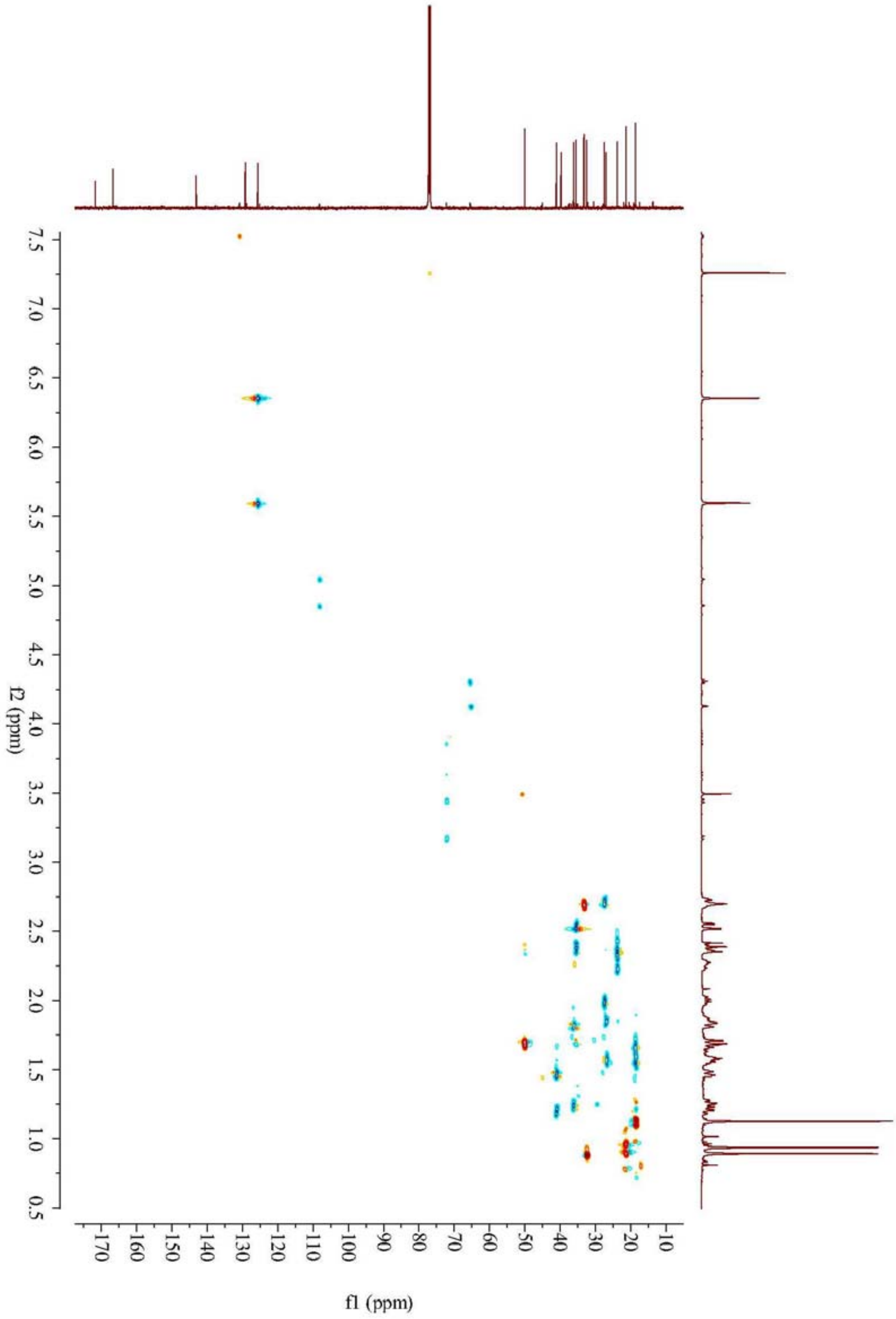

Figure S28. HMBC spectrum of **4** in $CDCl_3$

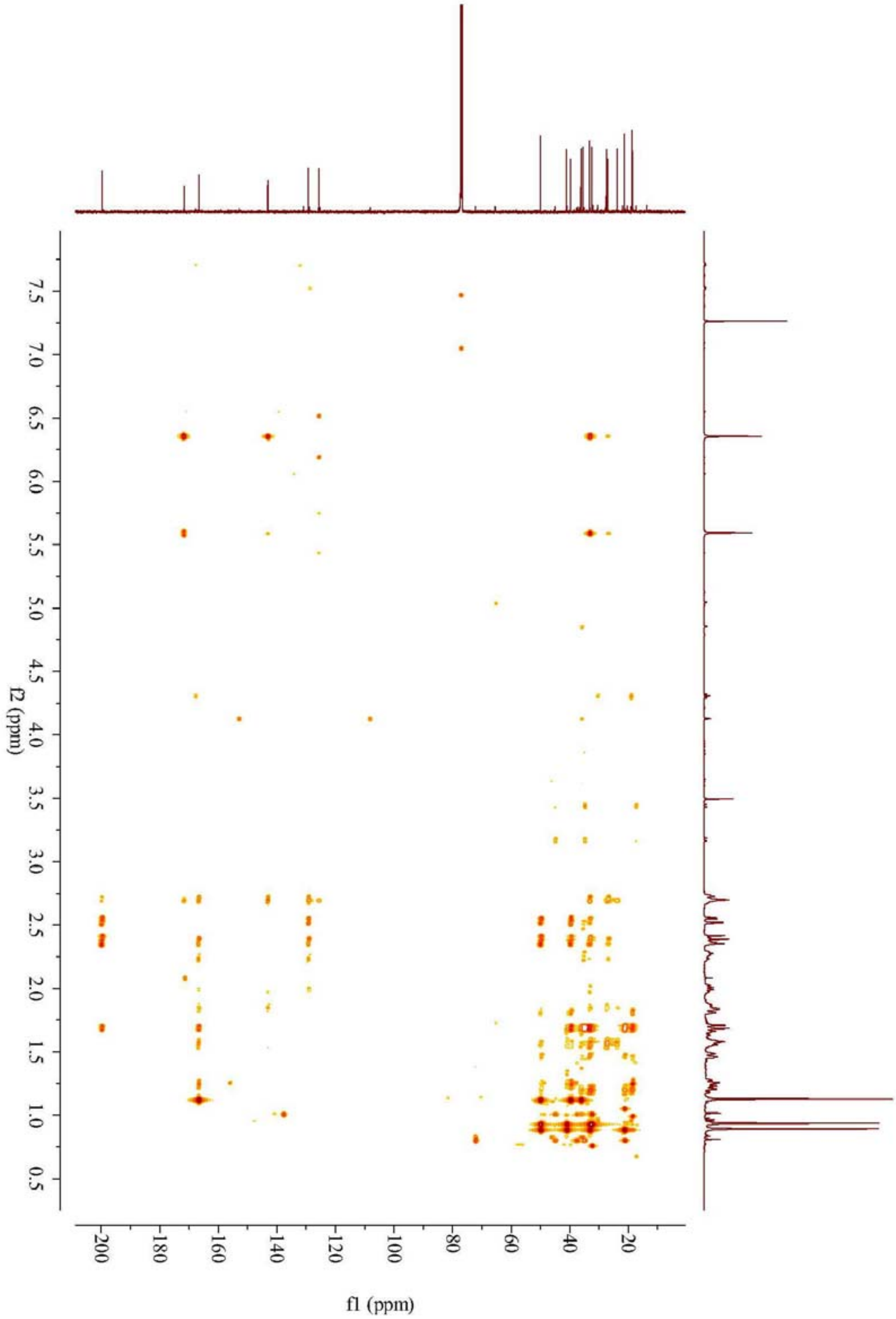

Figure S29. ^{1}H-^{1}H COSY spectrum of **4** in $CDCl_3$

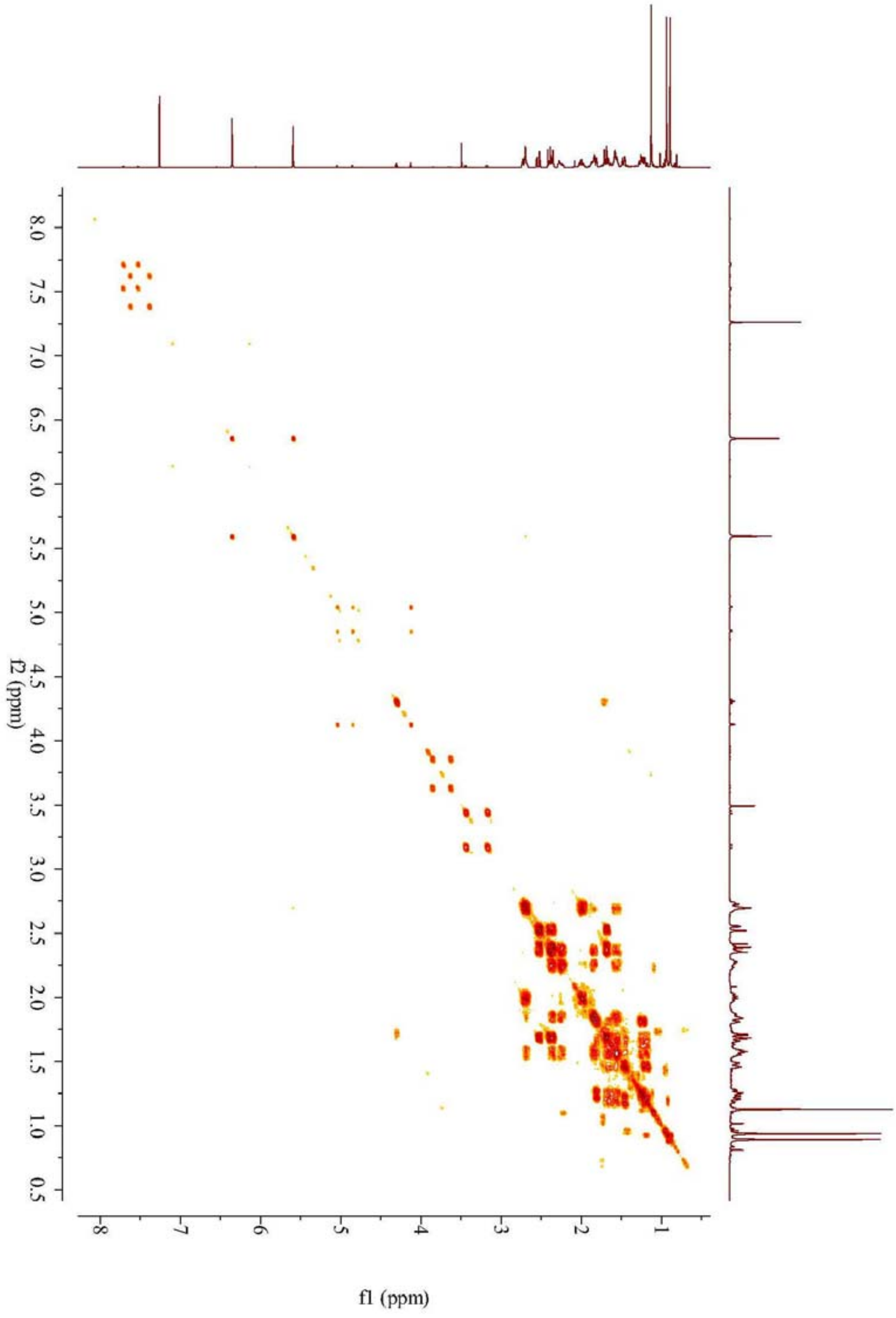

Figure S30. ROESY spectrum of **4** in $CDCl_3$

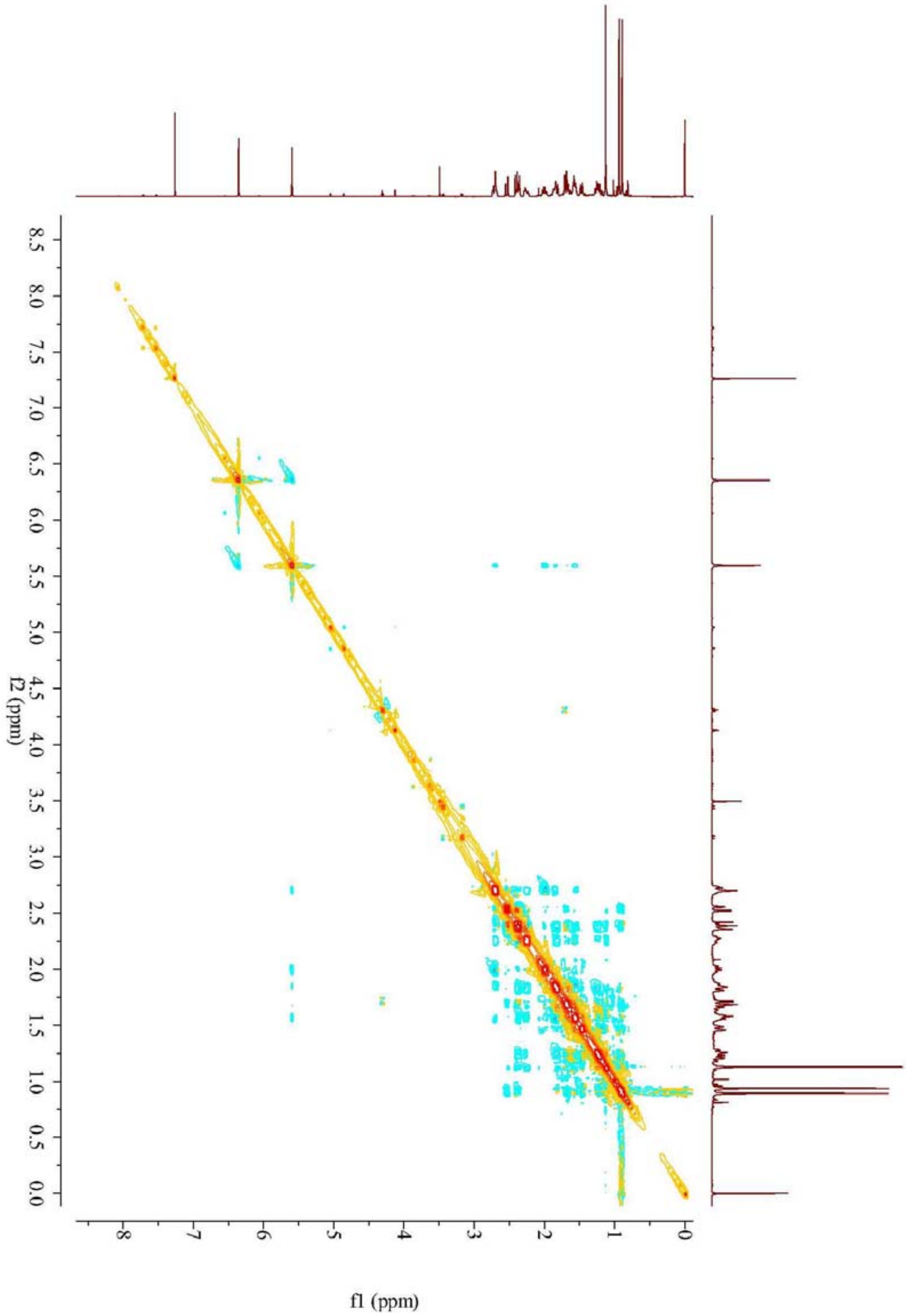

Figure S31. HRESIMS spectrum of **4**

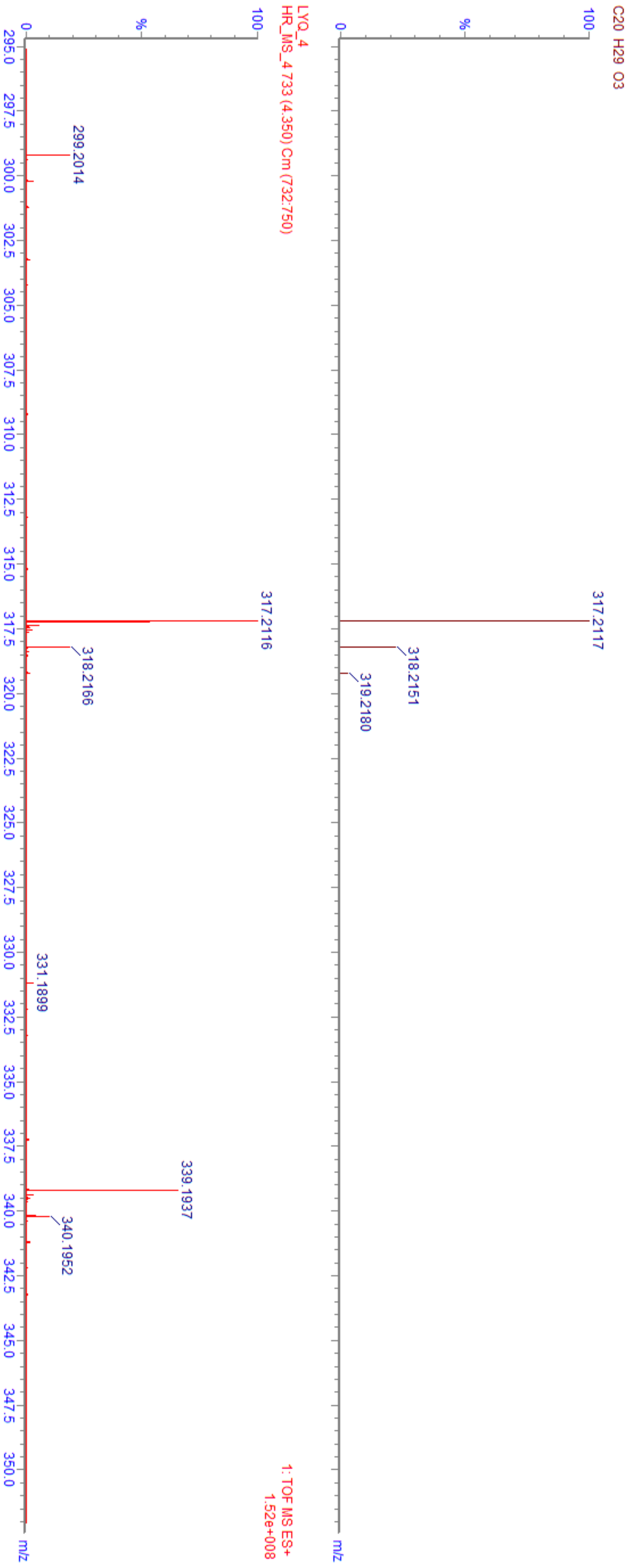

Figure S32. IR spectrum of **4**

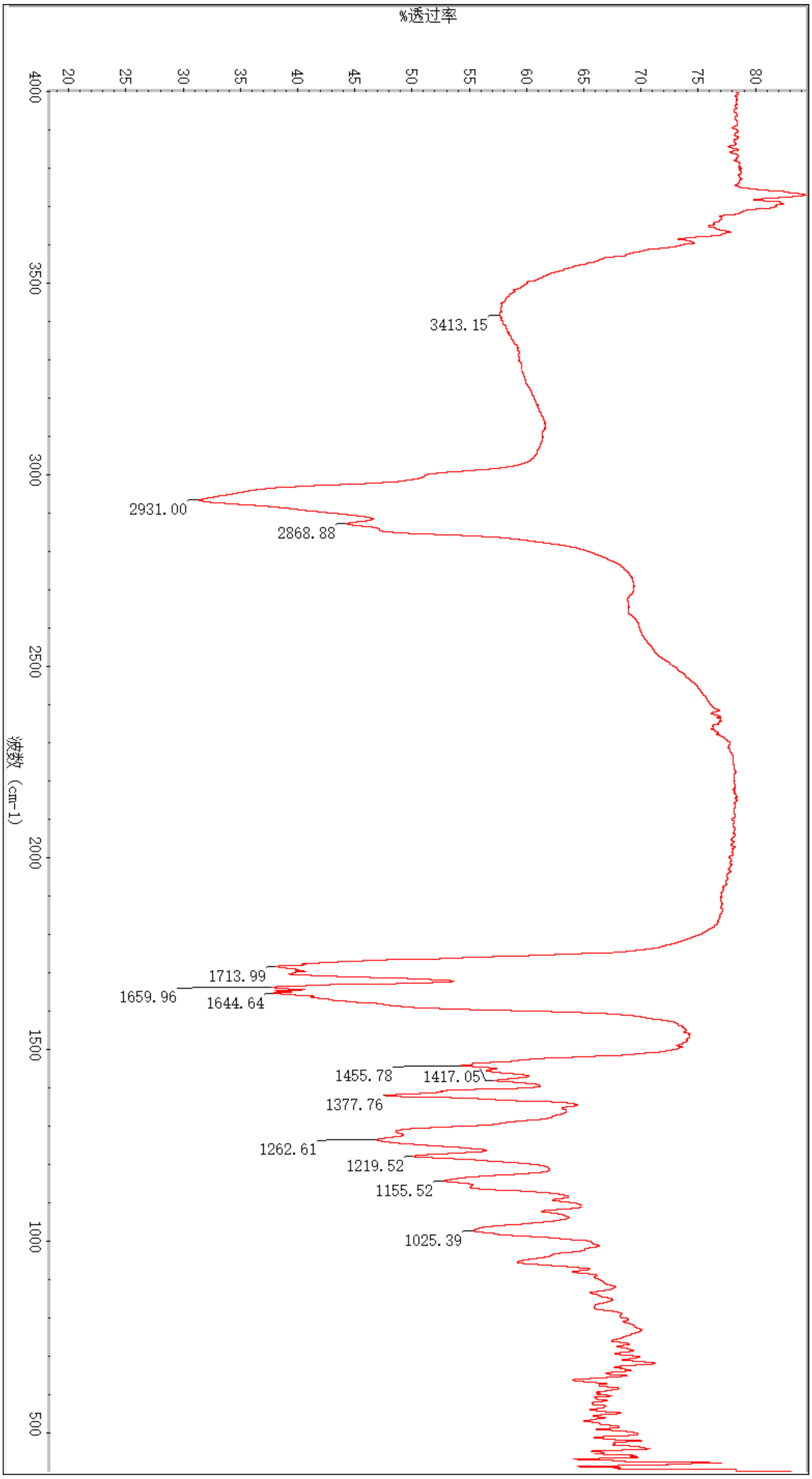

Figure S33. ^{1}H NMR spectrum of **5** in $(CD_3)_2CO$ (500 MHz)

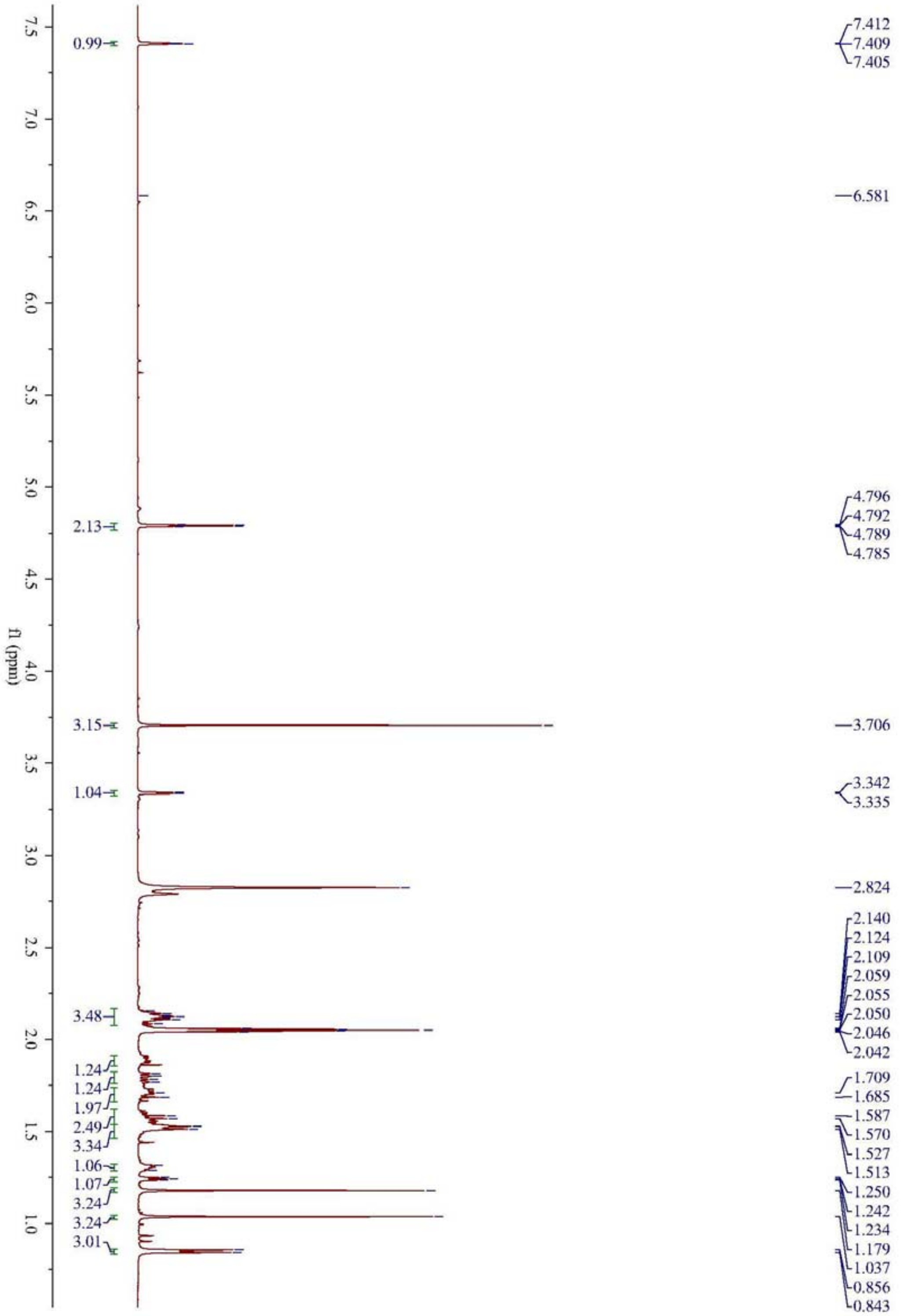

Figure S34. ^{13}C NMR spectrum of **5** in $(CD_3)_2CO$ (125 MHz)

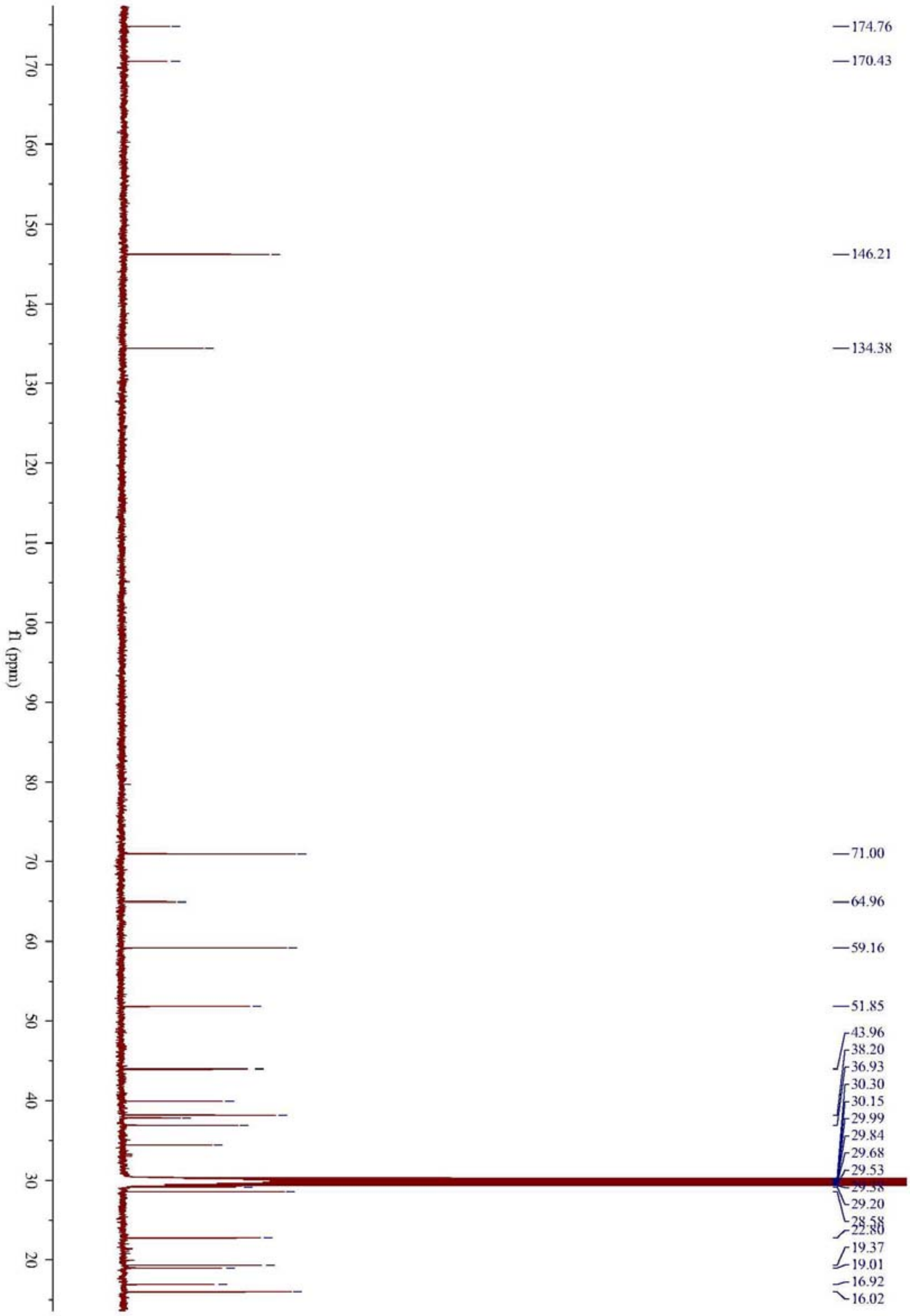

Figure S35. HSQC spectrum of **5** in $(CD_3)_2CO$

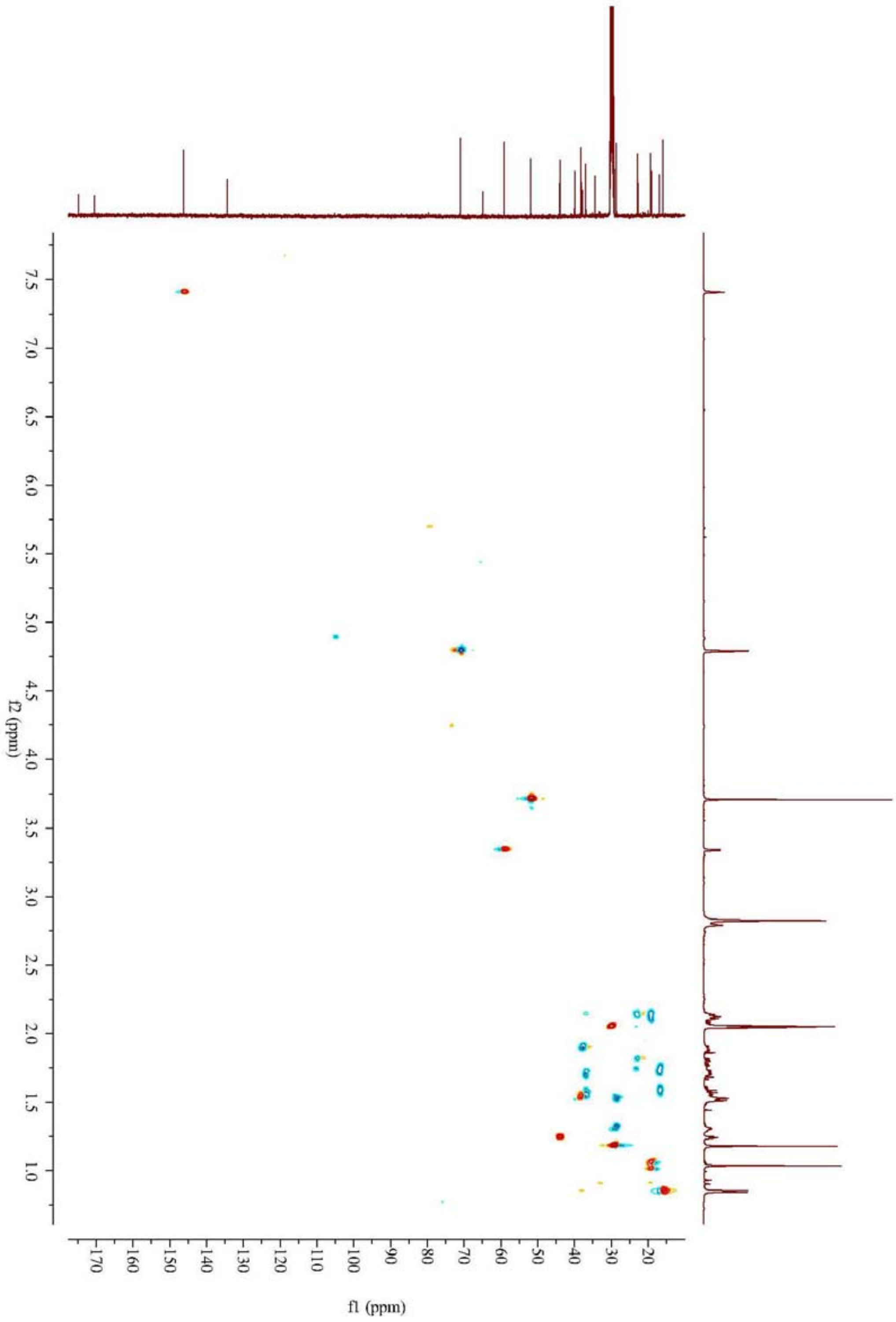

Figure S36. HMBC spectrum of **5** in $(CD_3)_2CO$

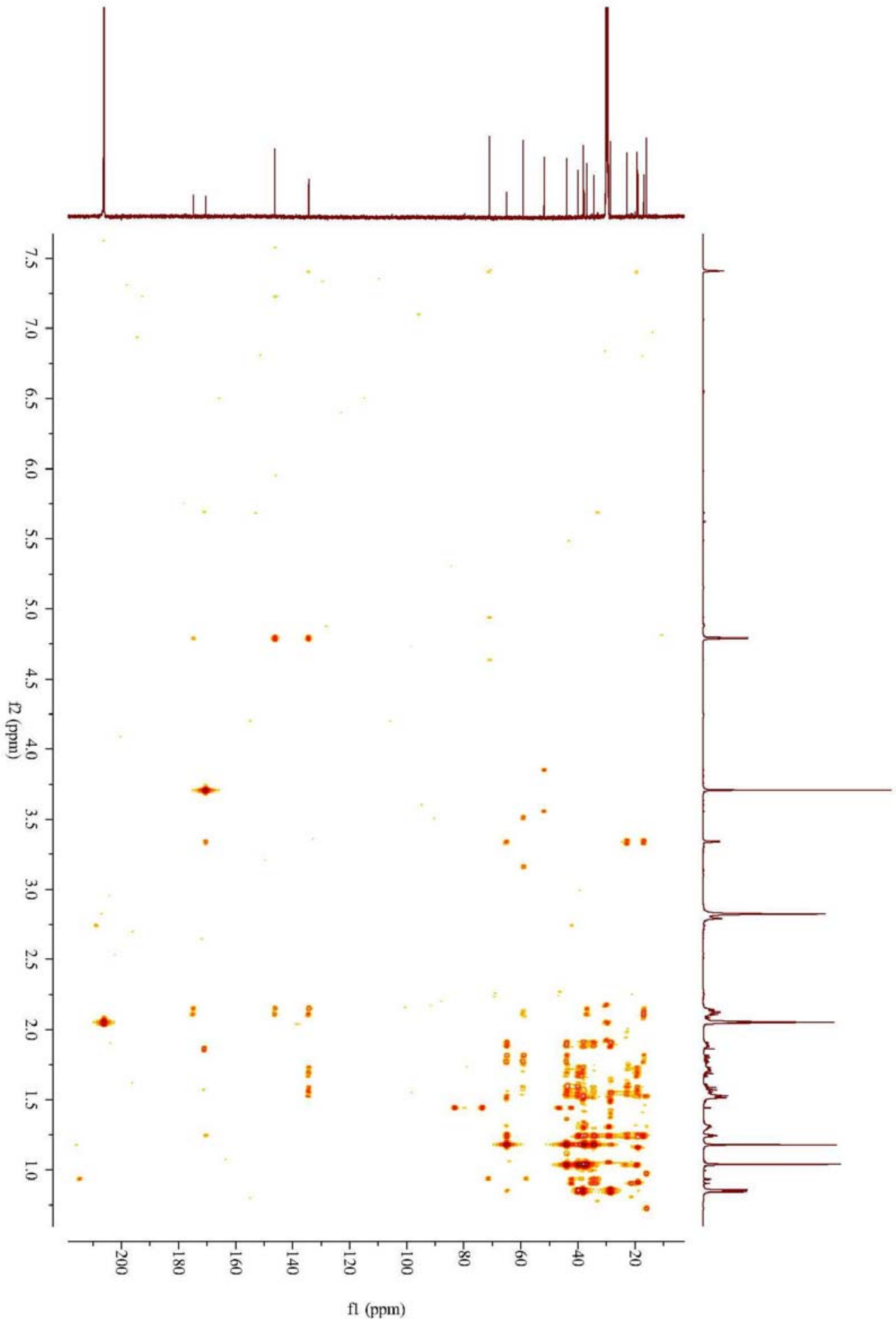

Figure S37. ^{1}H-^{1}H COSY spectrum of **5** in $(CD_3)_2CO$

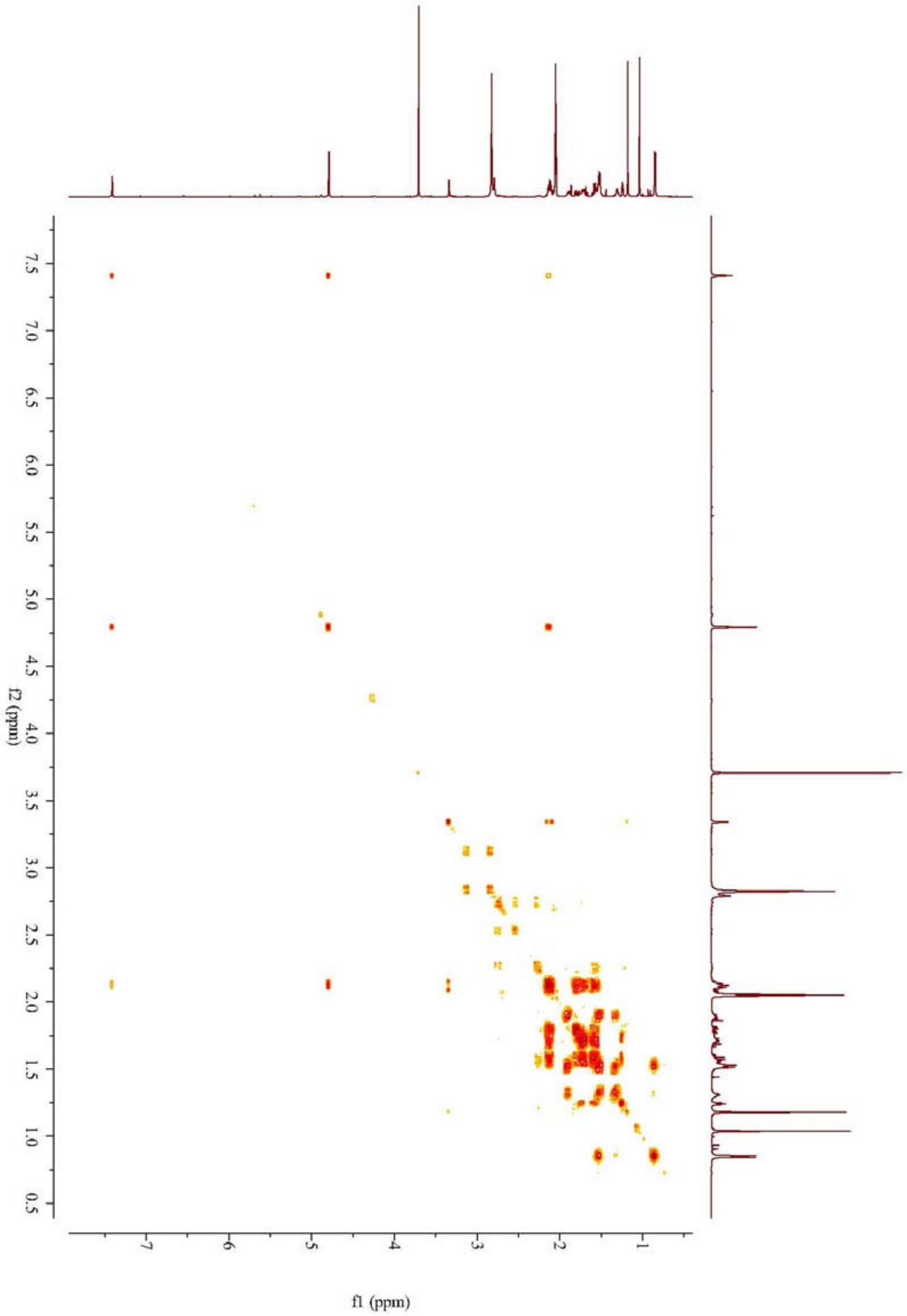

Figure S38. ROESY spectrum of **5** in $(CD_3)_2CO$

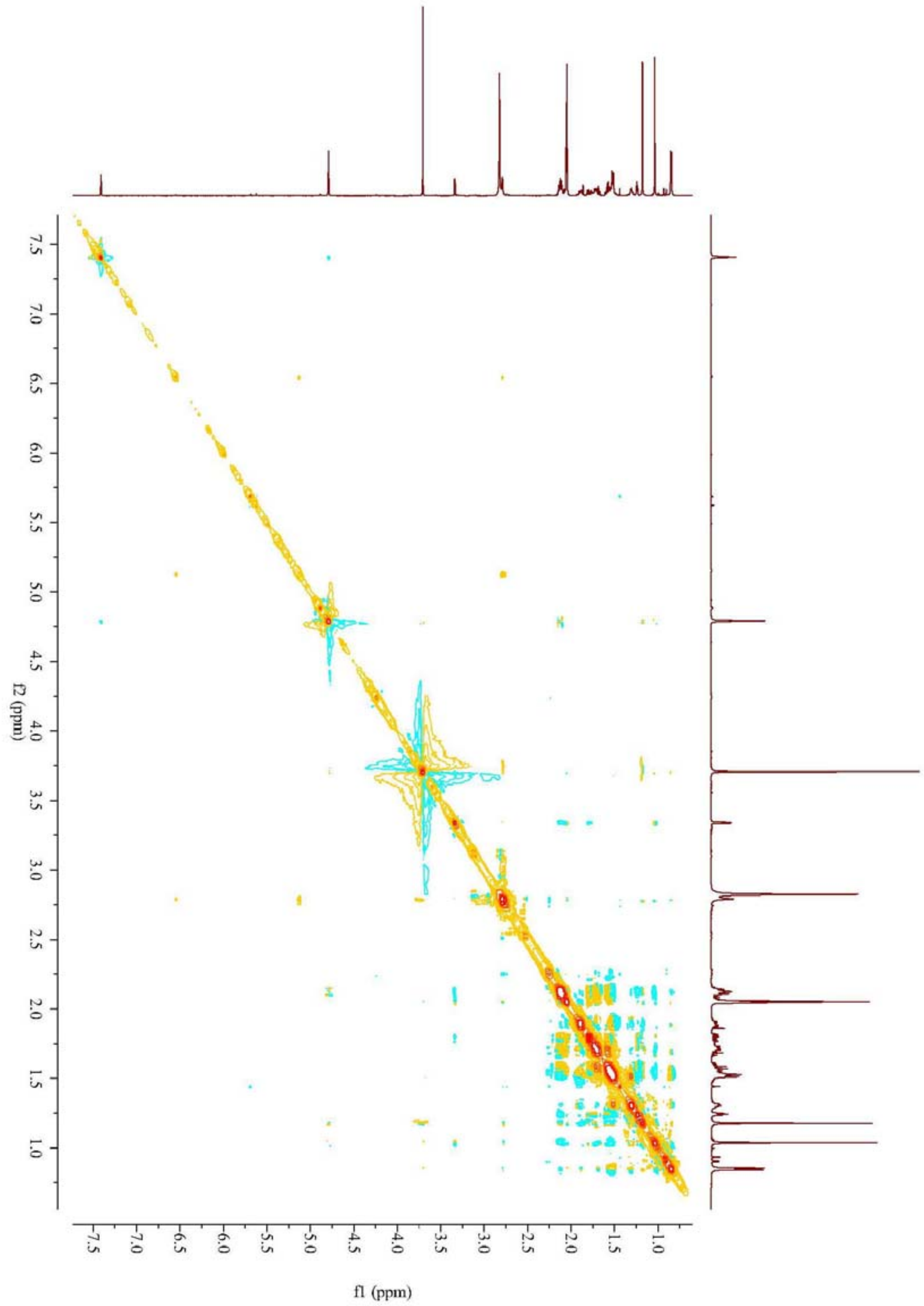

Figure S39. HRESIMS spectrum of **5**

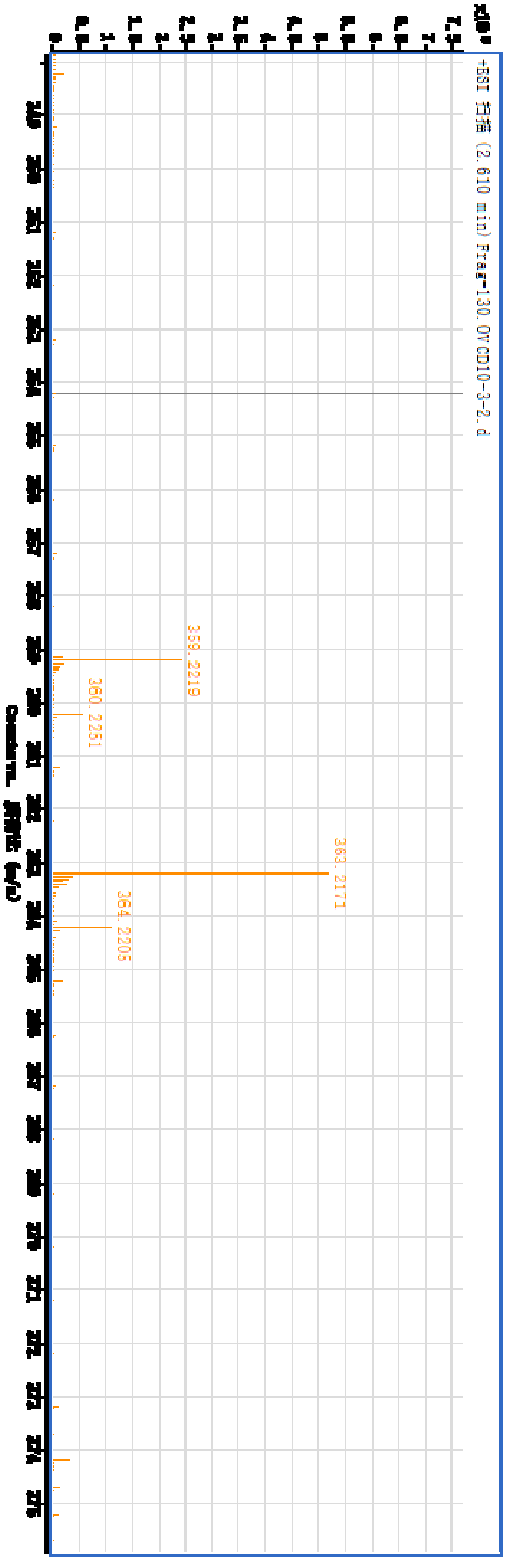

Figure S40. IR spectrum of **5**

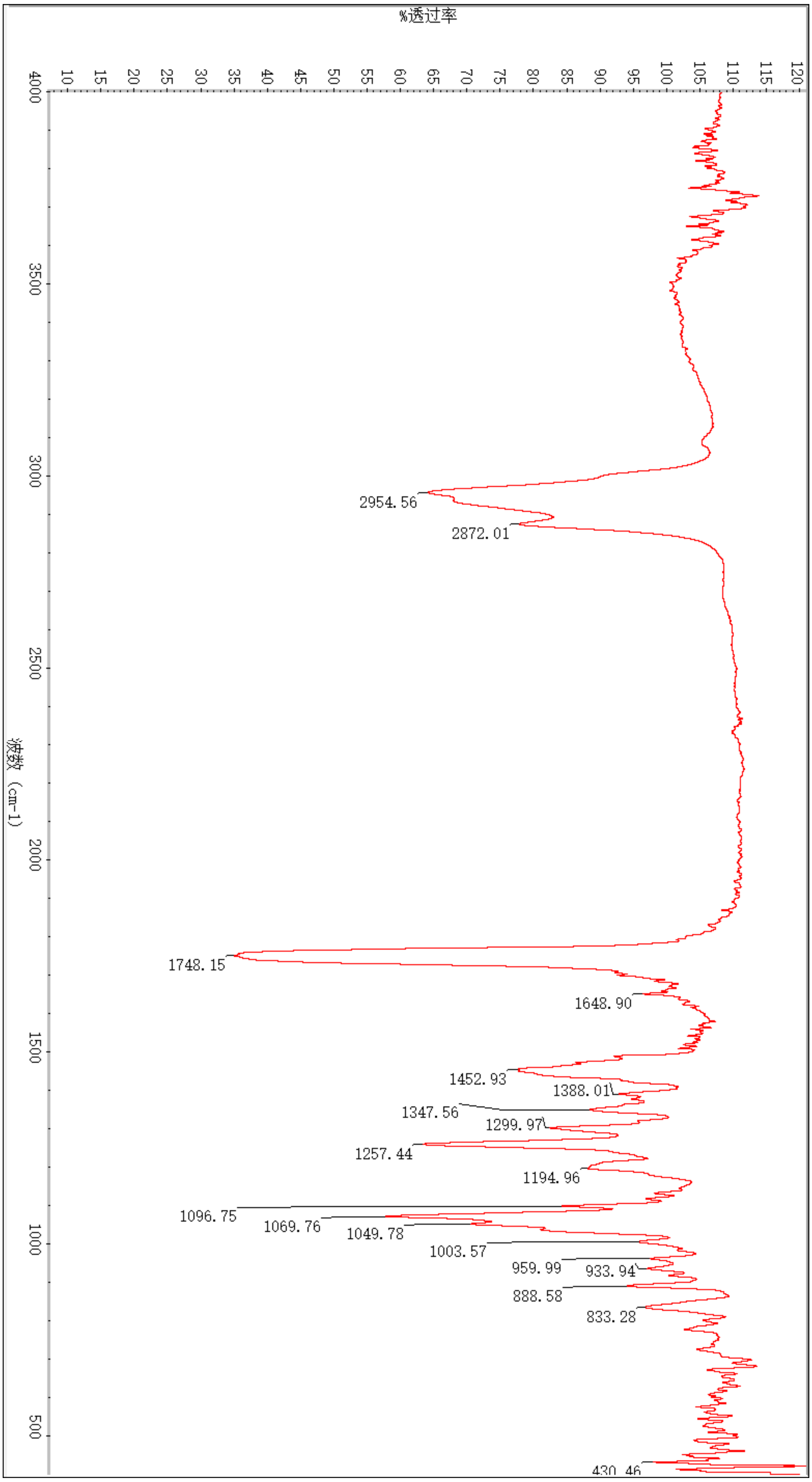

Figure S41. ^{1}H NMR spectrum of **6** in CD_3OD (500 MHz)

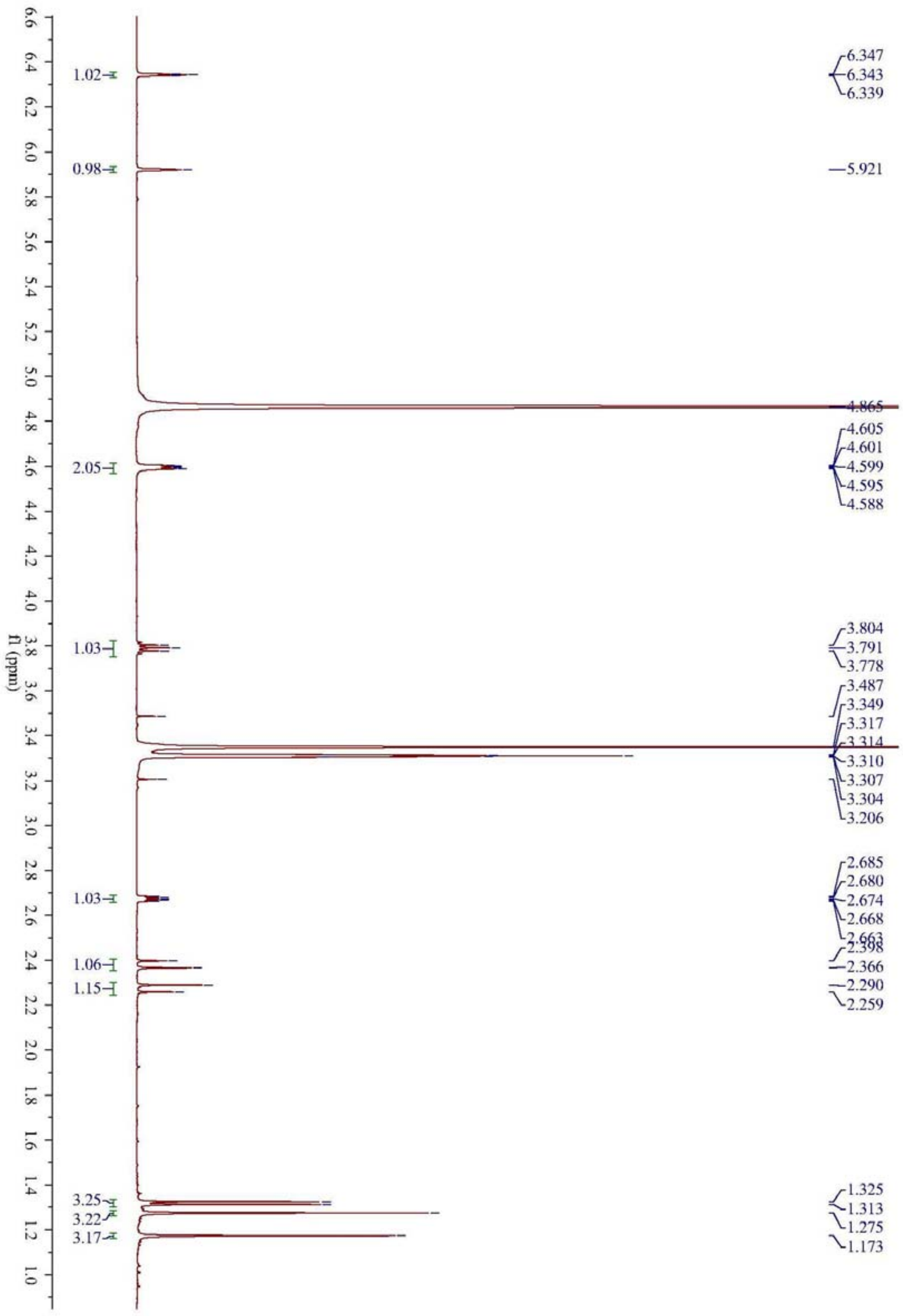

Figure S42. ^{13}C NMR spectrum of **6** in CD_3OD (125 MHz)

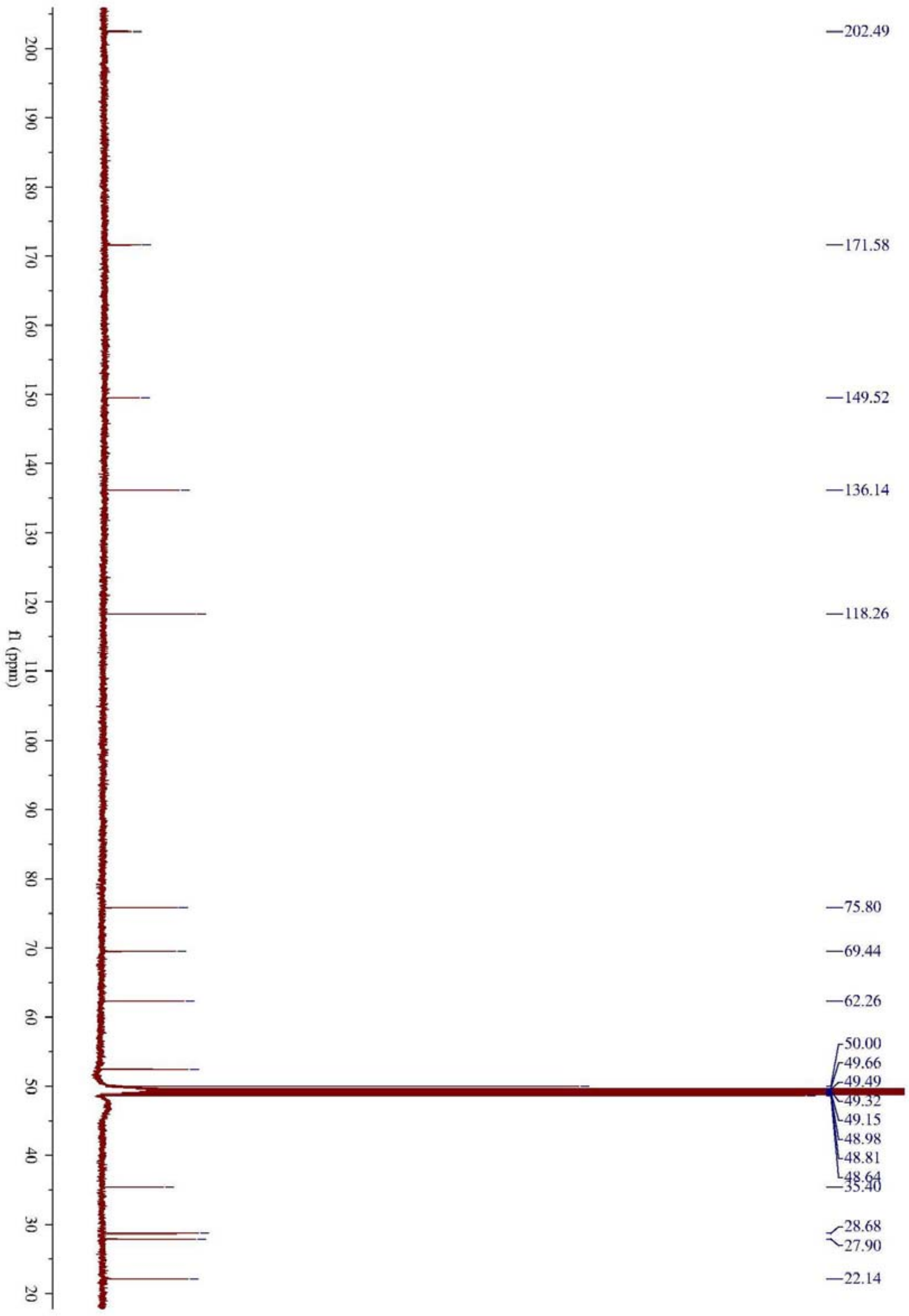

Figure S43. HSQC spectrum of **6** in CD_3OD

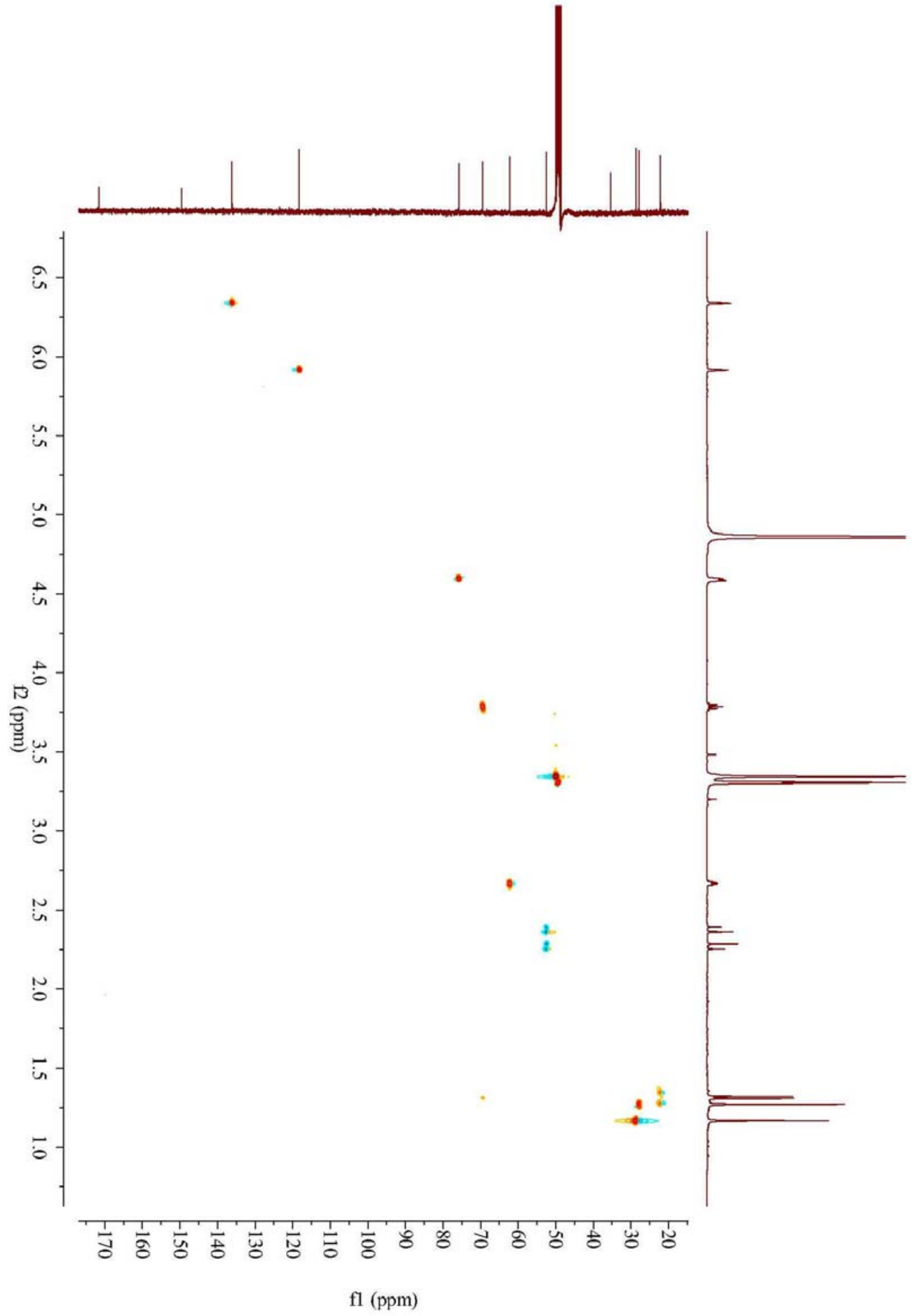

Figure S44. HMBC spectrum of **6** in CD_3OD

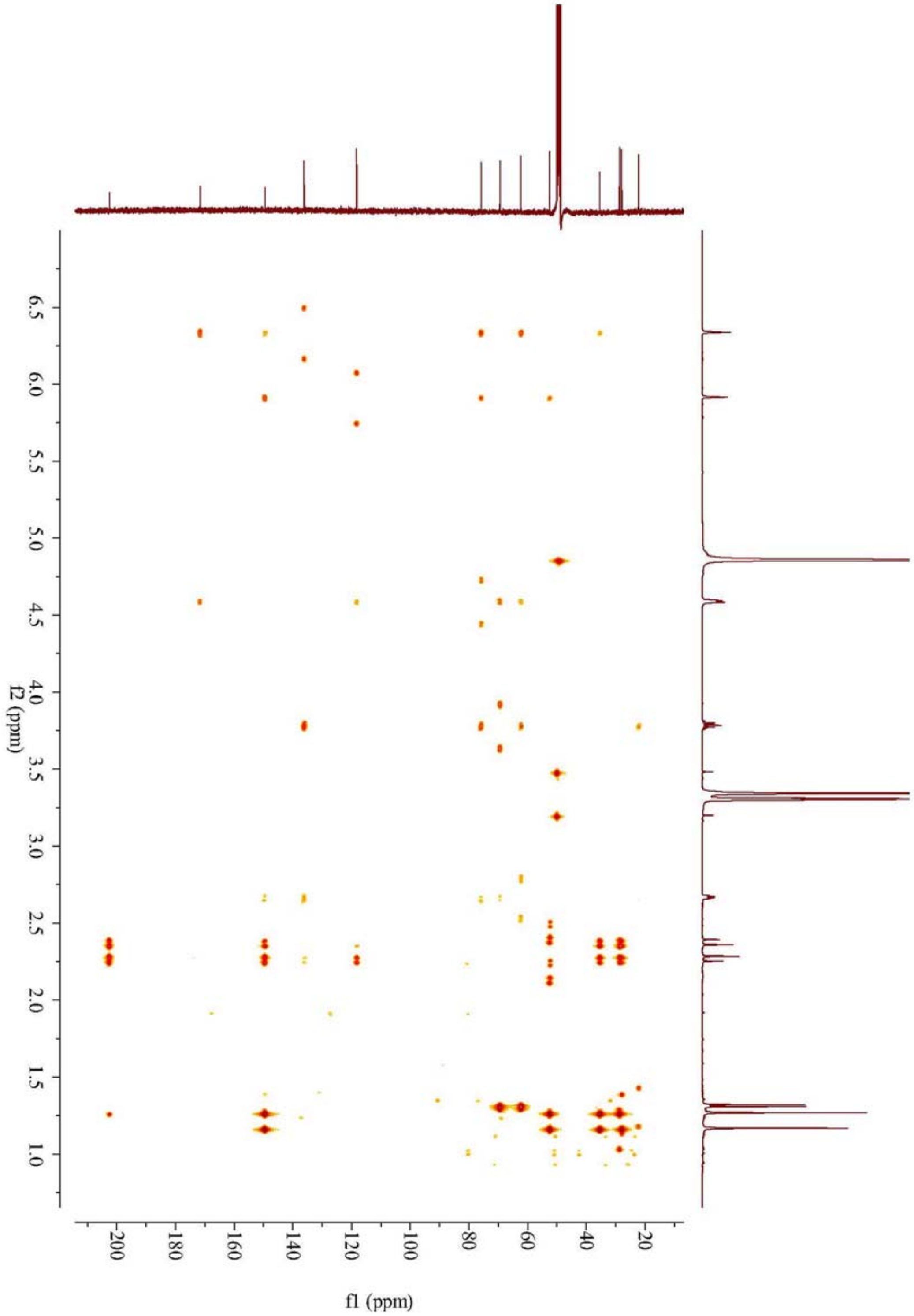

Figure S45. ^{1}H-^{1}H COSY spectrum of **6** in CD_3OD

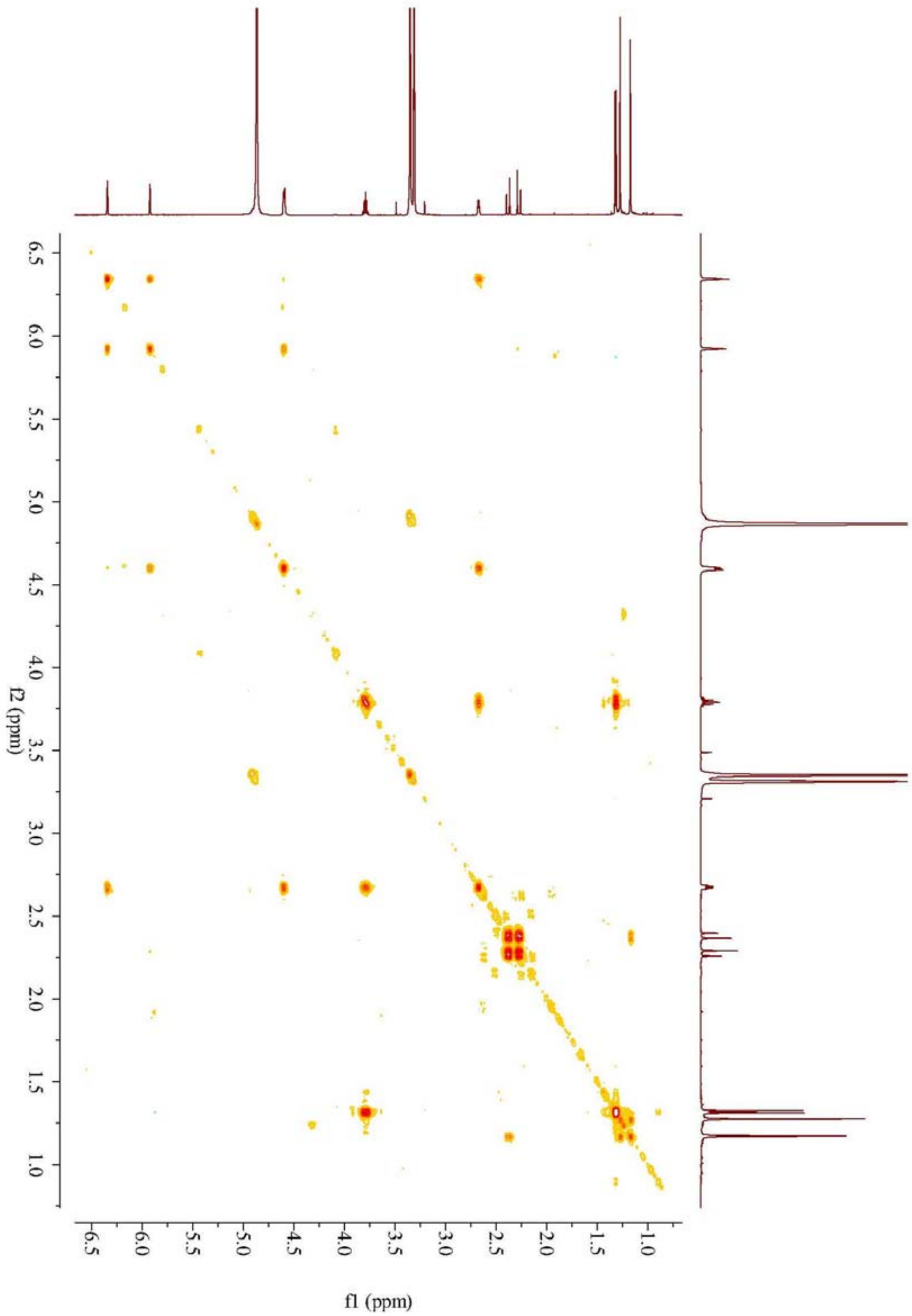

Figure S46. ROESY spectrum of **6** in CD_3OD

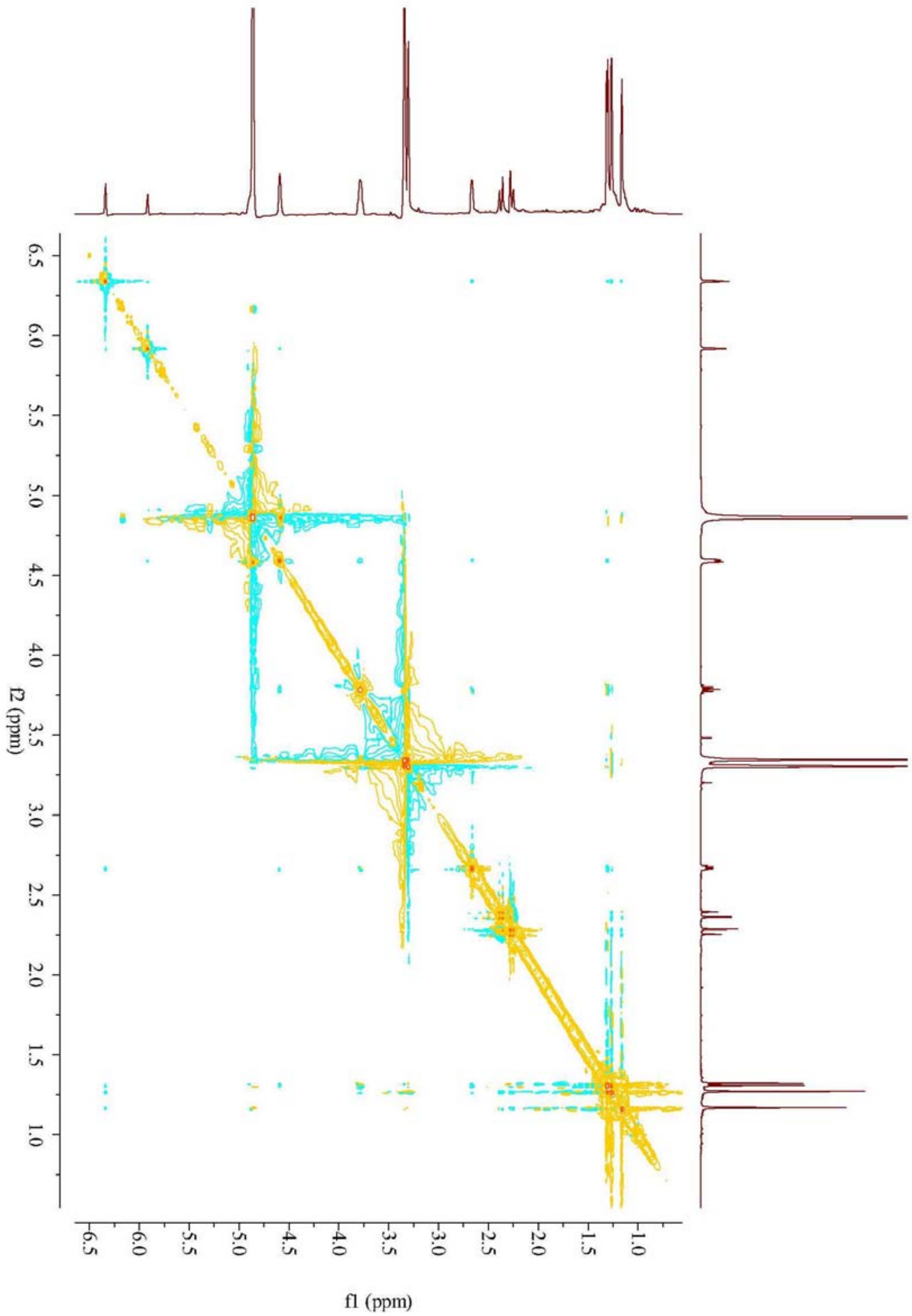

Figure S47. HRESIMS spectrum of **6**

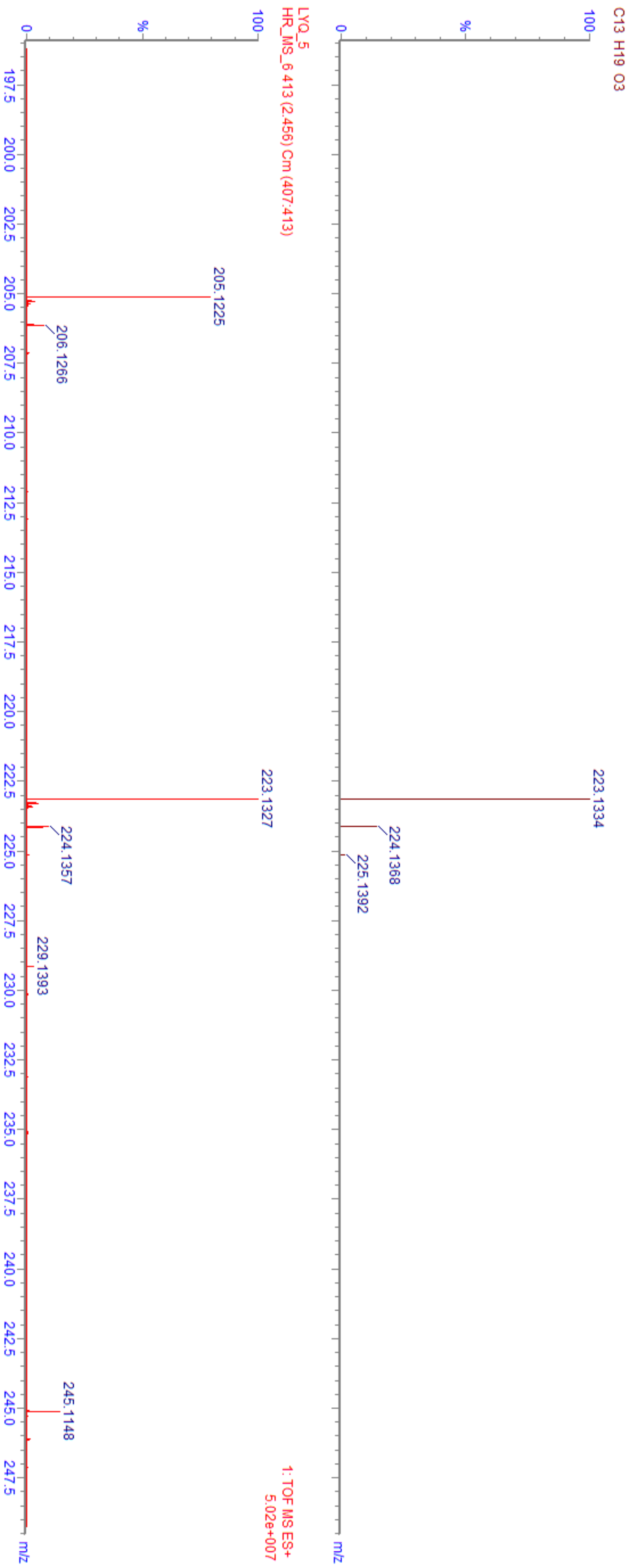

Figure S48. IR spectrum of **6**

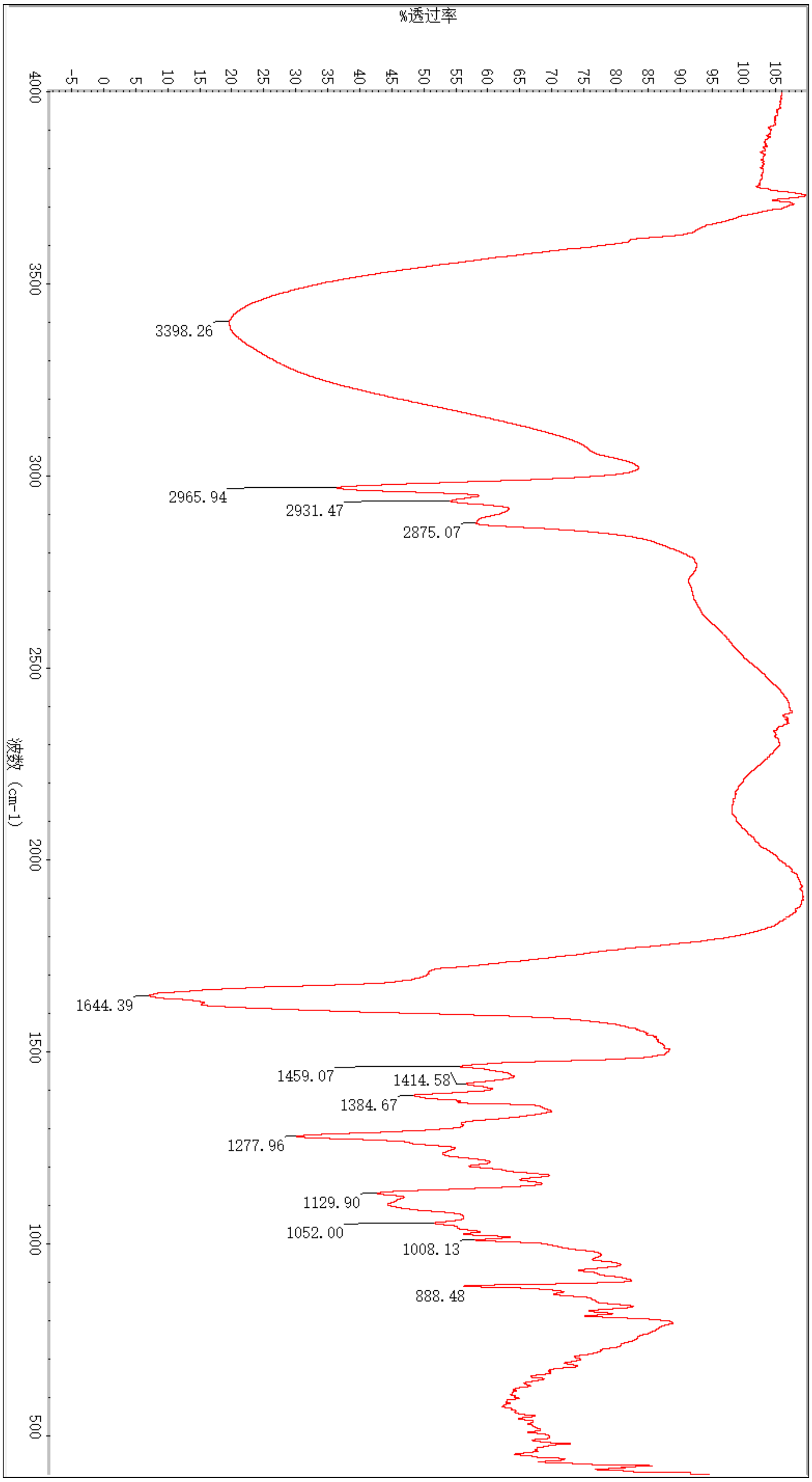

Supplement: Supplementary file 1 [file DataSheet1.pdf]
